# Palladium-catalysed synthesis of small-molecule epigenetic inhibitors as anticancer therapeutics

**DOI:** 10.1080/14756366.2026.2621477

**Published:** 2026-02-16

**Authors:** Ram Sharma, Mandeep Rana, Amandeep Thakur, Ritu Ojha, Seyyed Mojtaba Mousavi, Ashwani Dhingra, Kunal Nepali

**Affiliations:** ^a^ School of Pharmacy, College of Pharmacy, Taipei Medical University, Taipei, Taiwan; ^b^ Department of Optometry, Mackay Medical University, New Taipei City, Taiwan; ^c^ Department of Chemical Engineering, National Taiwan University of Science and Technology, Taipei, Taiwan; ^d^ Global Research Institute of Pharmacy, Global Research Group of Institutions, Yamuna Nagar, India; ^e^ Ph.D. Program in Drug Discovery and Development Industry, College of Pharmacy, Taipei Medical University, Taipei, Taiwan

**Keywords:** C–C/C–N bond formation, Palladium-catalysed cross-coupling, Small-molecule anticancer therapeutics, epigenetic targets, green catalysis

## Abstract

Palladium-catalysed reactions have emerged as indispensable tools in medicinal chemistry, enabling the precise construction of C-C and C-N bonds across a wide spectrum of drug-like molecular frameworks. This manuscript comprehensively examines advances reported over the past five years in palladium-catalysed methodologies applied to epigenetic drug discovery. The mechanistic diversity and synthetic adaptability of palladium catalysts for accessing scaffolds addressing the epigenetic targets have been highlighted. The robust drug design strategies and activity profile of the generated small molecule epigenetic inhibitors through palladium-assisted synthetic protocol are also presented in this compilation. Particular emphasis is placed on understanding the influence of ligand structure, base selection, and solvent optimisation in modulating catalyst reactivity. Collectively, this review offers a practical and forward-looking framework for the design and synthesis of next-generation epigenetic anticancer therapeutics (selective/non-selective/hybrid-inhibitors and degraders/PROTACS).

## Graphical Abstract

**Figure:**
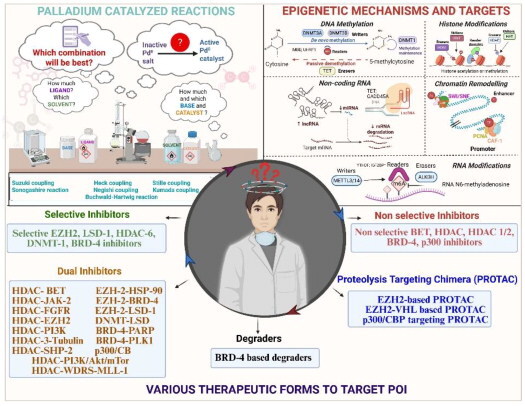

## Introduction

**Figure 1.**
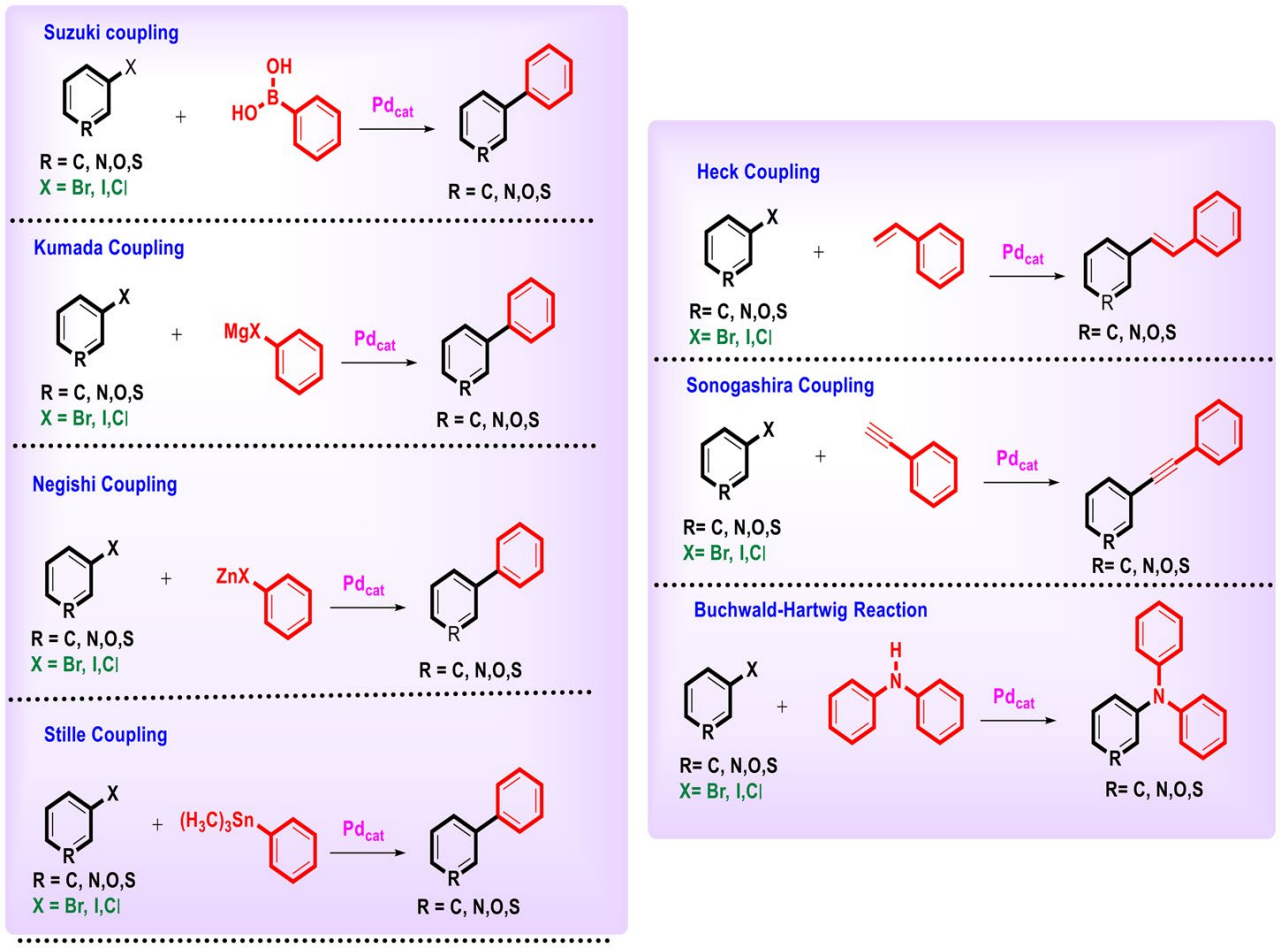
Key Pd-catalysed cross-coupling reactions for C–C and C–N bond formation (the figure was drawn by the authors using chemdraw software).

The pharmaceutical sector at present heavily relies on the applications of cross-coupling reactions for the construction of mechanistically diverse small molecule antitumor scaffolds. Palladium catalysis has emerged as the most prominent tool of the medicinal chemist’s toolbox to mediate the key steps (cross-coupling reactions) of the multistep synthetic protocols to anticancer structural templates^1–4^. Palladium-catalysed cross-coupling reactions (Figure 1) enable the synthesis of complex building blocks and intermediates with structural motifs common in many classes of APIs in a highly efficient and selective manner^2^^,^^3^. Notably, palladium-based catalysts have been most efficiently employed for the synthesis of biaryl constructs as antitumor templates by the Suzuki-Miyaura cross-coupling reaction between organic boron compounds and organic halides. It is important to mention that the palladium-catalysed Suzuki-Miyaura cross-coupling reaction is a versatile C-C bond formation reaction that offers significant flexibility of application to various substrates^5^. In addition, vinylation of aryl halides to afford substituted alkenes (sp^2^-sp^2^ carbon cross-coupling reaction, Heck coupling) and sp-sp^2^ carbon cross-coupling reaction of terminal alkynes with aryl halides (Sonogashira coupling) to generate substituted alkynes has also played an enormously decisive and important role in shaping chemical synthesis of pharmaceutically important structural templates^6^. Also, palladium-catalysed cross-coupling of organic electrophiles (Ar-X) with Grignard reagents (Kumada coupling), organostannic reagents (Stille), and organozinc reagents (Negeshi) represent selective and heavily used C-C bond-forming reactions. For the aforementioned reactions, the carbon atom bound to the boron, magnesium, zinc, and stannous could be sp, sp^2^, and sp^3^ hybridised^6^. In the context of C-N bond formation, the generation of arylamines and heteroarylamines via palladium-catalysed Buchwald–Hartwig reaction is one of the most comprehensively leveraged reactions (Figure 1) that has been utilised to establish the synthetic routes of numerous medicinally active agents. Specifically, it is a cross-coupling reaction between an aryl/heteroaryl halide with a primary or secondary amine in the presence of a palladium metal catalyst and a base^1^. A careful selection of catalysts and conditions in this cross-coupling reaction enables selective arylation of primary over secondary amines and vice versa, as well as the incorporation of heterocyclic substrates and sterically hindered substrates^7^. Delightfully, palladium catalysis for C-C/C-N bond assemblage is extremely operative both on bench and industrial scale as it enables the chemist to generate bioactive scaffolds at a scale that ranges from milligram to multiton^8–13^.

The ongoing wave in the field of catalytic chemistry has been inclined towards the quest to expand the list of palladium catalysts, and the chemist has demonstrated utmost proficiency in this context as the armoury of palladium-based catalysts has been supplemented with quite a few entries lately (Table 1). The aforementioned efforts of the organic chemist have most significantly benefitted the medicinal chemistry teams, as they are always on the lookout for efficient and tractable catalysts that can expedite the overall multistep synthetic route and produce the target adducts in good yield. The accessibility to a large pool of palladium catalysts with proven catalysis potential is a privilege for the drug discovery groups, as it enables them to conduct comprehensive optimisation of the synthetic methodologies to pinpoint a specific palladium catalyst on the basis of the chemical nature of the scaffold for the stitching of C-C/C-N bond. It is often believed that the success of a drug discovery campaign relies on the strategies leveraged to design the antitumor templates that can interact with the amino acid residues of the target proteins. There is no denying the fact that the drug design approach holds significant importance in medicinal chemistry endeavours; however, the synthetic routes developed to furnish the designed chemical architectures must be given tantamount consideration to translate the drug discovery enthusiast’s envisionment to reality. Notably, the reliance of synthetic routes to be categorised as efficient ones on the ability of the catalyst to furnish the intermediatory adducts is quite evident from the literature precedents, which underscores the importance of the catalyst in drug discovery. Moreover, in-depth investigations, including *in vivo* characterisations of the synthesised adducts, are only feasible if the developed synthetic protocols are able to produce the target compounds in acceptable yields. In this context, the continued emergence of palladium-based catalysts has proved to be a boon for the drug discovery teams.

**Table 1.** List of Various organopalladium catalysts.

Organopalladium Catalyst	Type of bonds Formed	Name of the reactions	Structure	Characteristics	Ref
Palladium(II) Chloride (PdCl_2_)	C-C	Suzuki & Heck coupling reactions, Wacker reaction	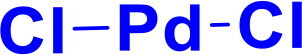	Palladium (II) chloride serves as a precursor for heterogeneous catalysts, electronic materials, speciality polymers, and palladium nanoparticles, which are used in sensors and catalysis.	^18–21^
Dichlorobis(triphenylphosphine)palladium(II) PdCl_2_(Ph_3_P)_2_	C-C C-N	Heck intramolecular reactions Suzuki-Miyaura Coupling Stille Coupling Carbonylation Reactions Buchwald-Hartwig animation Sonogashira reaction Negishi Coupling	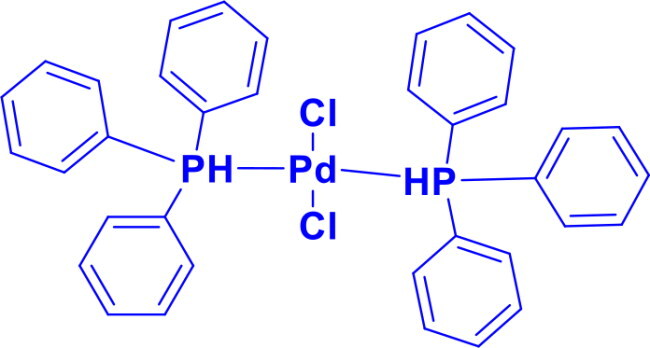	Triphenylphosphine ligands stabilise the palladium centre by providing electron density, enhancing its reactivity towards oxidative addition, and facilitating cross-coupling of terminal alkynes with aryl halides.	^22^ ^,^ ^23^
Bis(acetonitrile)dichloropalladium(II) PdCl_2_(CH_3_CN)_2_	C-O C-N	Buchwald Hartwig amination Suzuki-Miyaura coupling, Stille coupling, Sonogashira coupling, Negishi coupling, Hiyama coupling, Heck reaction Hegedus Indole synthesis	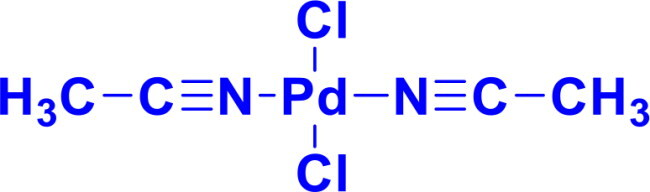	The acetonitrile ligands in PdCl₂(CH₃CN)₂ are weakly bound and easily displaced, making it a versatile precursor in organometallic chemistry, which allows faster reactions and low ligand loading PdCl_2_(CH_3_CN)_2_ is known as an environmentally friendly catalyst	^1–5^ ^,^ ^19^
[1′-Bis(diphenylphosphino)ferrocene]palladium(II) dichloride PdCl_2_(dppf)	C-N C-C	Buchwald Hartwig amination Suzuki coupling	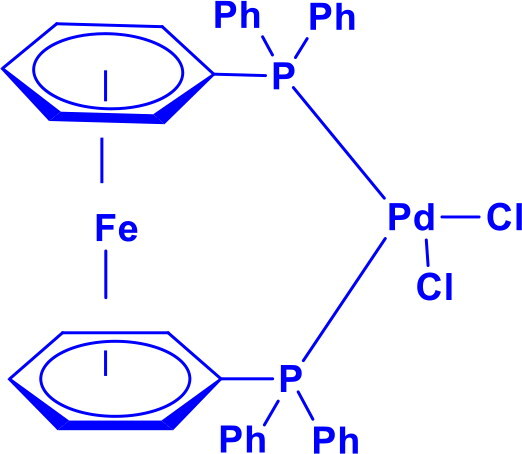	Pd(dppf)Cl_2_ catalyst is more robust and storable dppf ligand provides steric and electronic control Works efficiently at lower temperatures Compatible with aryl, vinyl, and alkyl electrophiles	^6^ ^,^ ^7^
Dichloro[4-bis(diphenylphosphino)butane]palladium(II) PdCl_2_(dppb)	C-C	Kumada Coupling Suzuki-Miyaura Coupling Negishi coupling Suzuki coupling Sonogashira coupling	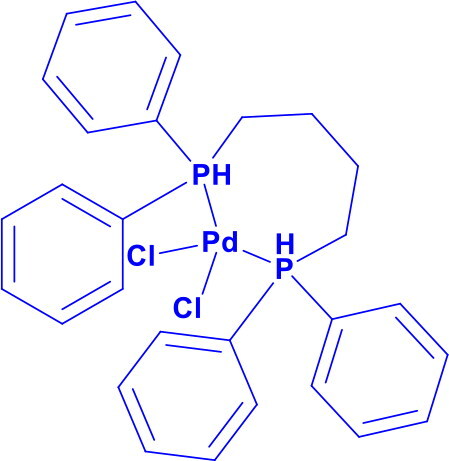	It provides better control over regio- and stereoselectivity due to the rigid coordination environment, poor solubility in organic solvents due to the variable bite angle	^24^ ^,^ ^25^
[1,1-Bis(di-tert-butylphosphino)ferrocene]dichloropalladium(II) PdCl₂(dtbpf)	C-C C-N	Buchwald Hartwig amination Suzuki coupling Hiyama coupling Sonogashira reaction Heck coupling Stille coupling	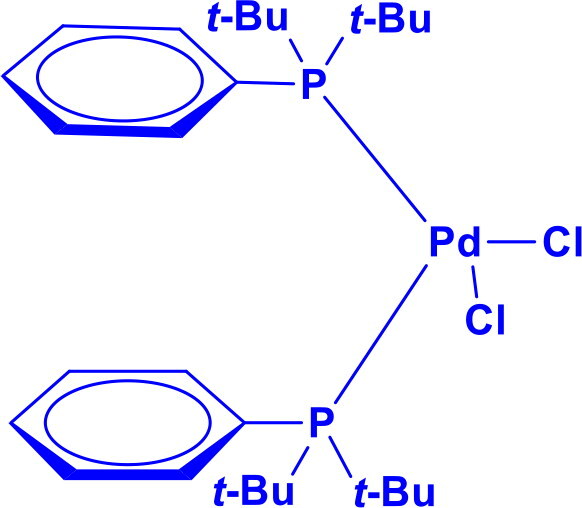	Bulky di-tert-butyl groups increase solubility and stability	^26^
Tetrakis(triphenylphosphine)palladium(0) Pd(Ph_3_P)_4_	C-C C-N C-O C-P	Heck reaction, Suzuki coupling, Stille coupling, Sonogashira coupling, and Negishi coupling Stille–Kelly coupling Kumada coupling	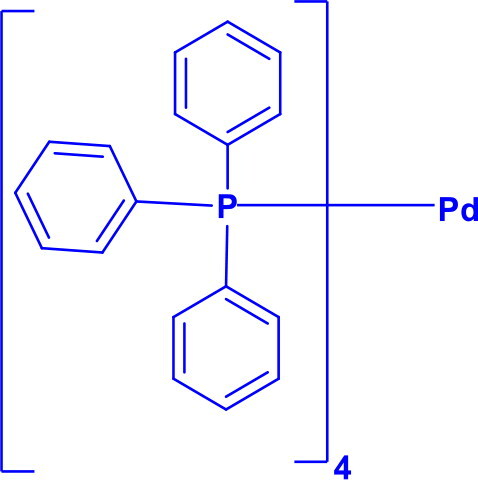	Unique character of Pd(PPh_3_)_4_ is soluble in non-polar solvent Directly provides Pd(0) active species Catalytic selectivity is high, but it generates steric hindrance from the PPh₃ ligands	^27–30^
Bis(dibenzylideneacetone)palladium(0) Pd(dba)_2_	C-N	Buchwald-Hartwig Cross-Coupling Wacker-type reaction	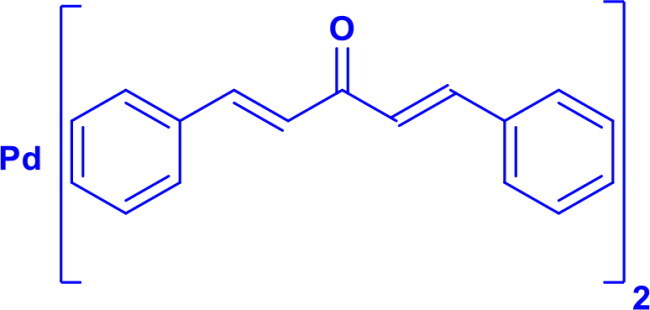	Consists of a dimeric Pd(0) Complex with two bridging dba Ligands	^31–36^
Tris(dibenzylideneacetone)dipalladium(0) Pd_2_(dba)_3_	C-N C-S	Buchwald Hartwig amination Heck Coupling	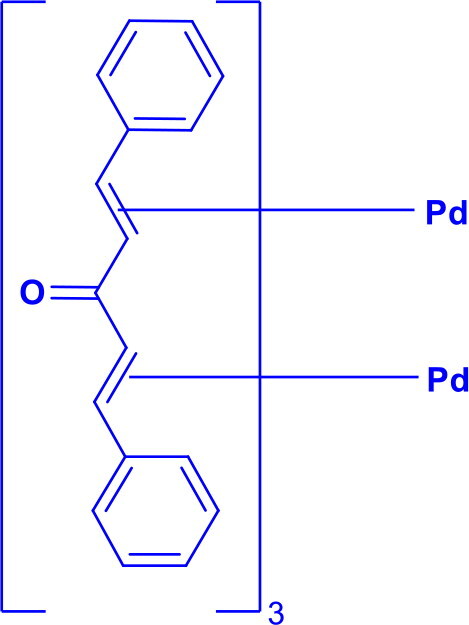	Consists of a dimeric Pd(0) Complex with bridging three dba Ligands Pd_2_(dba)_3_ can easily exchange ligands	^31–36^
Dichlorobis(tri-o-tolylphosphine)palladium(II) PdCl_2_[P(*o* -tol)_3_]_2_	C-N C-C	Buchwald-Hartwig animation Stille Coupling Heck Couling	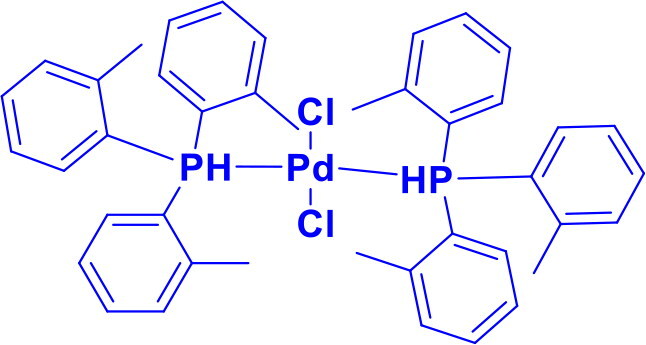	P(*o*-tol)_3_ ligand is bulky and provides steric hindrance that influences the selectivity and reactivity of the catalyst.	^37^
allylpalladium(ii) chloride dimer Pd(η³-C₃H₅)Cl)₂	C-C	Heck reactions Suzuki-Miyaura reactions Tsuji-Trost reactions		η³-allyl ligand provides stability and is highly effective in allylic substitution reactions	^38^
Palladium(II)cyanide Pd(CN)_2_	C-C	Suzuki-Miyaura reaction Cyanation Reactions	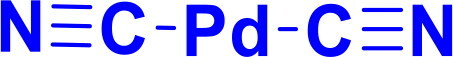	The cyanide ligand forms strong σ-donor and π-acceptor bonds with palladium, stabilising Pd(CN)₂ and influencing its reactivity, though its poor solubility in organic solvents limits use in homogeneous catalysis.	^39^ ^,^ ^40^
Palladium(II) trifluoroacetate Pd(OCOCF_3_)_2_	C-C C-N	Suzuki coupling Heck Reaction Sonogashira reaction Buchwald Hartwig amination Allylic Oxidation Epoxidation	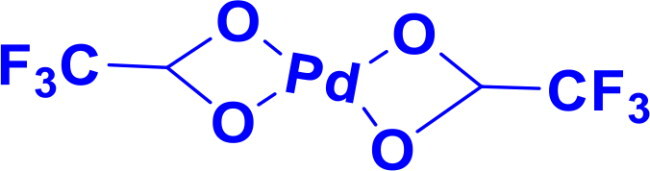	The trifluoroacetate ligands in Pd(OC(O)CF₃)₂ are highly electron-withdrawing due to the strong inductive effect of the fluorine atoms, making the palladium centre more electrophilic. This enhances its reactivity in catalytic processes like cross-coupling reactions compared to Pd(OAc)_2_	^39–42^
N-heterocyclic carbene palladium(II) diacetate complex [(NHC)Pd(OAc)_2_]	N-C(O)	Suzuki-Miyaura and Buchwald-Hartwig	[(NHC)Pd(OAc)_2_]	Bench-stable, well-defined Pd(II)–NHC precatalyst that is air- and moisture-tolerant. Acetate ligands dissociate readily to generate the catalytically active Pd–NHC species. Strong σ-donating NHC ligand facilitates oxidative addition into the amide N–C(O) bond.	^42^
Palladium(II) acetate Pd(OAc)_2_	C-N C-C C-O C-H	Buchwald Hartwig amination Suzuki coupling Hiyama coupling Sonogashira reaction Heck coupling The wacker-type reactions	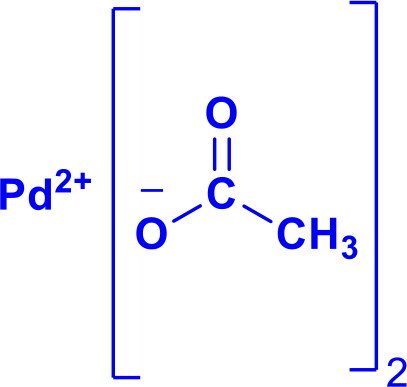	Pd(OAc)₂ is more versatile in organic solvents and is preferred for C–H activation, cross-coupling, and oxidation reactions. Used for the synthesis of Furan	^13^ ^,^ ^43^
Bis(triphenylphosphine)palladium(II) diacetate Pd(PPh_3_)_2_(OAc)_2_	C-C C-O C-N Indole synthesis	Heck reaction Suzuki-Miyaura coupling Sonogashira coupling Buchwald-Hartwig amination	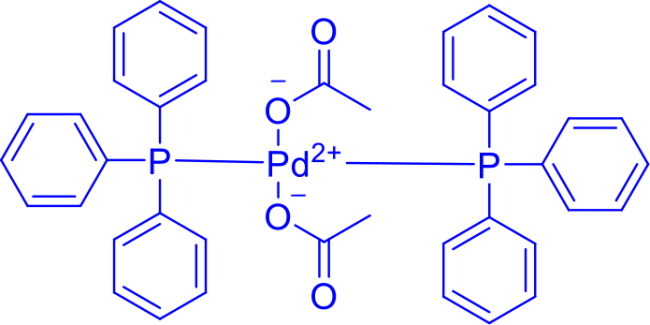	Highly effective catalyst for the Larock indole synthesis Used for the synthesis of indole derivatives for drug discovery (e.g., serotonin receptor modulators, kinase inhibitors) The dual ligand system (OAc and PPh₃) provides a balance of reactivity and stability.	^43–45^
Palladium(II) acetylacetonate C_10_H_16_O_4_Pd	C-C	Suzuki-Miyaura coupling Heck reaction Sonogashira coupling Stille coupling	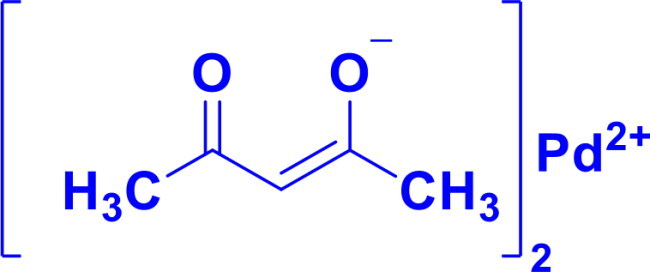	Palladium(II) acetylacetonate is air-stable, soluble in organic solvents, and features bidentate acetylacetonate ligands that stabilise the palladium centre.	^46^ ^,^ ^47^
Bis(benzonitrile) palladium(II) chloride PdCl_2_(NCC_6_H_5_)_2_	C-C C-H activation α-O-glycosylation	Suzuki-Miyaura coupling, Heck reaction, Sonogashira coupling, Stille coupling, Wacker Oxidation, Aza-Michael Reaction, Nazarov Cyclisation, Diamination of Conjugated Dienes	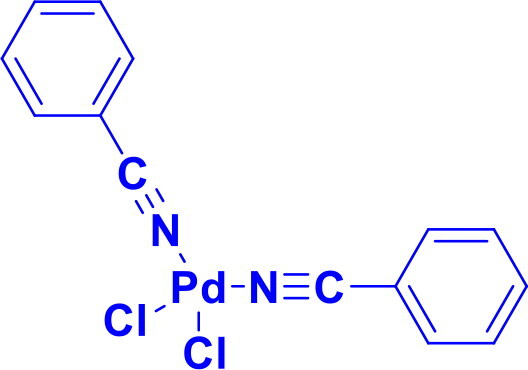	Easy displacement of PhCN ligands generates active catalytic species	^48^
Palladium(II) acetate supported by a bis(octadecyl)-DABCO dibromide ligand	C-C	Heck Coupling Suzuki–Miyaura cross-coupling	Pd(OAc)_2_/[C_18_–DABCO–C_18_]_2_Br	Forms stable Pd nanoparticles (<5 nm) in water, stabilised by the Gemini-type [C18–DABCO–C18]₂Br surfactant, Shows high efficiency and selectivity in Mizoroki–Heck and Suzuki–Miyaura couplings, with yields up to 95%, Works for a broad range of aryl iodides and bromides under mild conditions (80–100 °C, K₂CO₃ base), Recyclable for at least three cycles with only a slight decrease in catalytic activity.	^48^
Pd(OAc)_2_/CuI Complex (Palladium(II) acetate) and CuI (copper(I) iodide)	C-C	Sonogashira Coupling	Pd(OAc)_2_/CuI	Enables tandem C(sp³)–X activation and Sonogashira-type C(sp)–H coupling in a single sequence. Bimetallic Pd–Cu cooperation is crucial, especially during transmetallation, improving reactivity of otherwise inert C(sp³) centres. It provides a broad application of Sonogashira chemistry to β-lactam frameworks.	
Methanesulfonato(2-dicyclohexylphosphino-3,6-dimethoxy-2′,4′,6′-tri-i-propyl-1,1′-biphenyl)(2′-amino-1,1′-biphenyl-2-yl)palladium(II) BrettPhos Pd G_3_	C-N	Buchwald-Hartwig cross-coupling reaction	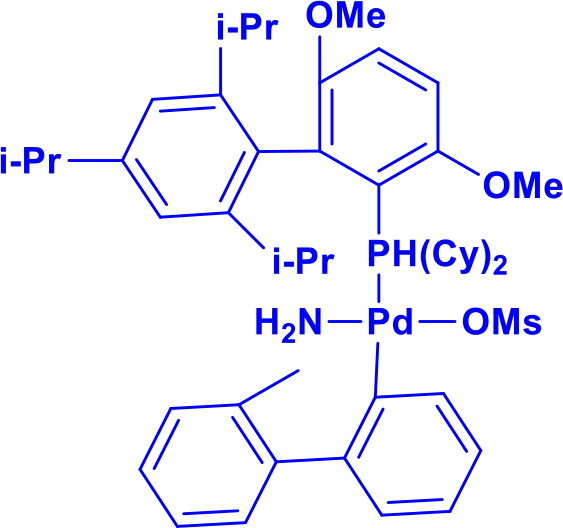	Stable to air, moisture, and heat, with excellent solubility in common organic solvents and long shelf life. Excellent catalysis for Buchwald-Hartwig cross-coupling	^49^ ^,^ ^50^
Methanesulfonato2-(dicyclohexylphosphino)-2′-(N,N-dimethylamino)-1,1′-biphenylpalladium(II) DavePhos-Pd-G3	C-C C-N	Suzuki-Miyaura Coupling, Buchwald-Hartwig Coupling, Heck Reaction, Hiyama Coupling, Negishi Coupling, Sonogashira Coupling, Stille Coupling	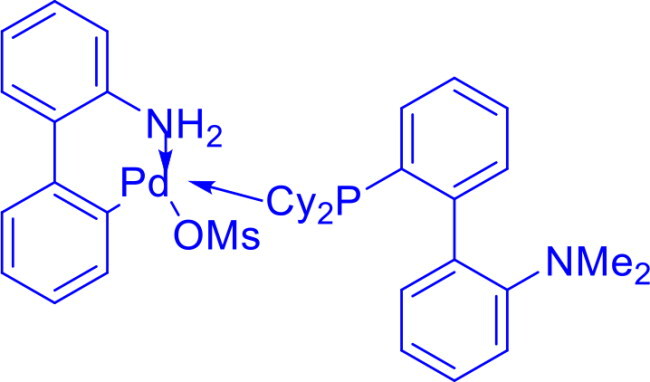	DavePhos-Pd-G3 features a bulky, electron-rich DavePhos ligand that enhances reactivity and stability, activates rapidly under mild conditions and is ideal for challenging couplings.	^51^
Dichloro1,3-bis(2,6-di-3-pentylphenyl)imidazol-2-ylidenepalladium(II), Pd-PEPPSI-IPent	C-N C-C	Stille coupling reaction Negishi coupling reaction Suzuki coupling reaction	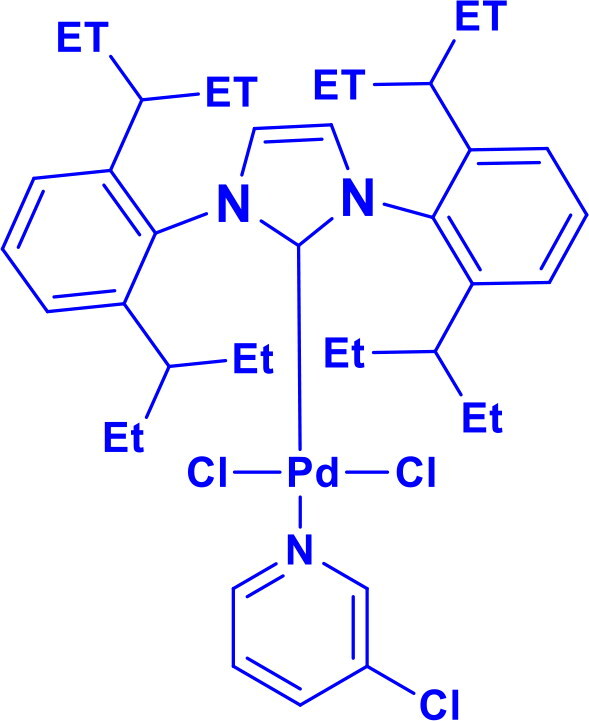	Pd-PEPPSI-IPent is an air-stable Pd(0) complex with a flexible PEPPSI ligand and an isopentyl group. It excels in C–C cross-coupling reactions like Suzuki and Buchwald-Hartwig, offering high catalytic efficiency, low catalyst loading, and minimal Pd aggregation.	^52^
Methanesulfonato9,9-dimethyl-4,5-bis(diphenylphosphino)xanthenepalladium(II) Xantphos Palladacycle Gen_4_ (R)-1-[(Sp)-2-(dicyclohexylphosphino)ferrocenyl]ethyldi-tert-butylphosphine	C-S	Palladium-catalysed thiolation reaction	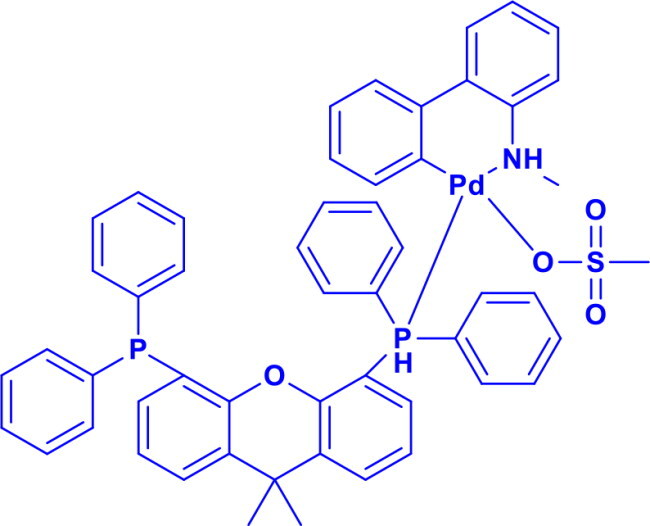	Highly efficient for C-S bond formation Highly stable at room temperature Highly reactivity for cross-coupling reactions.	^53^
Josiphos SL-J009-1-G3-Palladacyclic complex	C-C	Heck Reaction Cross-Coupling Reactions	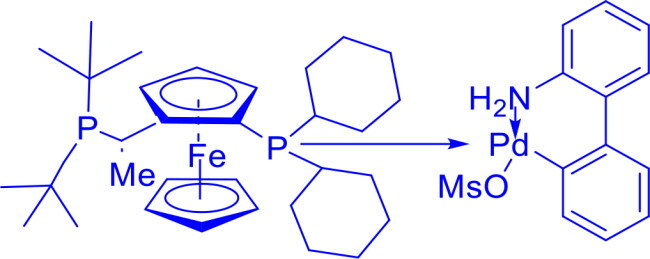	The Josiphos coxplex highly enantioselective and stable catalyst, ideal for asymmetric organic synthesis due to its chiral Josiphos ligand and palladacyclic structure.	^54^ ^,^ ^55^
2-(Dicyclohexylphosphino)-2′,4′,6′-triisopropyl-1,1′-biphenyl CPhos Pd G3	C-C	Suzuki Coupling, Sonogashira Coupling, Negishi Coupling	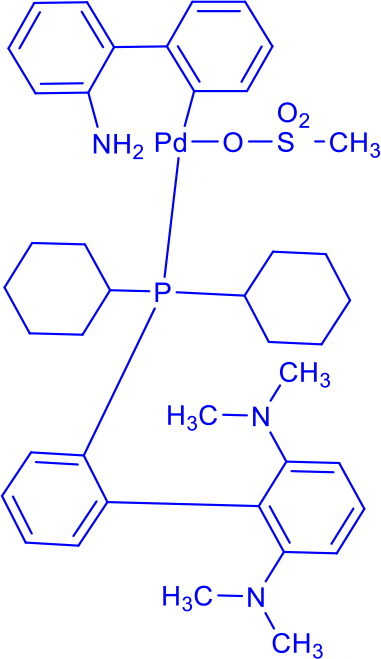	CPhos Pd G3 is a stable, highly reactive palladium catalyst that works well at low loadings for tough cross-coupling reactions, including sterically hindered and electron-poor substrates.	^56^
2,2′-bis(diphenylphosphino)-1,1′-binaphthyl BINAP-PdCl _2_ 2-Dicyclohexylphosphino-2′,6′-dimethoxybipheny	C-C C-N	Buchwald-Hartwig Cross Coupling Reaction, Heck Reaction, Hiyama Coupling, Negishi Coupling, Sonogashira Coupling, Stille Coupling, Suzuki-Miyaura Coupling	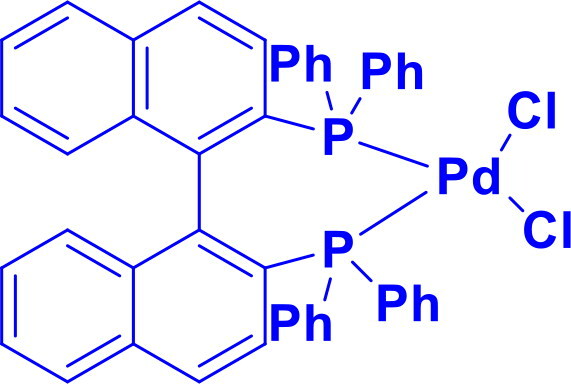	BINAP-PdCl _2_ is a highly versatile and enantioselective chiral catalyst, valued for its rigid, chiral BINAP ligand, which imparts exceptional stereocontrol in asymmetric transformations.	^57^
SPhos Pd G3	C-C C-N	Suzuki-Miyaura Coupling, Buchwald-Hartwig Coupling, Heck Reaction, Hiyama Coupling, Negishi Coupling, Sonogashira Coupling, Stille Coupling	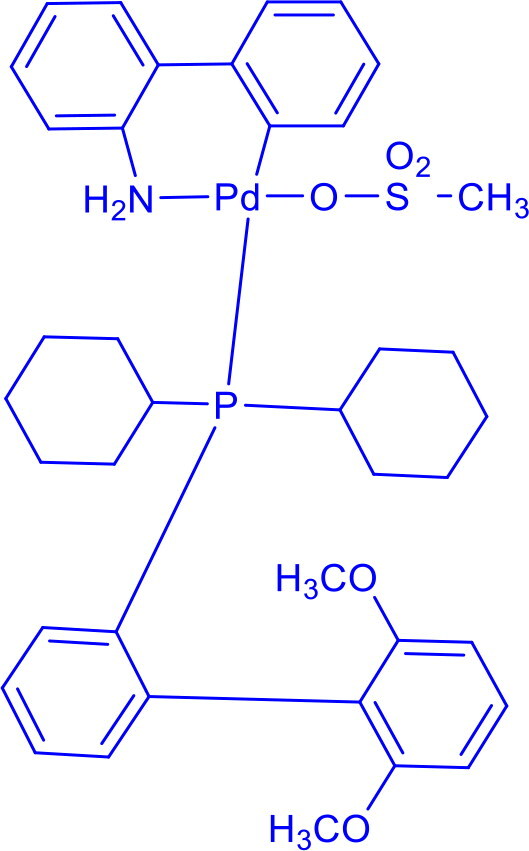	The SPhos ligand, a sterically electron-rich phosphine, stabilises the palladium centre, accelerates key catalytic steps, prevents Pd(0) aggregation, and ensures SPhos Pd G3′s stability under ambient conditions for easy handling and storage.	^58^
2-(Di-tert-butylphosphino)-3,6-dimethoxy-2′,4′,6′-triisopropyl-1,1′-biphenyl *tert* -BuBrettPhos-Pd-G3	C-C C-N	Suzuki-Miyaura Coupling, Buchwald-Hartwig Coupling, Heck Reaction, Hiyama Coupling, Negishi Coupling, Sonogashira Coupling, Stille Coupling	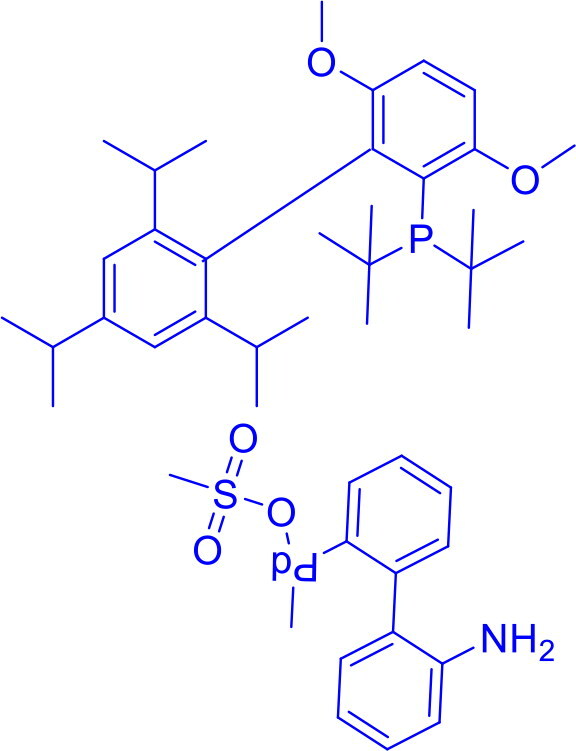	Features a sterically hindered, electron-rich t-BuBrettPhos ligand and Pd-G3 precatalyst design, enabling high reactivity, stability, rapid activation, exceptional performance with hindered substrates, broad functional group tolerance, and efficient operation at low loadings.	^59^ ^,^ ^60^
2-Dicyclohexylphosphino-2′,6′-diisopropoxybiphenyl RuPhos Pd G3	C-O C-N C-C	Buchwald-Hartwig amination, Suzuki-Miyaura coupling, Negishi coupling	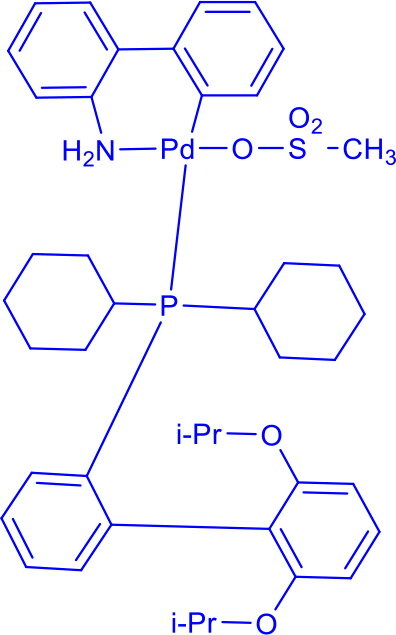	RuPhos Pd G3 has a bulky, electron-rich phosphine that stabilises the palladium centre and promotes efficient reductive elimination,	^61^
Dichloro[bis(2-(diphenylphosphino) phenyl)ether] palladium(II) Pd(DPEPhos)Cl₂,	C-C	Sonogashira coupling Suzuki Coupling	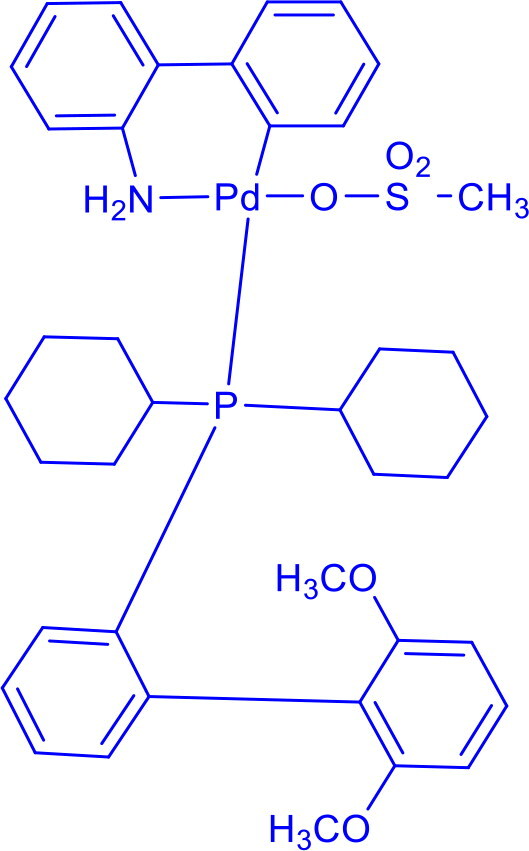	The ether oxygen in the ligand backbone can influence the electronic properties of the complex. It may also participate in weak interactions	^62^
N,N-bidentate diimine ligand Pd(quinox)Cl_2_	C-C C-N	Wacker reaction, Buchwald-Hartwig Reaction, Suzuki Coupling reaction, Hiyama coupling	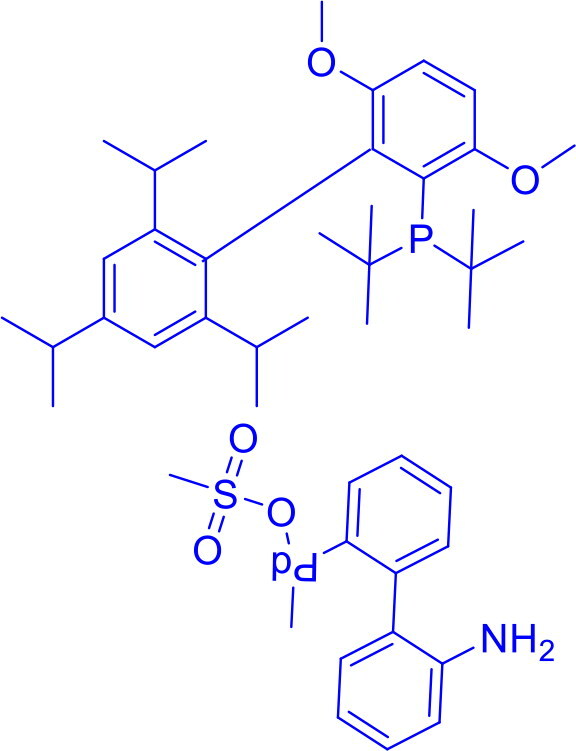	The quinoxaline ligand, being an electron-deficient heterocycle, slightly withdraws electron density from the palladium centre, making it more electrophilic.	^63^
Palladium-tetrasodium tetra(p-sulfonatophenyl)porphyrin Palladium(II)-Porphyrin Complexes	C-C	Suzuki–Miyaura cross-coupling Heck Reaction	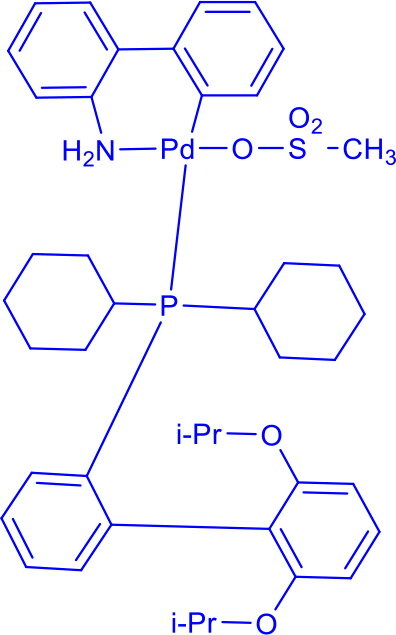	Operates at very low catalyst loadings and under air- and moisture-tolerant conditions, Water-soluble palladium(II) porphyrin complex due to four sulphonate groups on the phenyl rings	^63^

Over the years, a rampant rise in the prevalence of cancer has been witnessed, which in turn has served as a fodder for the organic growth of the palladium-based catalyst market in pursuit of accomplishing target-oriented synthesis of antitumor constructs^4^. This review article highlights the applications of palladium that have been used for the generation of epigenetic inhibitors as antitumor agents. Literature precedents reveal that epigenetic dysregulation is a well-identified feature of cancer, and epigenetic alterations are involved in various stages of tumour progression. In pursuit of reversing the epigenetic alterations, various classes of epigenetic inhibitors have been developed lately, and the outcomes have been optimistic enough to label such chemical classes as promising targeted therapies^14^^,^^15^. A glance at the literature on the epigenetic inhibitors reported in the last decade indicates that Pd-catalysed reactions are the linchpin of synthetic methodologies for the construction of complex therapeutic agents modulating multiple aspects of epigenetics. Their capacity to form diverse C-C and C-N bonds has provided unparalleled versatility and precision in the synthetic organic chemistry used by drug discovery teams. A careful analysis of the chemical routes to epigenetic inhibitors reveals that achieving high yields in Pd-catalysed transformations, especially for structurally complex substrates, requires a multifaceted optimisation strategy^16^^,^^17^. Prudent selection of ligands for the palladium catalyst, bases, and solvents, along with catalyst loading, defines the success of such endeavours in terms of efficient synthesis of the designed adducts. It is important to mention that the selection of the ligand (monodentate ligands, bulky ligands, bidentate ligands, and strong sigma donors) was found to be extremely critical in this context, as it controls the reactivity, selectivity, and stability of the palladium complex. The availability of a large number of ligands conferred a scope to the medicinal chemistry teams to pinpoint a suitable combination of palladium catalyst and ligand for enhancing the reactivity of the palladium complex. Base screening (inorganic bases and organic bases) again proved to be an important aspect in this regard, as it influenced the deprotonation steps. In addition to their ability to deprotonate, bases were selected based on their compatibility with other reagents as well as solubility in the chosen solvent. Solvent optimisation was also found to be one of the most challenging aspects of palladium-catalysed multistep synthetic routes, as the choice of the solvent, whether polar aprotic, less polar, or green alternatives, has an impact on catalyst stability and ligand solubility.

**Figure 2.**
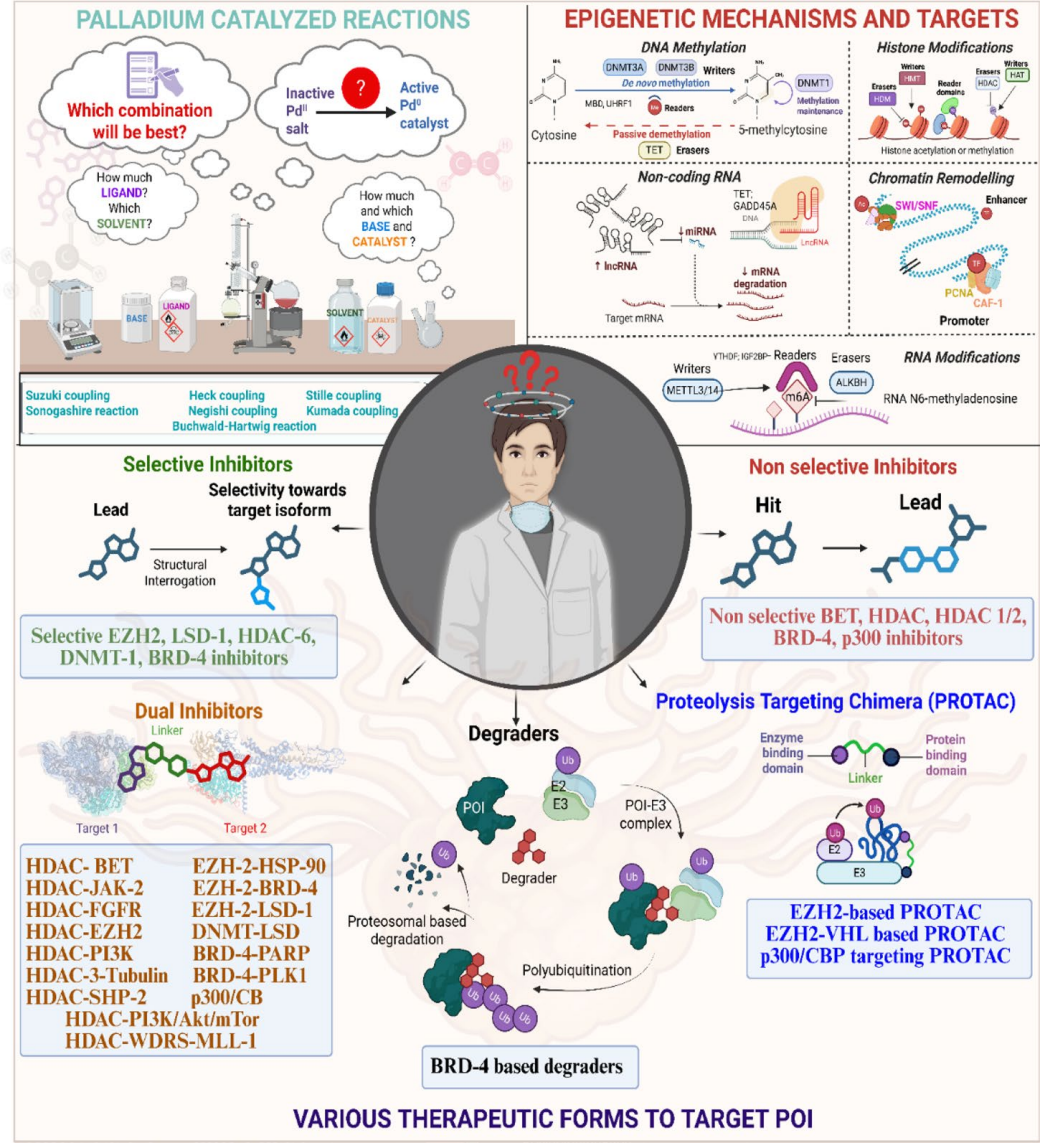
Palladium catalysis and small molecule epigenetic inhibitors: A hand in glove alliance (the figure was drawn by the authors using BioRender software).

This review systematically surveys and critically analyzes advances reported over the past five years (2020–2025) in the palladium-catalysed methodologies applied to epigenetic drug discovery, which highlights their broad applicability across heterocycles, functionalised arenes, and pharmaceutically relevant scaffolds used in cellular epigenetics programs (Figure 2). Distinct from earlier reviews that primarily catalogue cross-coupling reactions or focus on general medicinal chemistry aapplications, including recent perspectives on sustainable palladium catalysis^17^, the present manuscript uniquely integrates synthetic innovations with epigenetic target engagement and biological validation. By correlating structural, mechanistic, and procedural parameters such as catalyst-ligand selection, base effects, solvent systems, and reaction efficiency with downstream biological performance, this manuscript provides a translational framework that bridges synthetic chemistry and functional epigenetics. Importantly, we emphasise how systematic optimisation of palladium-catalysed processes has enabled reduced catalyst loadings, improved reaction sustainability, and late-stage diversification of epigenetically active scaffolds for the synthesis of selective/non-selective/dual-inhibitors, degraders, and PROTACS. In addition to the methodological advances, we comprehensively summarise robust drug-design strategies, structure-activity relationships, and *in vitro*/*in vivo* activity profiles of representative epigenetic inhibitors. Noteworthy to mention that, the drug discovery sleuths have left no stone unturned in attempting to maximise the efficiency of palladium catalysis by investing a substantial amount of effort to pinpoint the most efficient combination of palladium catalysts, ligands, bases, and solvents that can smoothly catalyse the key steps of the chemical routes to small molecule epigenetic inhibitors. Resultantly, the structural pool of epigenetic inhibitors is currently loaded with numerous preclinically active tractable small molecule epigenetic inhibitors that are knocking on the doors of clinical studies. Collectively, the insights compiled herein extend beyond a descriptive survey and offer a practical roadmap for academic and industrial researchers seeking to design efficient, scalable, and biologically impactful epigenetic therapeutics.

## Epigenetics targets and palladium catalysis for the construction of small molecule epigenetic inhibitors

In eukaryotic cells, the genome is intricately packaged into nucleosomes, comprising 146 base pairs of DNA wrapped around histone octamers, which further organise into higher-order chromatin structures^64^. Chromatin architecture dynamically regulates the expression of many genes, wherein compacted chromatin restricts the transcriptional access; however, relaxed configurations allow engagement of transcriptional machinery^65^. This regulation is principally mediated by epigenetic mechanisms that comprise DNA methylation, histone modifications, nucleosome remodelling, and non-coding RNA interactions, without any alteration of the underlying DNA sequence^66^^,^^67^. Since the introduction of the concept by Conrad Waddington in 1942, the definition of epigenetics has evolved to encompass mitotically and meiotically heritable changes in gene expression mediated by chromatin modifications^68^^,^^69^. Epigenetic regulation is orchestrated by a coordinated interplay of “writers,” which deposit chemical markers on histones or DNA, “readers,” which are methyl-CpG-binding domain proteins that recognise these modifications, and “erasers,” generally chromatin-modifying enzymes that help to remove them^70–72^. Together, these factors establish and maintain cell-specific gene expression programs critical for normal development, tissue homeostasis, and regulation of DNA-based functions like transcription, DNA repair, and replication^73^. Disruption of epigenetic landscapes is a near-universal feature of malignancy, contributing to the dysregulation of pathways governing proliferation, apoptosis, genomic stability, and metastasis^74^. In cancer, the epigenome is profoundly perturbed, characterised by widespread DNA hypomethylation, promoter-specific hypermethylation of tumour suppressor genes, aberrant histone modification profiles, and dysregulation of chromatin-remodelling enzymes^75^. These alterations not only cooperate with genetic mutations to drive oncogenesis but can also independently initiate and sustain malignant phenotypes^76^. Further, environmental factors, such as ultraviolet radiation, can also exacerbate epigenetic dysregulation, contributing to tumour initiation through persistent alterations in gene regulatory networks^77^. Epigenetics also contributes to cellular heterogeneity within tumours, complicating therapeutic responses and enabling cancer cells to adapt to adverse conditions^78^. As research advances, epigenetics is increasingly recognised not only as a mechanism underpinning cancer biology but also as a key to improving diagnostics, prognosis, and personalised therapies^79^. Various key epigenetic targets have been recognised in the past few years, mediating the methylation, acetylation, and other components of epigenetics in cancerous cells, which are mentioned below:

**DNA and RNA Methylation Dynamics:** DNA methylation involves the covalent addition of a methyl group to the C5 position of cytosine residues, predominantly within CpG dinucleotides, catalysed by DNA methyltransferases (DNMTs)^80^. Cancer genomes exhibit distinct patterns of global DNA hypomethylation coupled with site-specific promoter hypermethylation, reflecting two mechanistically independent but coexisting epigenetic alterations^81^^,^^82^. DNA methylation, primarily at CpG islands, silences gene expression through the actions of DNMT1, DNMT3a, and DNMT3b, while TET enzymes mediate active demethylation to maintain transcriptional plasticity^71^^,^^83^. In parallel, N6-methyladenosine (m6A) modifications on mRNA, dynamically regulated by “writers,” “erasers,” and “readers,” fine-tune RNA fate, impacting transcription, RNA splicing, stability, and translation^84^. Disruptions in DNA and RNA methylation homeostasis are closely linked to development, lineage specification, and oncogenic transformation, highlighting their significance as promising targets for therapeutic intervention in cancer^85^. DNMTs have emerged as viable targets for oncogenic therapy; therefore, azacitidine and decitabine were FDA-approved DNMT inhibitors^86^. Following this, ivosidenib and enasidenib have been used to target IDH1/IDH2 mutations and the TET enzyme in order to modulate various components of DNA methylation and demethylation in oncogenic setups^87^.

**Epigenetic Modulation of Chromatin and Gene Expression:** Histone post-translational modifications (PTMs) - including acetylation, methylation, ubiquitylation, phosphorylation, and others- dynamically regulate chromatin structure and transcriptional activity^88^. Histone acetylation, mediated by histone acetyltransferases (HATs) and reversed by Histone deacetylases (HDACs), neutralises lysine charges to promote transcription, while histone methylation has context-dependent outcomes: methylation at H3K4/36/79 activates transcription, whereas H3K9/27 and H4K20 methylation enforces repression^89^^,^^90^. Specific histone-modifying enzymes such as EZH2 and SET7/9 establish and maintain these marks, orchestrating cellular identity and influencing disease progression^91^^,^^92^. Various key targets of histone post-translational modifications are (i) Histone Methyltransferases (HMTs) mediate histone methylation linked to gene silencing, and it is targeted by EZH2 inhibitor tazemetostat^93^. (ii) Histone Demethylases (HDMs) impact gene activation and repression by removing the methyl group, and it is mainly mediated by lysine-specific demethylase-1 (LSD-1). Various LSD inhibitors are under multiple phases of clinical trials for targeting HDMs in cancer^94^. (iii) HDACs promote chromatin condensation and transcriptional repression, and their counterpart HATs are studied for their active role in gene activation. Hence, various inhibitors like vorinostat, romidepsin, and others were FDA-approved for various oncogenic malignancies^95^^,^^96^. (iv) BET Proteins (Bromodomains) recognise acetylated proteins and regulate oncogene transcription. BET inhibitors like JQ1 and birabresib have been studied to inhibit BET in cancer^97^. (v) Targeting Polycomb Repressive Complexes (PRCs) and EZH2/PRC2 complex through tazemetostat has exhibited prominent anticancer efficacy by inhibiting the H3K27 methylation^98^.

**Non-Coding RNAs as Epigenetic Regulators:** Non-coding RNAs (ncRNAs), which represent over 70% of the human genome, functionally regulate gene expression through diverse mechanisms^99^. MicroRNAs (miRNAs) post-transcriptionally silence genes by targeting 3′ UTRs, influencing ∼60% of coding genes, and their dysregulation- often via epigenetic alterations at CpG islands- contributes to oncogenesis^100^. Long non-coding RNAs (lncRNAs) and circular RNAs (circRNAs) act as scaffolds, enhancers, and miRNA sponges, with emerging evidence showing their capacity to encode regulatory micro peptides, thus adding additional layers to the complexity of gene regulatory networks in health and disease^101^. As ncRNAs modulate gene expression post-transcriptionally, various ncRNA-based therapies are under exploration preclinically.

**Figure 3.**
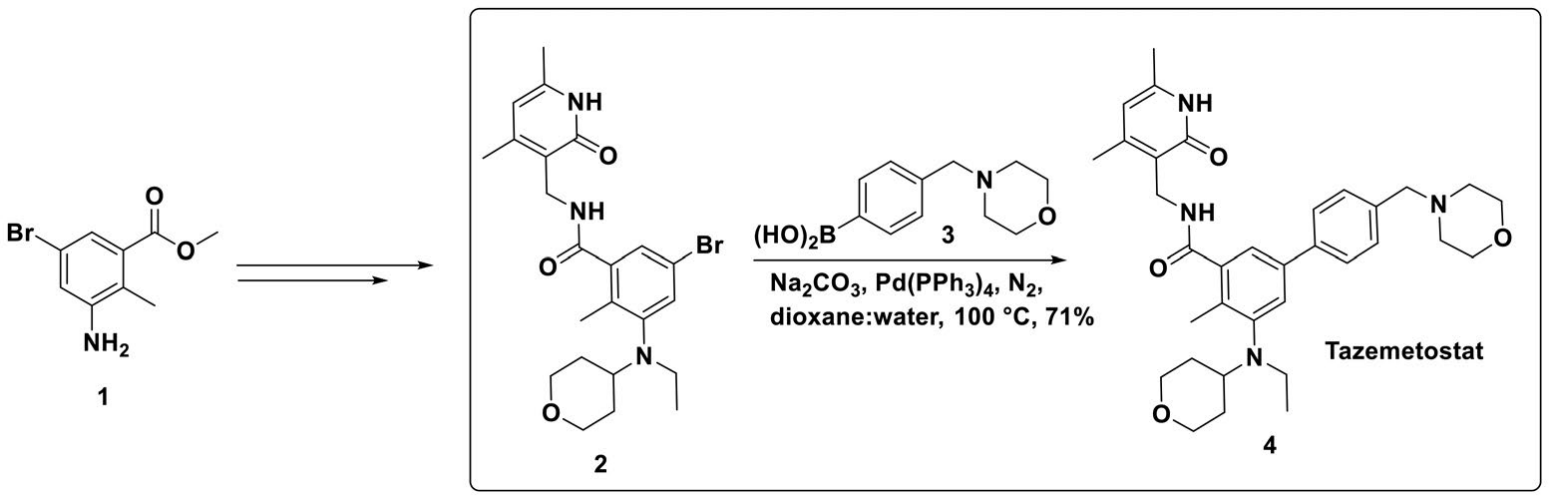
Synthetic Route of FDA-Approved EZH2 Inhibitor (the figure was drawn by the authors using chemdraw software).

Palladium-catalysed reactions, particularly cross-coupling reactions, have become indispensable in the synthesis of structurally complex small molecules. These reactions offer high chemo-selectivity, functional group tolerance, and scalability- critical features in drug development pipelines. Several FDA-approved agents targeting epigenetic regulators such as HDACs and methyltransferases utilise Pd-catalysed steps for the formation of key C-C or C-N bonds. One prominent example is Tazemetostat, the first FDA-approved EZH2 inhibitor, developed by Epizyme for the treatment of metastatic or locally advanced epithelioid sarcoma. It functions by blocking histone H3K27 hyper-trimethylation, thereby preventing tumour dedifferentiation^102^. The synthesis of tazemetostat involves a convergent sequence, culminating in a palladium-catalysed C-C bond-forming reaction. Initially, methyl 3-amino-5-bromo-2-methylbenzoate undergoes sequential reductive amination with tetrahydro-4H-pyran-4-one and subsequently with acetaldehyde to yield a key benzylic amine intermediate. Saponification of the ester followed by amide bond formation with an amine partner, mediated by PyBOP, furnishes the penultimate intermediate. The pivotal transformation involves a Suzuki-Miyaura cross-coupling reaction, where the bromoarene was coupled with a boronic acid derivative in the presence of tetrakis(triphenylphosphine)palladium(0)\[Pd(PPh_3_)_4_], sodium carbonate, and a dioxane-water solvent system at 100 °C. This palladium-catalysed step efficiently installs the aryl moiety, delivering tazemetostat in 71% yield, underscoring the critical role of Pd catalysis in the construction of the bioactive framework, as shown in Figure 3^103^.

**Figure 4.**
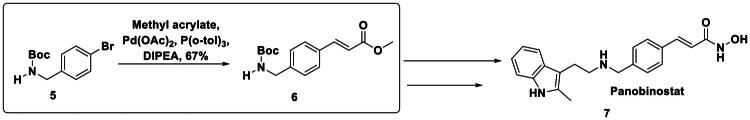
Synthetic Route of FDA-Approved HDAC Inhibitor (the figure was drawn by the authors using chemdraw software).

Furthermore, Panobinostat is a potent, non-selective HDAC inhibitor approved for the treatment of multiple myeloma. It targets class I, II, and IV HDAC enzymes, promoting hyperacetylation of histones and non-histone proteins, leading to transcriptional activation of tumour suppressor genes, cell cycle arrest, and apoptosis^104^. Structurally, panobinostat features a hydroxamic acid moiety critical for zinc ion chelation at the HDAC active site. Its broad-spectrum HDAC inhibition disrupts cancer cell survival pathways, making it a powerful agent in epigenetic cancer therapy. Figure 3 illustrates the synthesis of Panobinostat via a convergent strategy involving key palladium-catalysed and reductive amination steps. Initially, a Boc-protected 4-bromoaniline undergoes a Pd(OAc)_2_-catalysed Heck coupling with methyl acrylate in the presence of tri(o-tolyl)phosphine and DIEA, forming an (E)-methyl cinnamate derivative. Notably, with a low molar loading of Pd catalyst (1% mol), a high yield of cinnamate derivative (67%) was obtained. Subsequent nucleophilic substitution of the ester group, deprotection of the Boc protecting group using trifluoroacetic acid, and reductive amination with an indole-3-carboxaldehyde using sodium triacetoxyborohydride lead to Panobinostat, typically isolated as its lactate salt for enhanced bioavailability. Hence, the Pd-catalysed coupling reaction laid a pivotal foundation in the synthesis of Panobinostat, enabling the efficient construction of key aryl-olefin intermediate essential for subsequent pharmacophore construction and HDAC inhibition (Figure 4)^104^.

**Figure 5.**
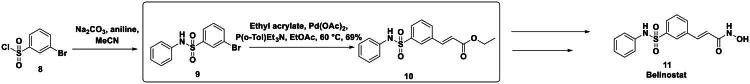
Synthetic Route of FDA-Approved HDAC Inhibitor (the figure was drawn by the authors using chemdraw software).

Belinostat (PXD101), a pan HDAC inhibitor, got FDA approval in 2014 for the treatment of T-cell lymphoma. The synthetic route for Belinostat begins with the formation of a sulphonamide intermediate via nucleophilic substitution between 4-bromobenzenesulfonyl chloride and aniline in the presence of a base (DMAO) in toluene. The sulphonamide undergoes a Pd(0)-catalysed Heck coupling reaction with ethyl acrylate using triethylamine (29.4 mol), tri(o-tolyl)phosphine (0.4 mol), and palladium (II) acetate (0.2 mol), forming the trans-alkene product with high regio- and stereoselectivity and overall yield of 69%. Next, hydrolysis, acidification, and condensation with hydroxylamine (NH_2_OH) yield the final hydroxamic acid product, Belinostat. The key steps involve the efficient construction of the carbon-carbon double bond via Heck coupling and the introduction of the hydroxamic acid functionality critical for HDAC inhibition, as represented in Figure 5^105^.

The applications of palladium-catalysed cross-coupling reactions are backed by a few precedents in the context of FDA-approved small molecule epigenetic inhibitors; however, their utility is clearly reflected via a glance at the preclinical studies (medicinal chemistry campaigns). Notably, the structural pool of small molecules addressing the epigenetic targets (preliminary and preclinical studies) has been expanded via C-C/C-N bond formation strategies on numerous instances. Several convincing examples (preclinical investigations - drug discovery endeavours) have been included in this section to demonstrate the broad applicability of Pd-catalysed methodologies in cellular epigenetics. Gladly, the armoury of palladium catalysts, at present, is decently sized, enabling the chemist to perform exhaustive optimizations of the cross-coupling reactions required to generate the target structural templates via selective C–C and C-N bond-forming reactions. The list of established as well as emerging palladium catalysts, along with key notions regarding the applicability and characteristics of the palladium catalysts, is covered in Table 1. Noteworthy to mention that palladium catalysis not only accelerated the synthesis of epigenetic modulators but also enhanced their synthetic tractability, scalability, and diversification potential, making it a cornerstone in the medicinal chemistry of epigenetic drug development^16^^,^^17^. The drug discovery studies presented in this section have been delved into various subsections on the basis of the palladium catalyst used either for the efficient construction of the intermediates or the generation of target compounds.

### Palladium (II) chloride (PdCl_2_) and related catalysts assisted construction of epigenetic inhibitors

Palladium (II) chloride (PdCl_2_) is a highly versatile catalyst in organic synthesis, known primarily for its widespread application in cross-coupling reactions, including Heck, Suzuki, and Stille couplings^18–21^. Notably, PdCl_2_ also acts as a key precursor for synthesising other catalytically active palladium species, such as Pd nanoparticles and Pd(0) complexes^22^. Also, PdCl_2_(PPh_3_)_2_ featuring triphenylphosphine ligand to stabilise the palladium centre is frequently employed in Negishi, Sonogashira, and Heck couplings^23^. Noteworthy to mention that some organopalladium complex featuring the dppf ligand serves as highly efficient catalysts for Buchwald-Hartwig amination (C–N bond formation) and Suzuki-Miyaura coupling (C–C bond formation)^19^^,^^106–110^. The dppf ligand stabilises the palladium centre through a combination of steric and electronic modulation, thereby promoting the catalytic cycle^111^^,^^112^. Numerous studies have been covered in this section that underscore the contribution of the aforementioned catalysts in accomplishing the epigenetic targeting chemotypes.

**Figure 6.**
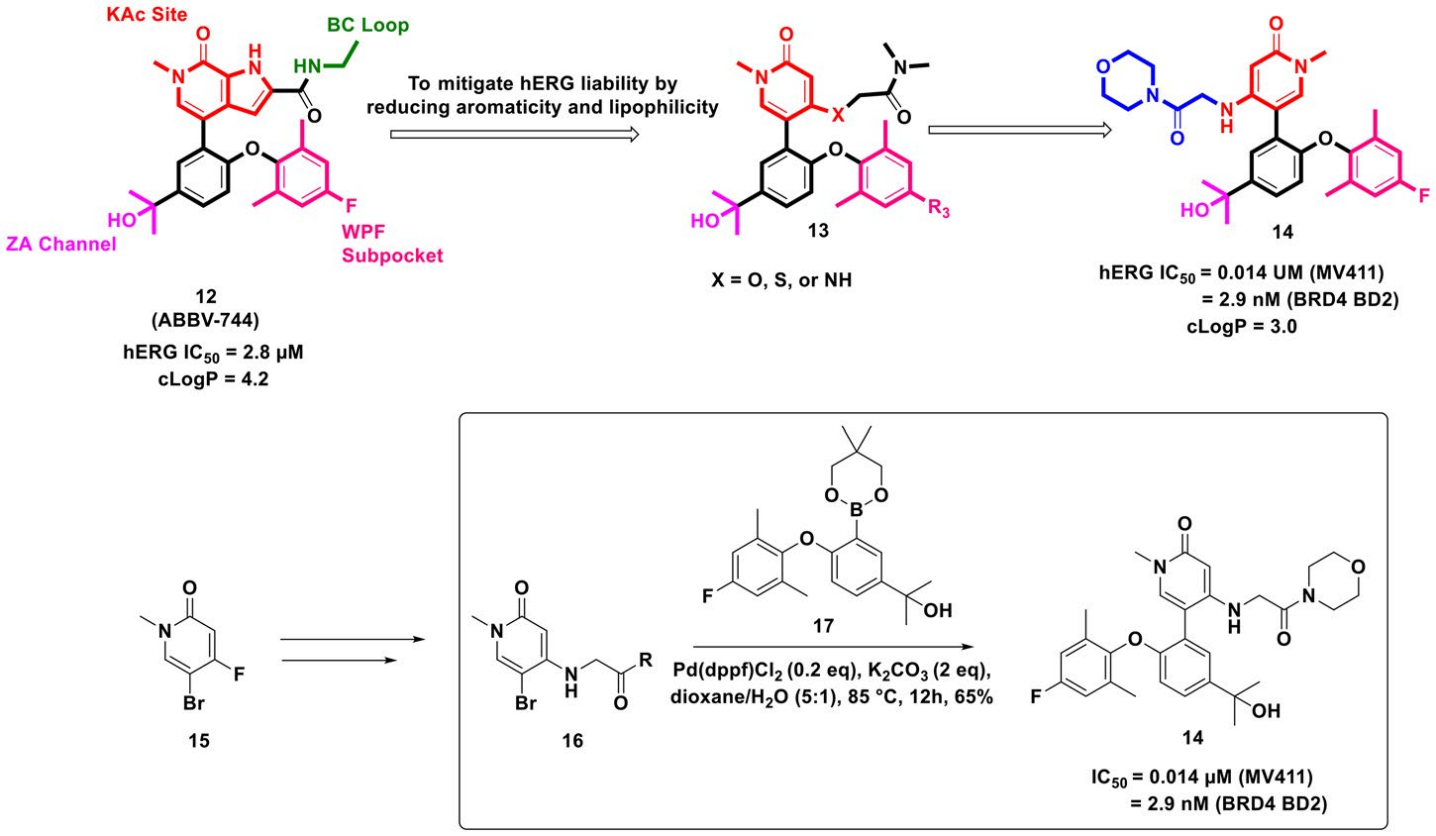
Design Strategy & Synthetic Route of Phenoxyaryl Pyridones Based Bromodomain & Extra Terminal Domain Inhibitors (the figure was drawn by the authors using chemdraw software).

The emergence of BET Proteins as a therapeutic target for the design of antitumor scaffolds is attributed to precedential reports ascertaining the dysregulation of BET proteins in cancer. The quest to capitalise on the strategy of modulating the BET proteins via small molecule structural templates initiated with the campaigns on pan-BET inhibitors; however, the focus of the ongoing endeavours has transposed towards Bromodomain-selective BET inhibitors in pursuit of attaining BET modulatory chemical architectures with improved safety profiles. With this background, Jiang *et al.* embarked on a medicinal chemistry endeavour and leveraged the catalytic potential of Pd(dppf)Cl_2_ to synthesise a logically designed selective BET inhibitor (**14**) (as shown in Figure 6). Notably, the BET inhibitor was furnished via a multistep synthetic route that involved a reaction sequence comprising of classical organic chemistry methodologies *viz.* nucleophilic substitution, lithium hydroxide assisted ester hydrolysis, HATU mediated amidation and the organopalladium catalysed C-C bond formation (Suzuki cross-coupling reaction). Important to mention that Pd(dppf)Cl_2_ was employed in the final step of the synthetic protocol to afford the C-C bond formation between 5-Bromo-1-methyl-4-((2-morpholino-2-oxoethyl)amino)pyridin-2(1H)-one and 2-(3-(5,5-dimethyl-1,3,2-dioxaborinan-2-yl)-4-(4-fluoro-2,6-dimethylphenoxy)phenyl) propan-2-ol. The reaction was conducted in dioxane/water at 85 °C for 12h and employed potassium carbonate as the base. The combination of Pd(dppf)Cl_2_ and K_2_CO_3_ is backed by numerous instances to smoothly catalyse the Suzuki coupling, as dppf provides a stable and electron-rich Pd complex to enhance the oxidative addition to aryl halides, while the boronate species required for transmetalation is generated by K_2_CO_3_. Delightfully, the aforementioned reaction conditions and the application of Pd(dppf)Cl_2_ as a catalyst led the research group to attain compound (**14**) in an overall 65% yield, which was considered more than satisfactory as this was the final step of the synthetic route. Given the attainment of the compound in satisfactory yields, the research group was able to conduct an exhaustive exploration (*in vitro* and *vivo*) of the compound (**14**) to profile it as a prospective therapeutic for acute myeloid leukaemia. Encouragingly, compound (**14**) demonstrated magnificent selective BRD4 BD2 inhibitory effects that led to striking cell growth inhibition of acute myeloid leukaemia (AML) cell lines. The compound was found to be an apoptosis inducer as it caused cell cycle arrest at G_0_/G_1_ arrest and was also endowed with striking *in vivo* antitumor potential in a study conducted in the MV411 mouse xenograft model^113^.

**Figure 7.**
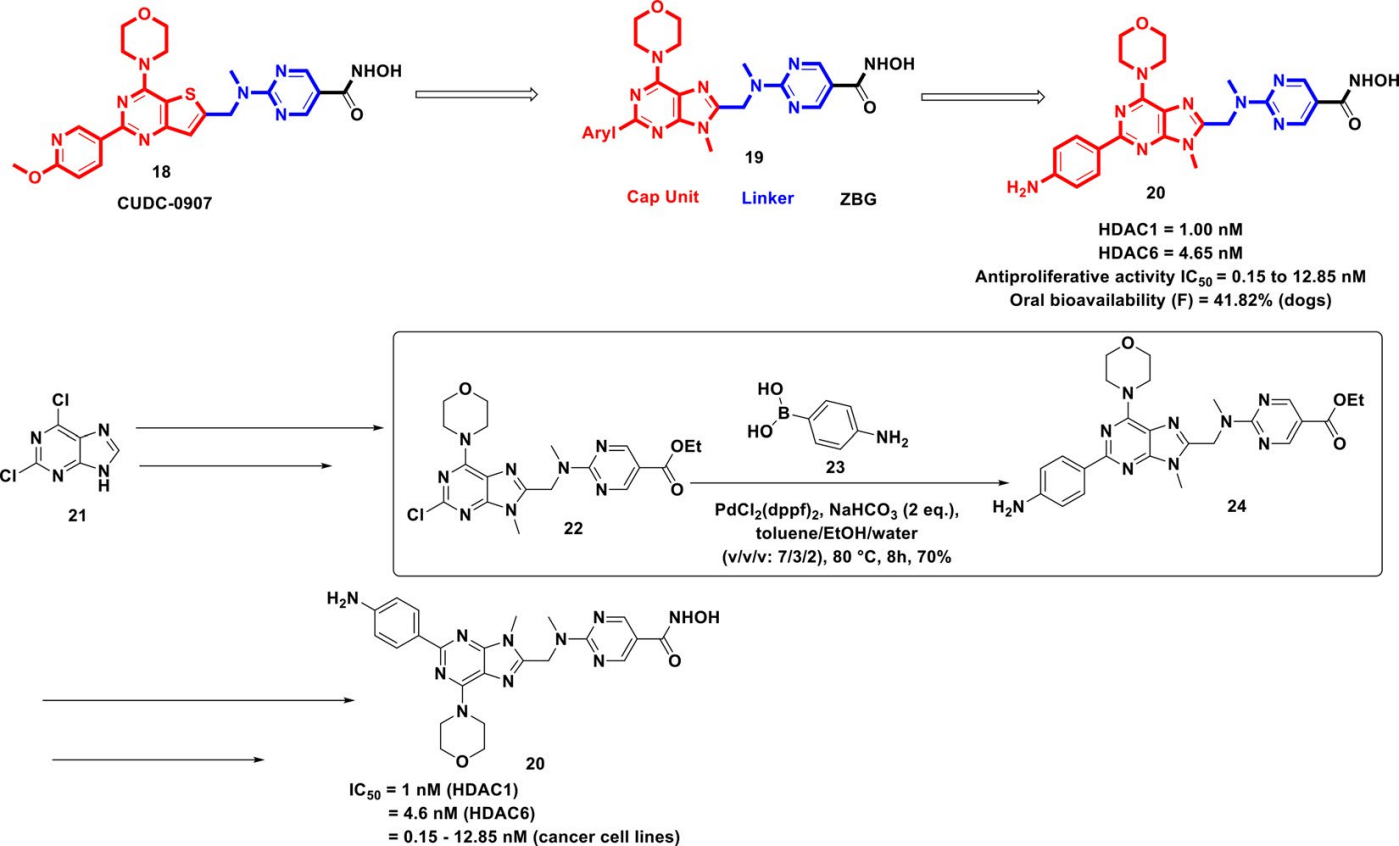
Design Strategy & Synthetic Route of Purine-Based Hydroxamic Acid Inhibitors (the figure was drawn by the authors using chemdraw software).

Chen *et al.* attempted to assemble an HDAC inhibitor via structural engineering of a well-established modular HDAC inhibitory pharmacophore comprising three structural components, *viz.,* surface recognition group, a linker, and a zinc binding motif. The research group planned to install a morpholinopurine scaffold as a surface recognition part with in the template, however, the plan was not just confined towards the aforementioned installation and assessment of the impact of diverse N-alkyl/cycloalkyl substitutions as well as the placement of C-C bond assisted aryl/heteroaryl rings (purine core) on the enzymatic and cellular activity was also the aim of the study (as depicted in Figure 7). Notably, to accomplish the latter, the research group demonstrated their reliance on organopalladium catalysis to afford the stitching of diverse aryl/heteroaryl rings on the purine scaffold. These attempts culminated in a potent class I and class IIb HDAC inhibitor (**20**) endowed with a good pharmaceutical profile and striking *in vitro* antitumor activity. Also, the compound demonstrated magnificent *in vivo* antitumor activity in HCT116 xenograft models (tumour growth inhibition = 65.6%, i.v. treatment with 10 mg/kg), MV4-11 xenograft models (tumour growth inhibition = 68.2%, i.v. treatment with 10 mg/kg), and MM1S xenograft models (tumour growth inhibition of 75.1%). Important to mention that the route to the synthesis of HDAC inhibitor (**20**) involved a synthetic protocol comprising multiple steps, *viz*. nucleophilic substitution on the purine core to install the morpholine ring, followed by alkylation, formylation to introduce an aldehyde group using DMF and n-BuLi, reductive amination with methyl amine, and appendage with 2-chloropyrimidine-5-carboxylate using DIPEA. The aforementioned reaction sequence led to the conversion of 2,6-dichloropurine to ethyl 2-(((2-Chloro-9-methyl-6-morpholino-9H-purin-8-yl)-methyl) (methyl)amino) pyrimidine-5-carboxylate (**21**), which was further subjected to PdCl_2_(dppf)_2_ mediated Suzuki cross-coupling reaction with (4-aminophenyl) boronic acid (**22**). Notably, NaHCO_3_ was used as the base, and the solvent used for this C-C bond-forming reaction was toluene/EtOH/water (v/v/v, 7/3/2). The reaction was performed at 80 °C for 8h, which yielded the desired adduct (**24**), which on treatment with NH_2_OH produced the target compound (**20**). Notably, the preceding example employed pd(dppf)Cl_2_ as the palladium catalyst. Despite using the overcrowdedness in terms of ligands, PdCl_2_(dppf)_2_ smoothly catalysed the Suzuki coupling reaction and produced the Suzuki coupled adduct (**24**) in good yields, thereby enabling the author to proceed ahead towards the accomplishment of the target compound. Thus, the palladium catalysis played an instrumental role in the bulk-scale synthesis of the target compound, thereby enabling exhaustive *in vivo* evaluations in three xenograft models. Also, the palladium catalysis led the authors to install diversely substituted phenyl groups on the purine core, culminating in the generation of several intermediates, which turned out to be the access point to the most potent compound^114^.

**Figure 8.**
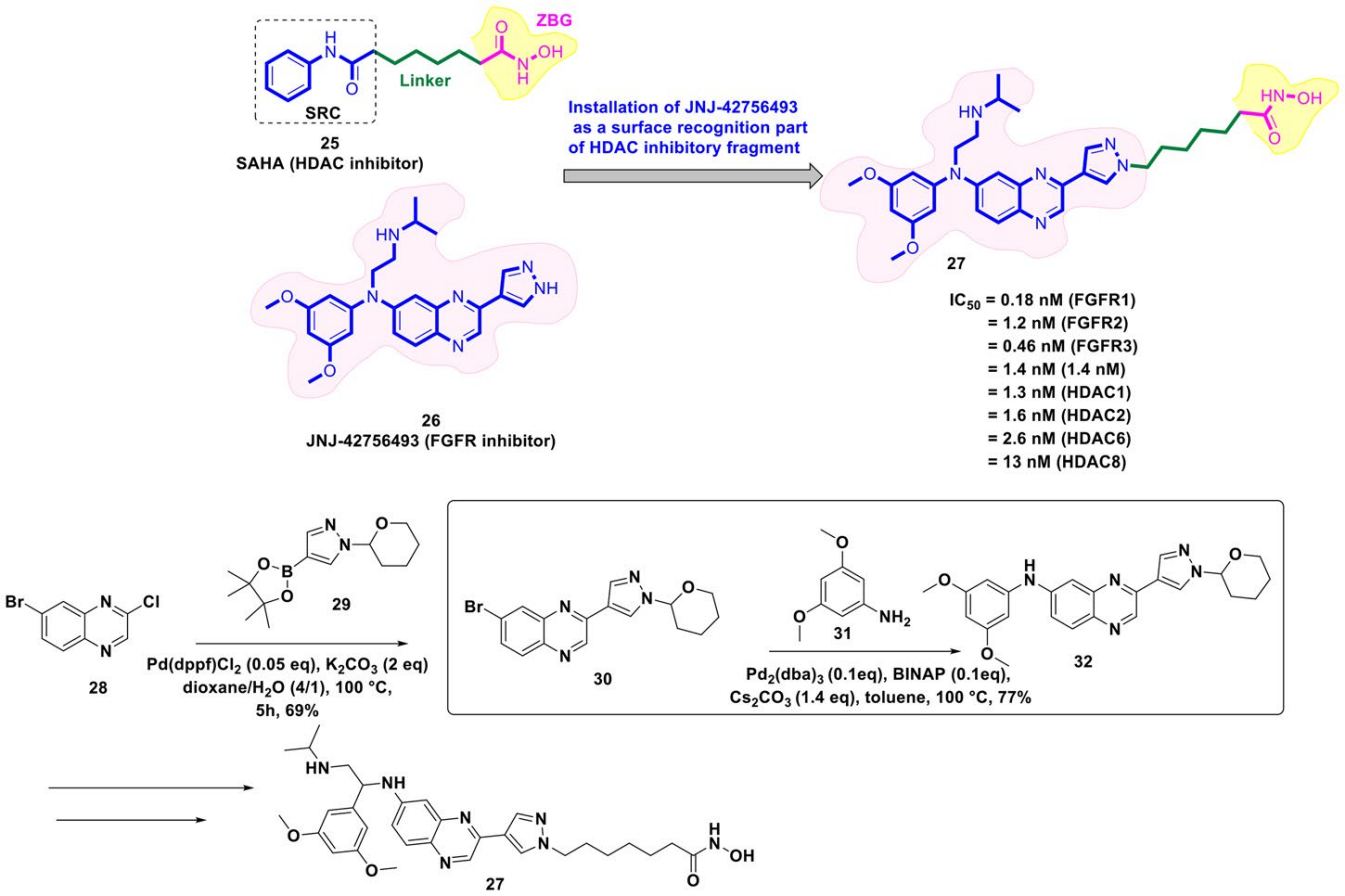
Design Strategy & Synthetic Route of Dual FGFR and HDAC Inhibitors (the figure was drawn by the authors using chemdraw software).

The strategy of dual inhibition has emerged as a validated approach to attain synergistic anti-tumour efficacy and counter the issue of drug resistance in cancer. Several recent disclosures ascertain that the STAT signal activation after HDAC inhibitor treatment enables the cancer cells to develop resistance towards HDAC inhibitors in solid tumours. To overcome the aforementioned issue, Wan *et al.* developed a compendium of logically constructed dual FGFR/HDAC inhibitors (design strategy shown in Figure 8) to capitalise on the revelations regarding the ability of FGFR inhibitors to downregulate pSTAT3 level. The generated scaffolds were subjected to a series of *in vitro* antitumor assays as well as *in vivo* antitumor evaluation. Amongst all the scaffolds, a dual FGFR1–4 and HDAC1/2/6/8 inhibitor (**27**) demonstrated striking *in vitro* cell growth inhibitory effects coupled with a remarkable *in vivo* antitumor profile (tumour growth inhibition in HCT116 and SNU-16 xenograft models) as well as an impressive pharmacokinetic profile. Also, compound (**27**) elicited the potential to overcome the resistance to HDAC inhibitors as it could downregulate the expression of p-STAT3. Noteworthy to mention that the synthetic route to (**27**) heavily relied on palladium catalysis, as the first two steps of the synthetic route designed for the generation of (**32**) (a key intermediate required for the synthesis of the target compound **27**) were catalysed by Pd(dppf)Cl_2_ and Pd_2_(dba)_3_. While Pd(dppf)Cl_2_ was used for the construction of the C-C bond through Suzuki cross-coupling reaction, Pd_2_(dba)_3_ was employed for the assemblage of the C-N bond (Buchwald-Hartwig amination). As shown in Figure 7, the starting material, 7-bromo-2-chloroquinoxaline, was treated with 1-[tetrahydro-2(H)-pyran-2-yl]-4-(4,4,5,5-tetramethyl-1,3,2-dioxaborolan-2-yl)-1H-pyrazole to generate the intermediate (**30**). Pd(dppf)Cl_2_ was used as the organopalladium catalyst, and potassium carbonate was used as the base for this step. The reaction was carried out in dioxane: H_2_O (4:1) at 100 °C for 5h, and (**30**) was obtained in acceptable yields (62%). Fourth, the successful synthesis of adduct (**30**), Pd_2_(dba)_3_ catalysis was used to transform the intermediate (**30**) to (**32**). The aforementioned Buchwald-Hartwig amination reaction also employed the application of BINAP (2,2’bis(dipheylphosphino)-1,1’binaaphthyl), which is usually employed in asymmetric Buchwald-Hartwig amination to attain a chiral amine product; however, in this case, a chiral amine was not the target intermediate. Thus, BINAP must have been chosen for its ability to form a highly stable palladium complex to enable smooth reaction between the halide and the amine. Caesium carbonate was used as the base, and the reaction was carried out in toluene at 100 °C overnight, yielding the intermediate (**32**) in good yields (72%). The intermediate (**32**) was further utilised for a multi-step reaction sequence to furnish the desired dual FGFR-HDAC inhibitor (**27**)^115^.

**Figure 9.**
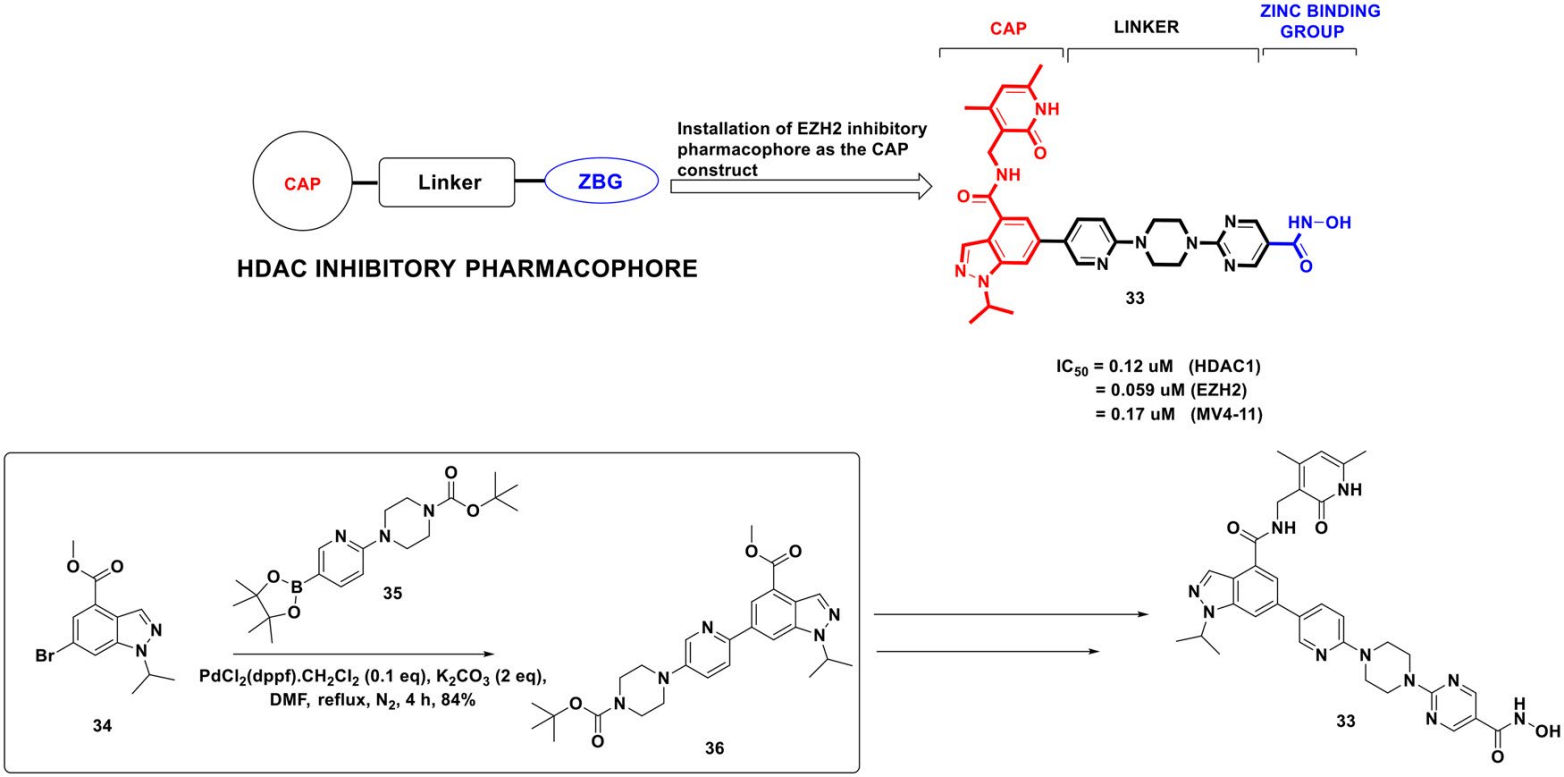
Design Strategy & Synthetic Route of Dual EZH2 & HDAC Inhibitor (the figure was drawn by the authors using chemdraw software).

The strategy to extract amplified antitumor effects in hematological malignancies via dual inhibition of HDAC and EZH2 is presently being given significant consideration by the medicinal chemist fraternity. The quest to supplement the library of dual EZH2-HDAC inhibitors as prospective antileukemic agents prompted Lu *et al.* to design chemical architectures that are based on structural commonalities of SAHA (HDAC inhibitor) and GSK126 (EZH2 inhibitor) as shown in Figure 9. The results of exhaustive biological evaluation aimed at elucidating the mechanistic underpinnings of designed structural templates led the research group to pinpoint an optimised scaffold (**33**) that was furnished via a synthetic pathway comprising of multiple steps. Important to mention that the second step of the synthetic route to scaffold (**33**) involved the appendage of methyl 6-bromo-1-isopropyl-1H-indazole-4-carboxylate (**34**) with *tert*-butyl 4-(5-(4,4,5,5-tetramethyl-1,3,2-dioxaborolan-2-yl)-pyridin-2-yl)piperazine-1-carboxylate (**35**). The reaction was carried out in DMF at 80 °C for 4h under the protection of nitrogen and was catalysed by [1,1′ Bis(diphenylphosphine)ferrocene]palladium(II)dichloride dichloromethane complex. The aforementioned synthetic protocol produced the adduct (**36**) in high yield (84%), which was further leveraged for the generation of scaffold (**33**) via a series of reactions. Scaffold (**33**) mediated anti-leukemic effects against MV4-11 cells 11 (IC_50_ = 0.17 *μ*M) via balanced dual inhibition of HDAC1 (IC_50_ = 0.12 *μ*M) and EZH2 (IC_50_ = 0.059 *μ*M). The remarkable antitumor potential of (**33**) was not just confined to *in vitro* studies, but a striking *in vivo* tumour suppression effect was also demonstrated by (**33**). Noteworthy to mention that compound (**33**) emerged to be the best of the series, which comprised 28 entries (designed dual HDAC-EZH2 inhibitors). The feasibility of choosing the most tractable scaffold out of numerous logically constructed chemical architectures in this study can be attributed to organopalladium catalysis to some extent, as the high-yielding initial step of the multistep chemical route served as a foundation for the late-stage structural diversification. Notably, structural diversification is highly imperative in medicinal chemistry to establish the structure-activity relationship that, in turn, renders enhanced probability to arrive at a pharmacokinetically and pharmacodynamically optimised scaffold^116^.

**Figure 10.**
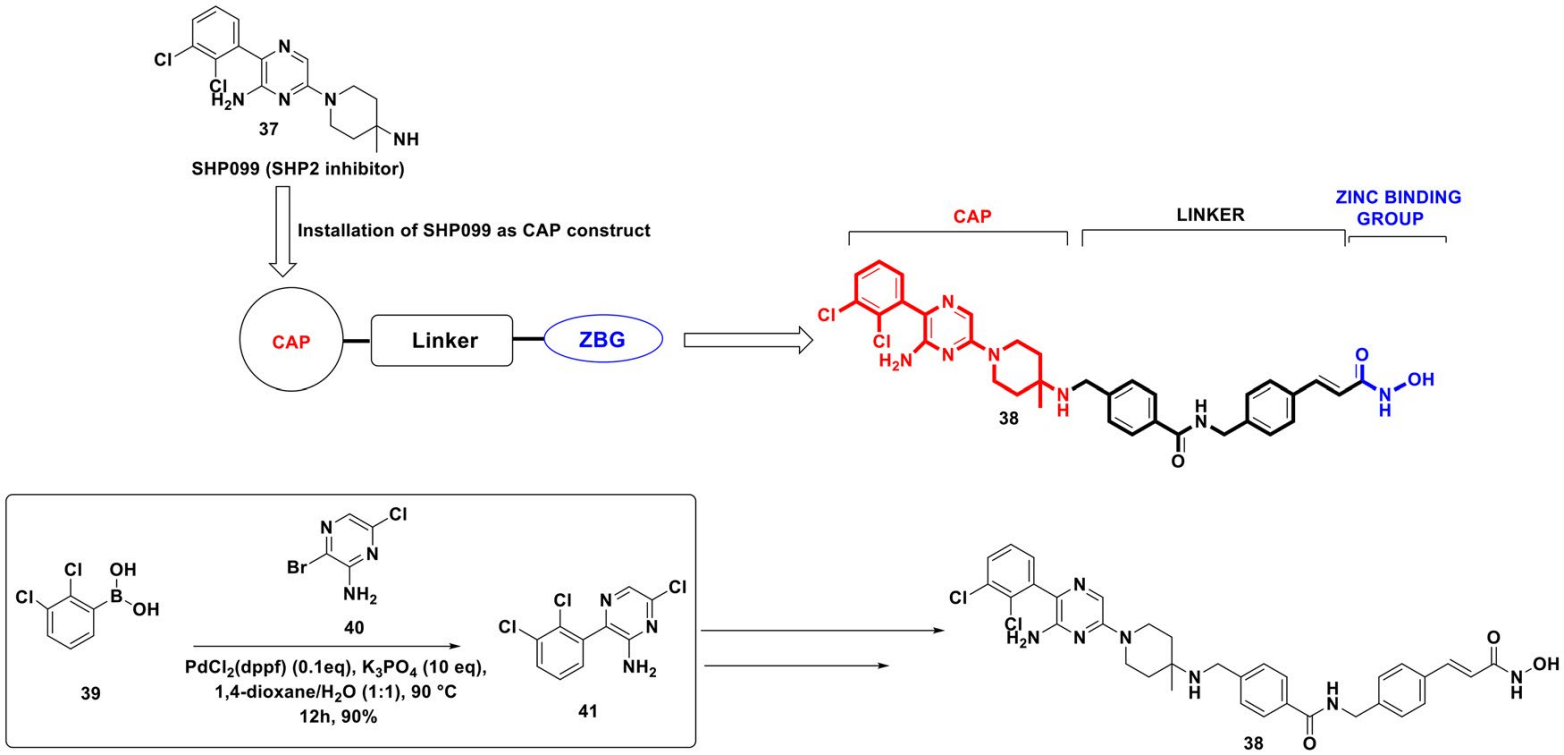
Design Strategy & Synthetic Route of Dual SHP2 & HDAC Inhibitors (the figure was drawn by the authors using chemdraw software).

Liu *et al.* employed the applications of the Suzuki cross-coupling reaction to generate a balanced dual modulator of Src homology-2 domain-containing phosphatase 2 (SHP2) and HDAC, as depicted in Figure 10. This pursuit to furnish a dual SHP2-HDAC inhibitor was spurred by preliminary observation of their research group, which ascertained that a cocktail of SHP099 (SHP2 inhibitor) and SAHA (HDAC inhibitor) can exert synergistic cell growth inhibitory effects against MV4-11 cells. The synthetic route to the dual inhibitor embarked with the PdCl_2_(dppf) catalysed synthesis of a key intermediate (**41**) using (2,3-dichlorophenyl)boronic acid (**39**) and 3-bromo-6-chloropyrazin-2-amine (**40**). The aforementioned Suzuki cross-coupling reaction was carried out in 1,4-dioxane/H_2_O at 90 °C for 12h. Potassium phosphate was used as a base in the reaction to deprotonate the organoboron reagent and enable the formation of an active palladium complex. The aforestated reaction conditions led to the generation of adduct (**41**) in high yields, ensuring the possibility of late-stage modifications that allowed the research groups to install diverse linkers in the chemical architecture of the target scaffolds. Thus, a linker-cytotoxicity relationship as well as a linker-enzyme inhibition relationship could be developed that led to singling out of adduct (**38**) as the most balanced bifunctional inhibitor of SHP2 and HDAC. Impressively, the dual inhibitory structural template elicited striking antitumor activity in the *in vitro* as well as the *in vivo* studies. Also, treatments led to the activation of T cells, leading to antigen presentation function and promotion of cytokine secretion, thereby triggering antitumor immunity. Correlation of the antitumor activity evaluation results and the enzymatic assay outcomes confirmed dual SHP2 and HDAC inhibition as the underlying mechanism for these effects^117^.

**Figure 11.**
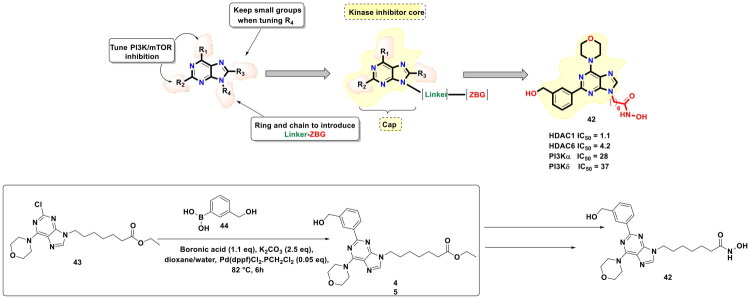
Design Strategy & Synthetic Route of Dual PI3K/Akt/mTOR-HDAC Inhibitor (the figure was drawn by the authors using chemdraw software).

Revelations from studies confirming the activation of PI3K/Akt/mTOR signalling pathway lead to resistance towards the cancer therapeutics, which led the authors to conceive the idea of attaining therapeutic benefits in HCC through dual modulation of PI3K and HDAC. Chen *et al.,* in quest to expand the activity spectrum of class I HDAC inhibitors for the treatment of hepatocellular carcinoma, designed dual PI3K/Akt/mTOR-HDAC inhibitors (as shown in Figure 11). To maximise the probability of identifying the most efficacious dual modulator, the research group planned to exercise variations in two components of the HDAC inhibitor pharmacophore, *viz.* Cap and Linker, while keeping hydroxamic acid as the constant structural feature in the entire compendium of compounds. In particular, a scout for the most appropriately functionalised purine core as the CAP construct of the HDAC inhibitory pharmacophore was conducted via stitching of diversely substituted phenyl rings on the purine core. The functionalised CAP components were furnished via application of the Suzuki cross-coupling reaction using Pd(dppf)Cl_2_·CH_2_Cl_2_ as the organopalladium catalyst. The functionalization was performed using a diversely substituted boronic acid in dioxane/water at 82 °C for 6h. Potassium carbonate was used as the base for this reaction. Extensive structural alteration attempted at the aforementioned site led the research group to furnish the most potent antitumor scaffold (**42**) in 87% yield. Delightfully, (**42**) demonstrated potent antitumor activity in hyper vascular liver cancer models along with a favourable target profile and tissue distribution^118^.

**Figure 12.**
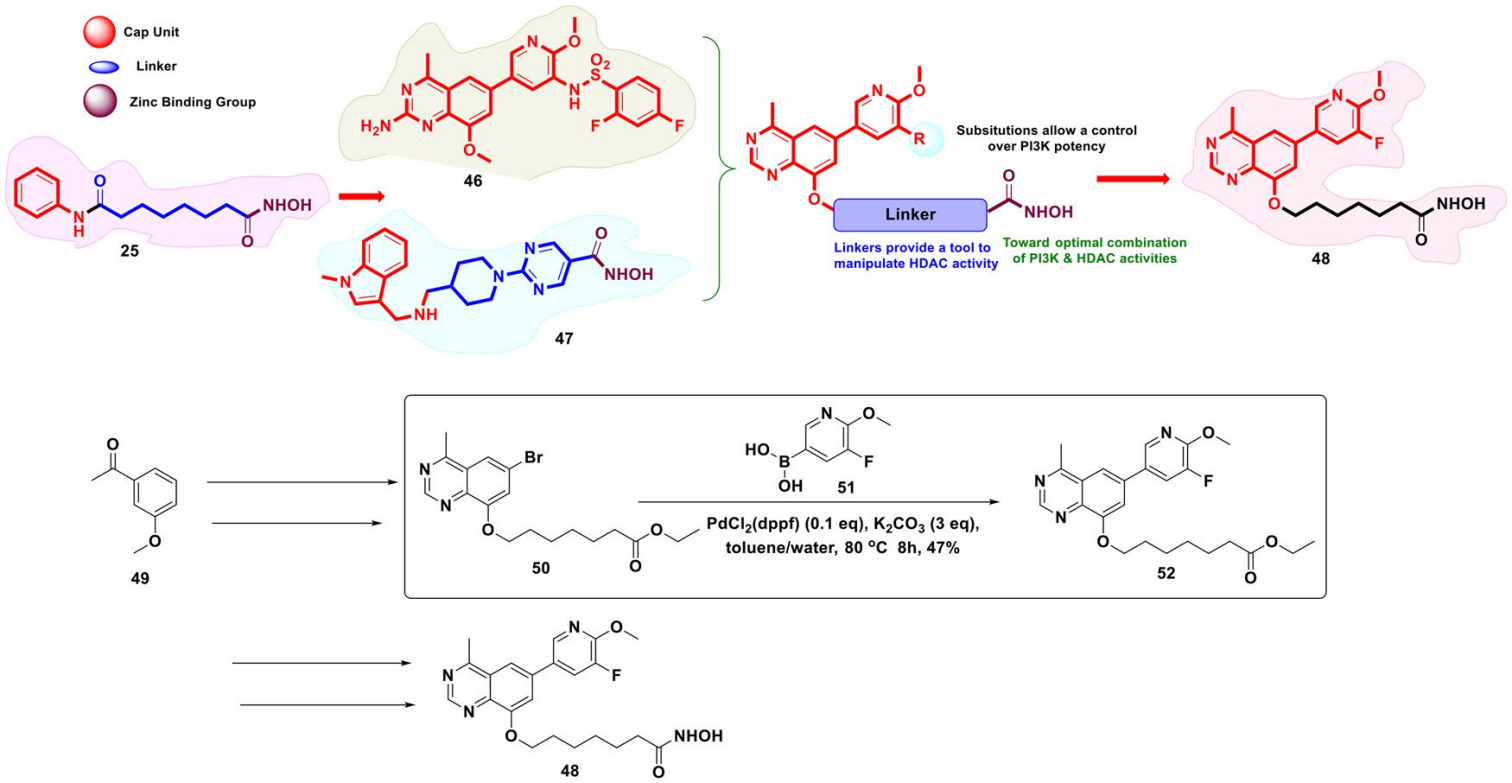
Design Strategy & Synthetic Route of Dual PI3K and HDAC Inhibitor (the figure was drawn by the authors using chemdraw software).

Another exemplary study for the design of dual PI3K and HDAC dual inhibitors was conducted by Zhang *et al*. The strategy used for the design of the target bifunctional scaffolds involved the tetheration of a zinc-binding group (hydroxamic acid) to a quinazoline scaffold via a linker, as shown in Figure 12. Organopalladium catalysis was utilised in this study at an advanced stage of the multi-step synthetic route for the furnishment of the most potent dual inhibitors (**48**). Specifically, PdCl_2_(dppf) was used for the C-C bond formation through Suzuki cross-coupling reaction between ethyl 7-((6-bromo-4-methylquinazolin-8-yl)oxy)heptanoate (**50**) and (5-fluoro-6-methoxypyridin-3-yl)boronic acid (**51**) at the penultimate step of the synthetic route to obtain the key intermediate to the target chemical architecture (**52**). The reaction was carried out in toluene/water at 80 °C for 8h, and potassium carbonate was used as the base. The yield of the Suzuki coupled product was only 47%; however, as this was the penultimate step of the synthetic scheme, the research groups proceeded ahead with the generation of the target compounds. Gratifyingly, the target compound was generated in sufficient amounts that allowed the team to explore the pharmacokinetics as well as the pharmacodynamics of the bifunctional scaffold^119^.

**Figure 13.**
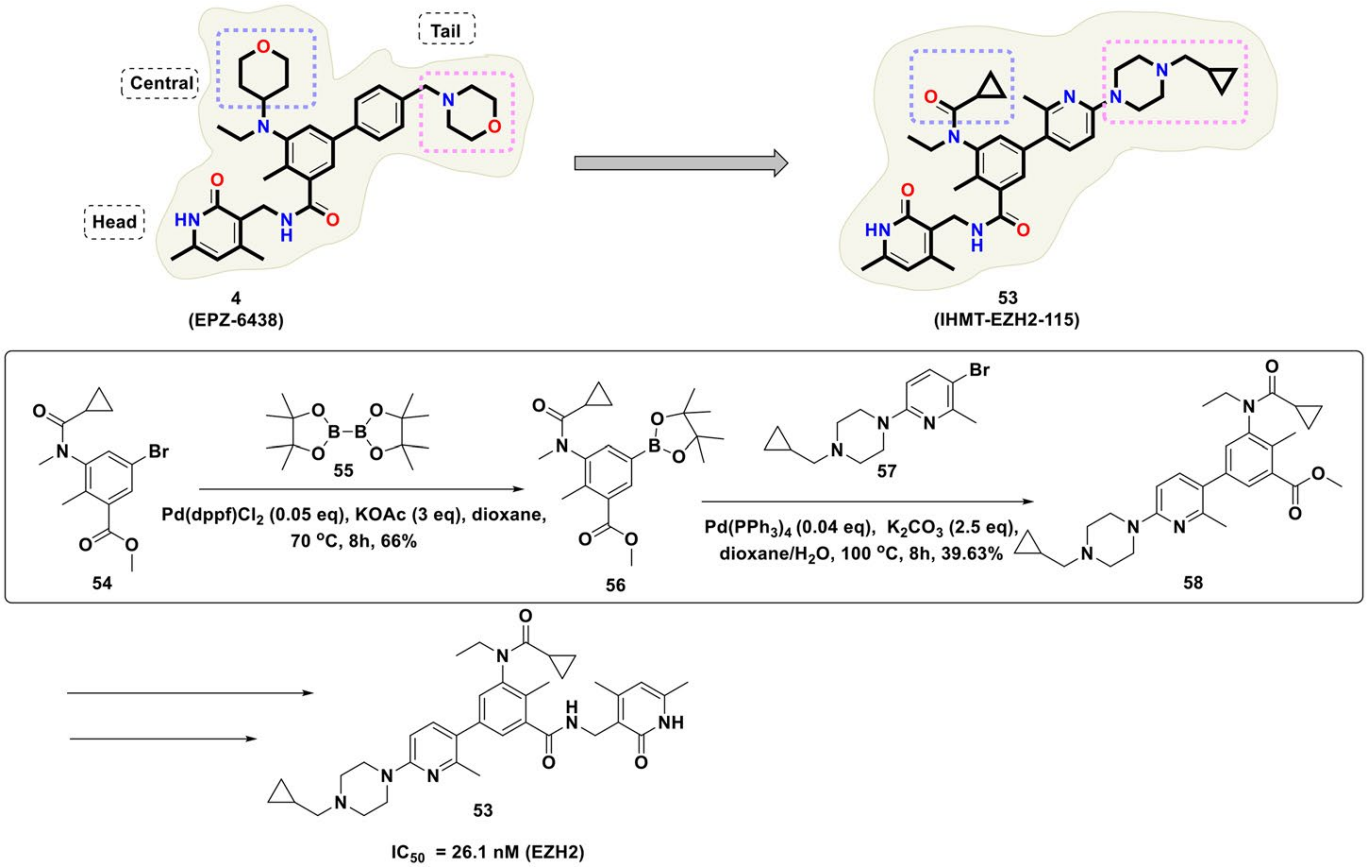
Design Strategy & Synthetic Route of Potent and Selective EZH2 Inhibitor (the figure was drawn by the authors using chemdraw software).

Zhou *et al.* conducted a medicinal chemistry campaign to develop EZH2 inhibitors for the treatment of hematological cancer, as shown in Figure 13. The structural template of the designed scaffold was inspired by the chemical architecture of EPZ-6438 (**4**) (Tazemetostat). The comprehensive exploration of the structural features culminated in the identification of a strikingly potent EZH2 inhibitor (**4**) (IC_50_ = 26.1 nM) that also displayed efficacy against EZH2 mutants (EZH2 Y641F, IC_50_ = 72.3 nM) and was endowed with favourable pharmacokinetic properties for oral administration. Also, the compound demonstrated antitumor efficacy in both Pfeiffer and Karpas-422 cell-mediated xenograft mouse models. Noteworthy to mention, the synthetic route to the designed inhibitor employed the utilisation of Pd(dppf)Cl_2_ and Pd(PPh_3_)_4_ for the first two consecutive steps. The synthetic route commenced with the conversion of methyl 5-bromo-3-(N-ethylcyclopropanecarboxamido)-2-methylbenzoate (**54**) to methyl 3-(N-ethylcyclopropanecarboxamido)-2-methyl-5-(4,4,5,5-tetramethyl-1,3,2-dioxaborolan-2-yl)benzoate (**56**) using Pd(dppf)Cl_2_. The reaction was performed in dioxane/H_2_O at 70 °C for 8h, and potassium acetate was used as the mild base in the reaction. The intermediate (**56**) was obtained in moderate yields (66%). Fourth, the construction of (**56**), the catalytic potential of tetrakis (triphenylphosphine)palladium (0) was exploited for the Suzuki cross-coupling reaction of (**56**) with 1-(5-bromo-6-methylpyridin-2-yl)-4-(cyclopropylmethyl)piperazine. Potassium carbonate was used as the base in the reaction, and the reaction was carried out in dioxane/H_2_O at 100 °C for 8h. The aforementioned reagents and reaction conditions produced the target compound (**53**) in 33% yield. Noteworthy to mention that this study underscores the reliance of the synthetic protocols to afford C-C bond formation^120^.

**Figure 14.**
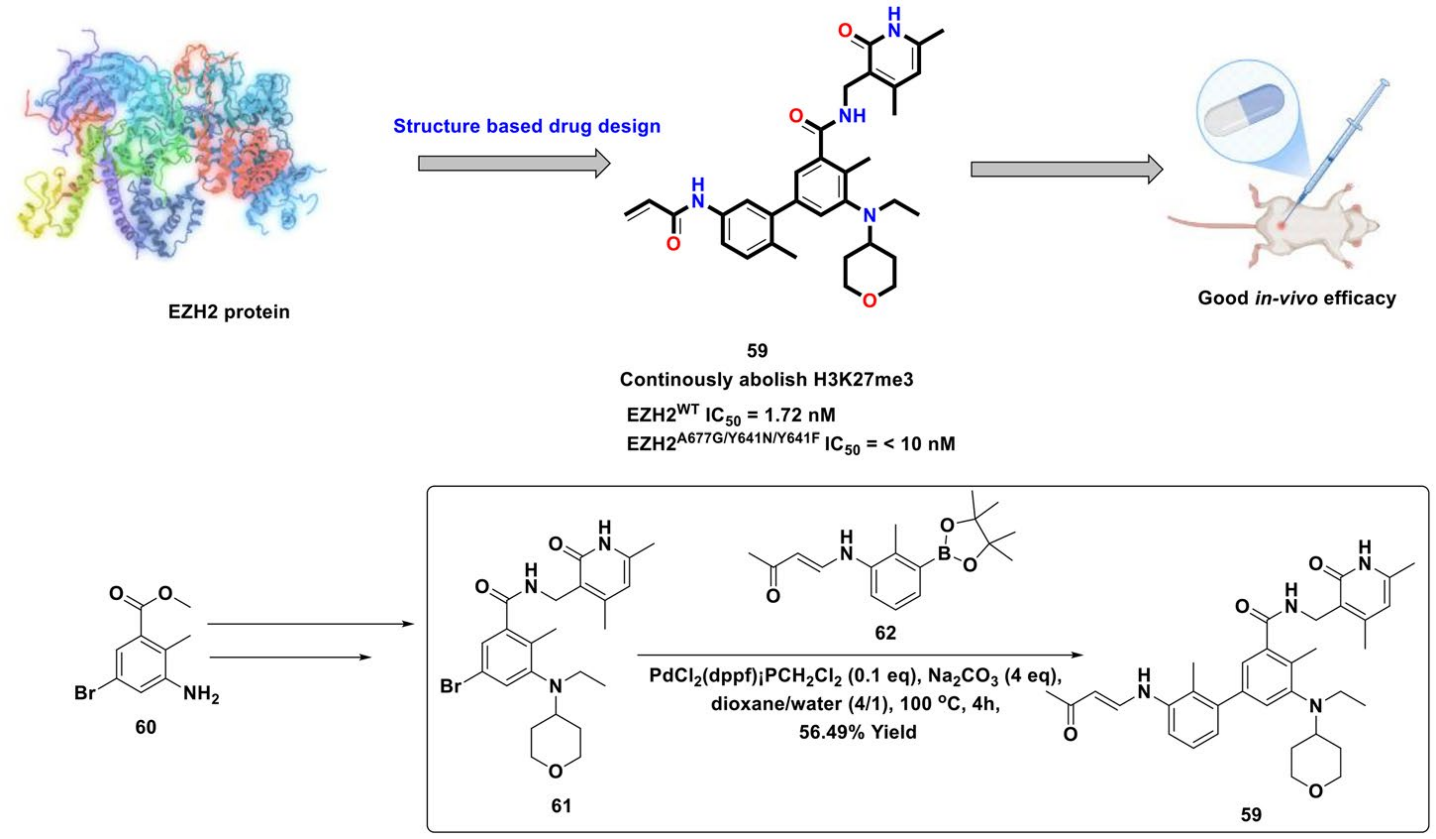
Design Strategy & Synthetic Route of Tazemetostat-Based Covalent EZH2 Inhibitor (the figure was drawn by the authors using chemdraw software).

Zhang *et al.* designed a covalent EZH2 inhibitor (**59**) based on the structural attributes of tazemetostat via installation of an acrylamide unit as the covalent reactive group (shown as in Figure 14). The synthetic pathway to the designed scaffold involved a reaction sequence comprising of conventional methodologies, *viz.,* reductive amination, amidation, and Suzuki cross-coupling reactions. Noteworthy to mention that the PdCl_2_(dppf)CH_2_Cl_2_ catalysed Suzuki cross-coupling reaction was used for the final step of the multistep synthetic route that enabled the research group to append the acrylamide bearing unit at the tetra-substituted phenyl ring. The reaction was carried out in dioxane/water (4/1) at 100 °C for 4h by utilising sodium carbonate as the base, and the target scaffold was attained in approximately 56.49% yield. It is important to mention that (**59**) was found to be highly effective against EZH2 mutants and demonstrated the ability to covalently bind to the S-adenosylmethionine (SAM) pocket of EZH2. Moreover, (**59**) elicited cell growth inhibitory effects against ovarian cancer cell lines and exerted tumour growth inhibition in the PA-1 xenograft model^121^.

**Figure 15.**
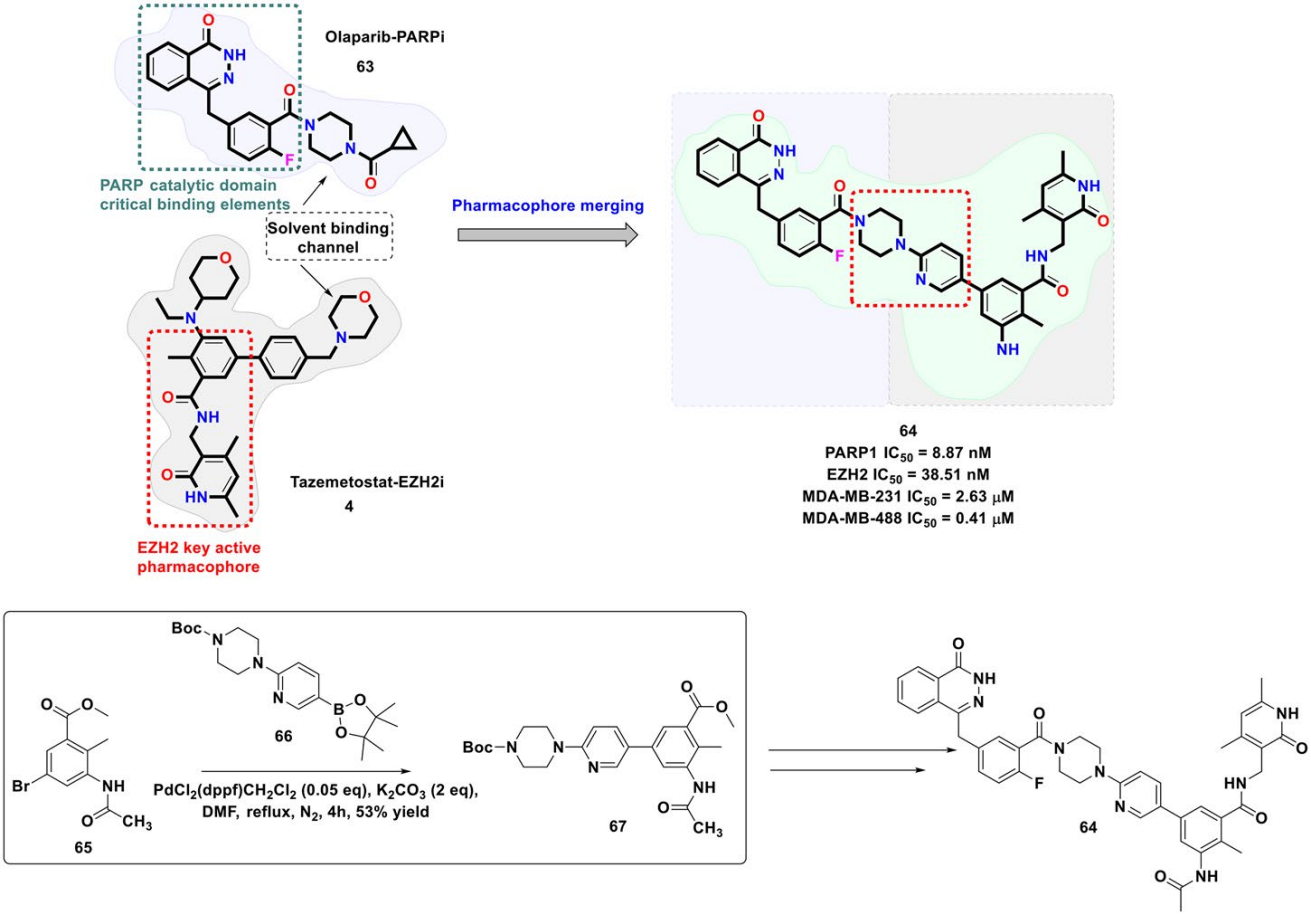
Design Strategy & Synthetic Route of First-in-Class Dual PARP and EZH2 Inhibitor for TNBC (the figure was drawn by the authors using chemdraw software).

In pursuit to expand the activity spectrum of PARP inhibitors to triple negative breast cancer cells (TNBC) without the BRCA mutations, Wan *et al.* embarked on a drug discovery endeavour to construct dual modulatory structural templates capable of inhibiting PARP and EZH2, as shown in Figure 15. The aforementioned plan was inspired by the precedential claims ascertaining the efficacy of EZH2 inhibitors to improve the sensitivity of wild-type BRCA cells to PARP inhibitors. For the design of the target compounds, the chemical architectures of Olaparib (**63**) (PARPi) and Tazemetostat (EZH2i) (**4**) were leveraged as the starting fragments. Encouragingly, a strikingly potent dual PARP-EZH2 inhibitor was furnished as part of this campaign via a synthetic route that commenced with an organopalladium-catalysed Suzuki cross-coupling reaction between methyl 3-bromobenzoate (**65**) and 4-(4-Boc-1-piperazinyl) phenyl borate pinacol ester (**66**). PdCl_2_(dppf)·CH_2_Cl_2_ and potassium carbonate were used as the organopalladium catalyst and the base, respectively. DMF was used as a solvent, and the reaction was performed under nitrogen protection for 4h. The aforementioned reaction conditions led to the generation of the key intermediate (**67**) in good yield, thereby enabling the authors to proceed forward and furnish the target dual inhibitory chemical architecture (**64**) through a series of reactions. Noteworthy to mention that the compound (**64**) demonstrated an impressive *in vitro* cytotoxicity profile against MDA-MB-231 (IC_50_ = 2.63 μM) and MDA-MB-468 (IC_50_ = 0.41 μM) cells with wild-type BRCA (TNBC cell lines). Correlation of cellular activity and enzymatic activity evaluation results revealed that the cytotoxic effects of the dual inhibitor (**64**) were mediated through balanced PARP1 and EZH2 inhibition. Moreover, the compound (**64**) was found to be an autophagy inducer^122^.

**Figure 16.**
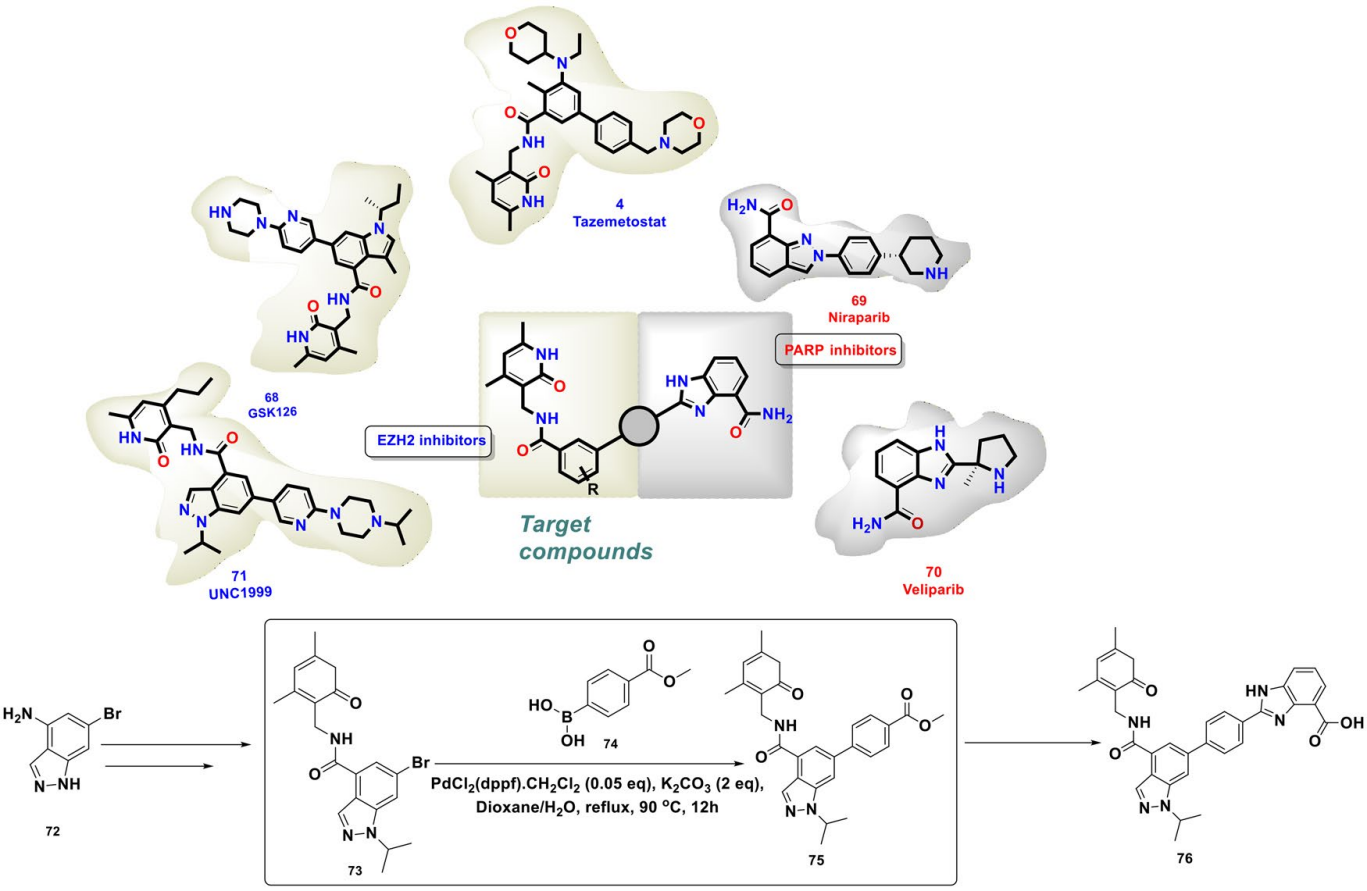
Design Strategy & Synthetic Route of Dual Target PARP1/EZH2 Inhibitor (the figure was drawn by the authors using chemdraw software).

**Figure 17.**
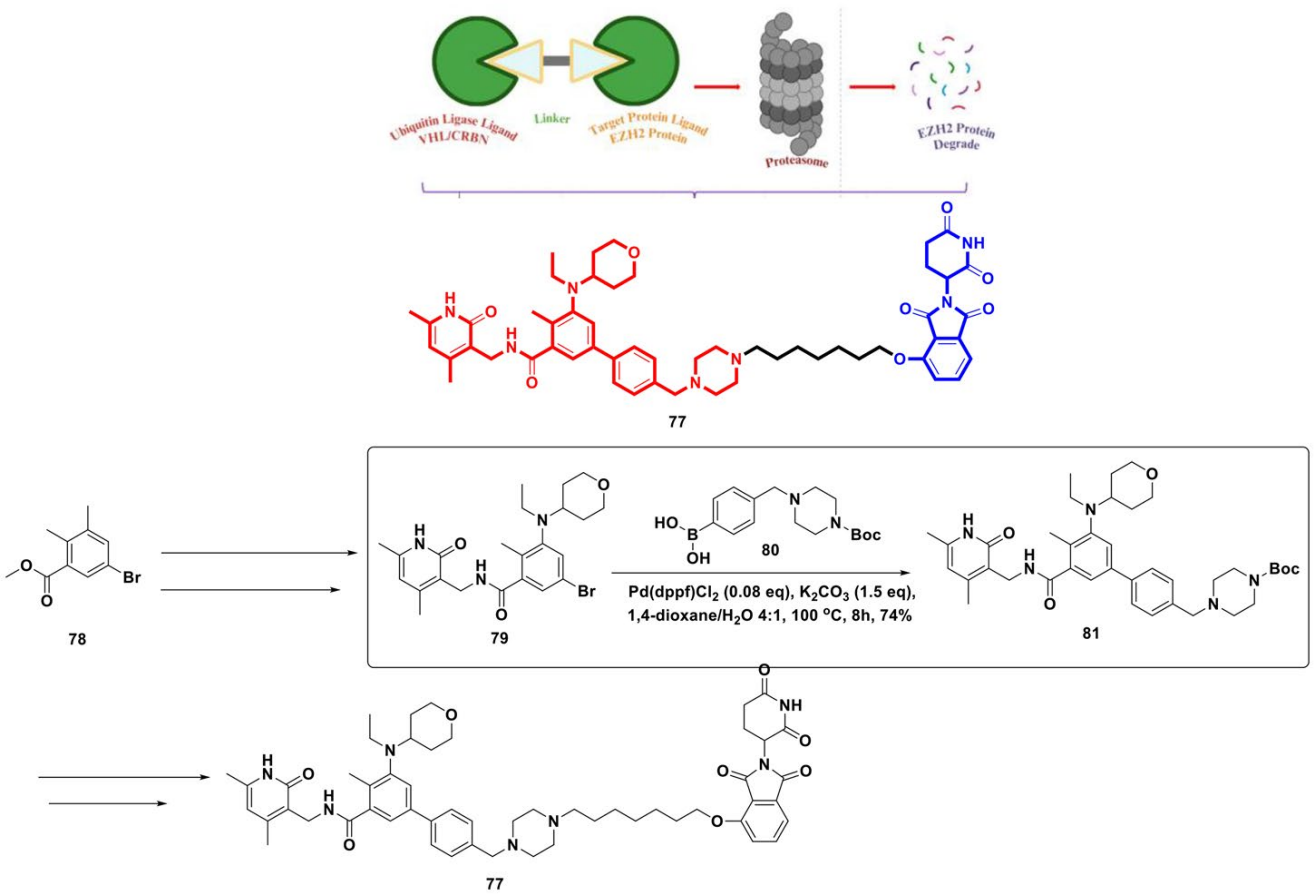
Design Strategy & Synthetic Route of EZH2 Based PROTAC (the figure was drawn by the authors using chemdraw software).

**Figure 18.**
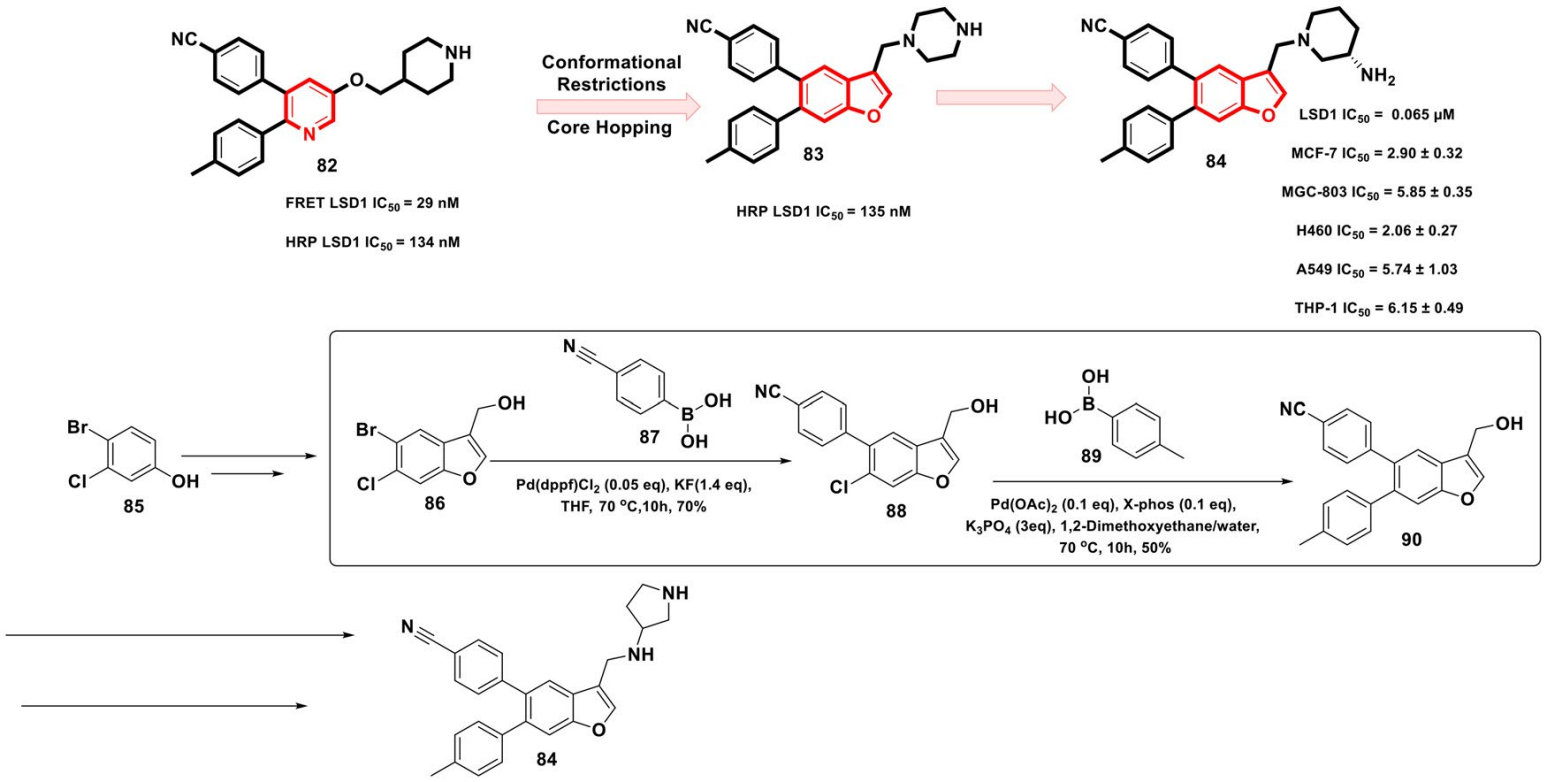
Design Strategy & Synthetic Route of Novel Benzofuran Derivatives as Potent LSD1 Inhibitors (the figure was drawn by the authors using chemdraw software).

**Figure 19.**
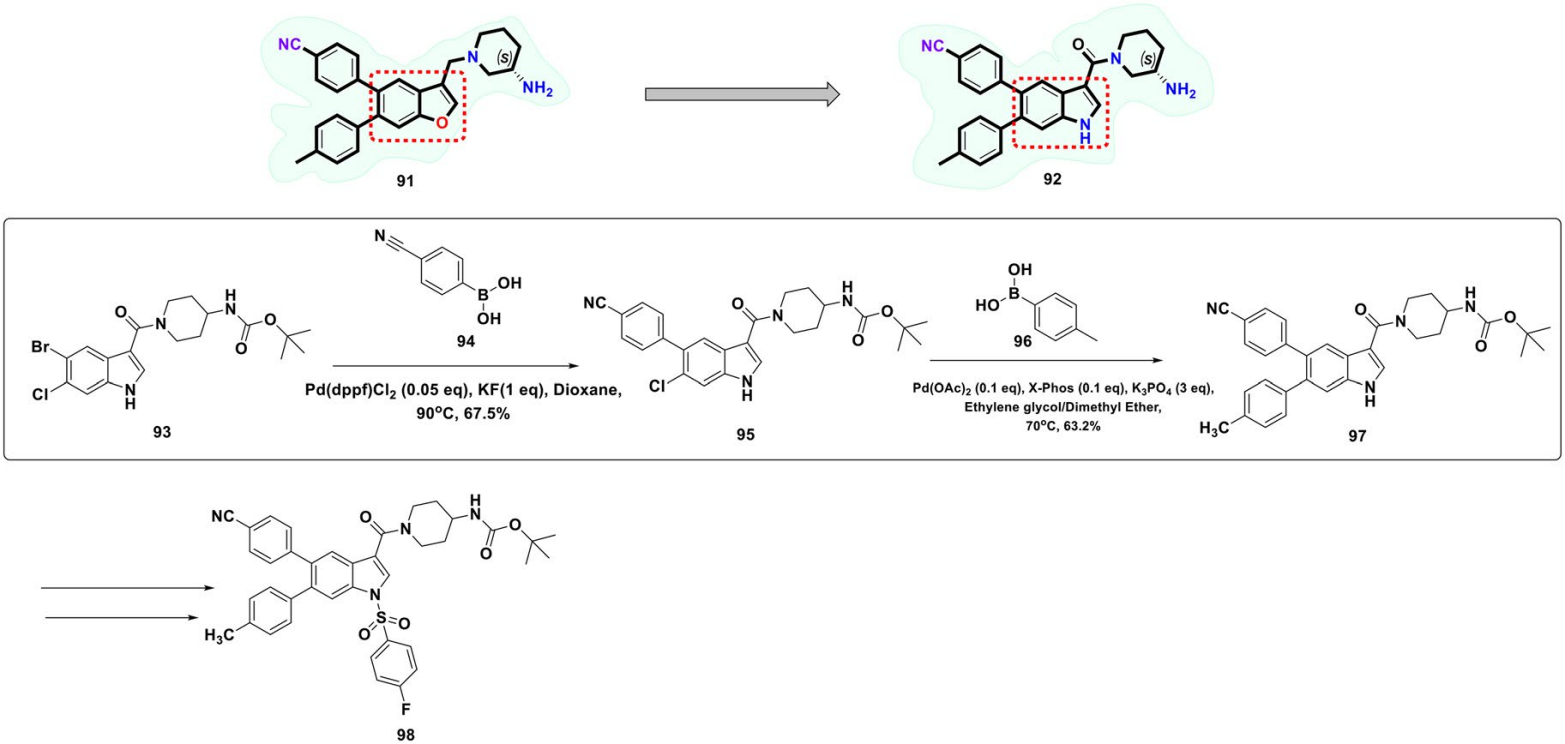
Design Strategy & Synthetic Route of Novel Indole Derivatives as LSD1 Inhibitors (the figure was drawn by the authors using chemdraw software).

Li *et al.* developed a synthetic route for the synthesis of the dual EZH2-PARP for the treatment of BRCA-mutated TNBC. The research group relied on palladium catalysis to stitch a substituted phenyl ring on the indazole ring (**72**) to construct a key intermediate (**73**) that was further utilised in the synthesis of the target compounds (**76**) (as shown in Fig. 16)^.^ The aforementioned stitching was accomplished via Suzuki cross-coupling reaction catalysed by PdCl_2_(dppf)⋅CH_2_Cl_2_. Notably, potassium carbonate was used as a base in this reaction, and a mixture of dioxane and water was used as the solvent for the reaction. The reaction mixture was refluxed for 12h to obtain intermediate (**75**). The dual PARP-EZH2 inhibitor demonstrated cytotoxicity against MDA-MB-231 cells (IC_50_ = 2.84 μM) and BT-549 cells (IC_50_ = 0.91 μM) mediated via dual inhibition of PARP1 (IC_50_ = 6.89 nM) and EZH2 (IC_50_ = 27.34 nM). The dual inhibitor (**76**) was also found to be endowed with *in vivo* antitumor activity, causing TGI of 57.24% in the tumour xenograft model, and also induced cell death via autophagy. The outcome of the biological evaluation indicated that the synthetic lethality attained by the dual inhibitor was attributed to EZH2 inhibition, which increased the sensitivity to PARP1^123^.

Liu *et al.* furnished a potent degrader (PROTAC) of EZH2 to extract therapeutic benefits of EZH2 targeting in cancer via proteasomal degradation of PRC2 components (shown in Figure 17). The three components of PROTAC models featuring a ligand for the target protein, a linker, and a ligand for E3 ligase, were leveraged for the design of a target degrader. Tazemetostat (**4**) was selected as the ligand for EZH2, and 4-hydroxythalidomide (CRBN Ligand) was pinpointed as an E3 ligase ligand to activate the ubiquitin proteasome pathway. The linker part was interrogated, and alkyl chains of varied lengths were used to tether the ligand for the target protein and the ligand for the E3 ligase. The most potent degrader was generated via a multistep synthetic route that commenced with the iron and ammonium chloride-assisted nitro reduction of methyl 5-bromo-2-methyl-3-nitrobenzoate (**78**), followed by consecutive reductive aminations, hydrolysis and EDCl/HOBt-mediated amidation to generate adduct (**79**). Interestingly, adduct (**79**) was leveraged for Suzuki cross-coupling reaction with 4-[(4-boc-1-piperazinyl)methyl]phenylboronic acid pinacol ester (**80**) to attain intermediate (**81**). Notably, Pd(dppf)Cl_2_ and potassium carbonate were used as the organopalladium catalyst and base for the reaction. The reaction was carried out in 1,4-dioxane and H_2_O (4:1) and was heated at 100 °C for 8h. The resulting adduct was subsequently subjected to Boc deprotection, followed by sodium bicarbonate-assisted alkylation with alkyl arms bearing E3 ligase ligands to generate target adducts (**77**). Noteworthy to mention that the Suzuki arylation yielded the intermediate in good yield (74% yield), ensuring the utilisation of the adduct for the generation of a compendium of compounds that allowed the research group to generate a linker–activity relationship. The aforementioned attempts proved to be instrumental in the identification of the standout PROTAC. Encouragingly, the target compound inhibited transcriptional silencing mediated by EZH2, dependent on PRC2, and manifested an impressive cytotoxicity profile against cancer cell lines^124^.

Zhang *et al.* accomplished a library of LSD1 inhibitors utilising the benzofuran scaffold and conducted an exhaustive investigation of the generated chemical architectures as depicted in Figure 18. Resultantly, a strikingly potent LSD1 inhibitor (**84**) (IC_50_ values = 0.065 µM) was identified that elicited striking antitumor effects against numerous cancer cell lines. Moreover, the compounds demonstrated apoptosis-inducing ability, *in vivo* antiproliferative activity in the H460 xenograft tumour model, and favourable liver microsomal stability. It is important to mention that the consecutive Suzuki cross-coupling reactions were performed as a part of the synthetic route optimised to afford the synthesis of the aforementioned LSD1 inhibitor. The protocol employed involved the conversion of 4-bromo-3-chlorophenol (**85**) to adduct (**86**), which was subjected to two C-C bond formation strategies via organopalladium catalysis. The first Suzuki coupling leveraged the catalytic potential of Pd(dppf)Cl_2_ to enable the cross-coupling between 4-cyanophenylboronic acid (**87**) and adduct (**86**). Potassium fluoride was used in this reaction to increase the nucleophilicity of boron in a quest to facilitate the transmetalation. Tetrahydrofuran was used as a solvent in the reaction, and the reaction was performed in an eggshell bottle under an argon atmosphere at 70 °C for 10h. The aforementioned reaction yielded the intermediate in good yields (70%). Noteworthy to mention that the subsequent cross-coupling reaction with 4-Methylphenylboronic acid (**89**) was not accomplished using Pd(dppf)Cl_2_, rather a different organopalladium catalyst, *viz.* palladium acetate, was used for the reaction. Tripotassium phosphate was used as the base in the reaction. The biaryl phosphine ligand (X-Phos) was used to stabilise the palladium catalyst and help in the oxidative addition step by rendering Pd(0) more nucleophilic. Notably, 1,2-dimethoxyethane/water (3:1) was used in the reaction, and the reaction was performed at 70 °C for 10h in an eggshell bottle. The yield of the reaction was reported to be around 50%, and the adduct (**90**) was obtained for further reaction to accomplish the formation of the target compound (**84**)^125^.

Zhang *et al.* continued working on the design and synthesis of LSD1 inhibitors and employed a lead modification approach to fine-tune the activity profile of compound (**84**), as shown in Figure 19. Bioelectronic isosteric strategy was used for the structure-based optimisation. Resultantly, compound (**98**) was furnished that exerted striking *in vitro* anti-lung cancer effects mediated via LSD1 inhibition (IC_50_ = 0.050 ± 0.005 μM). Also, the compound exhibited favourable metabolic stability and remarkable antitumor effects in *in vivo* studies. Further explorations revealed that IGFBP3 expression was enhanced, and the PI3K/Akt pathway and YAP-Hippo pathway were activated with compound treatment. The aforementioned observations underscore the potential of compound (**98**) to regulate genes associated with transcriptional dysregulation in cancer. Similar to compound (**85**), the synthetic pathway to compound (**98**) leveraged the catalytic potential of Pd(dppf)Cl_2_ and Palladium(II) acetate to catalyse consecutive Suzuki arylation with 4-cyanobenzeneboronic acid and 4-tolylboronic acid. Potassium carbonate was used for the former reaction, and potassium phosphate was used for the latter. Both the Suzuki cross-coupling reactions yielded the intermediates (**95** and **97**) in acceptable yields (67.5 and 63.2%), respectively, and this enabled the research group to further perform sulfonylation reaction with intermediate (**97**) and afford the formation of the target compound (**98**)^126^.

**Figure 20.**
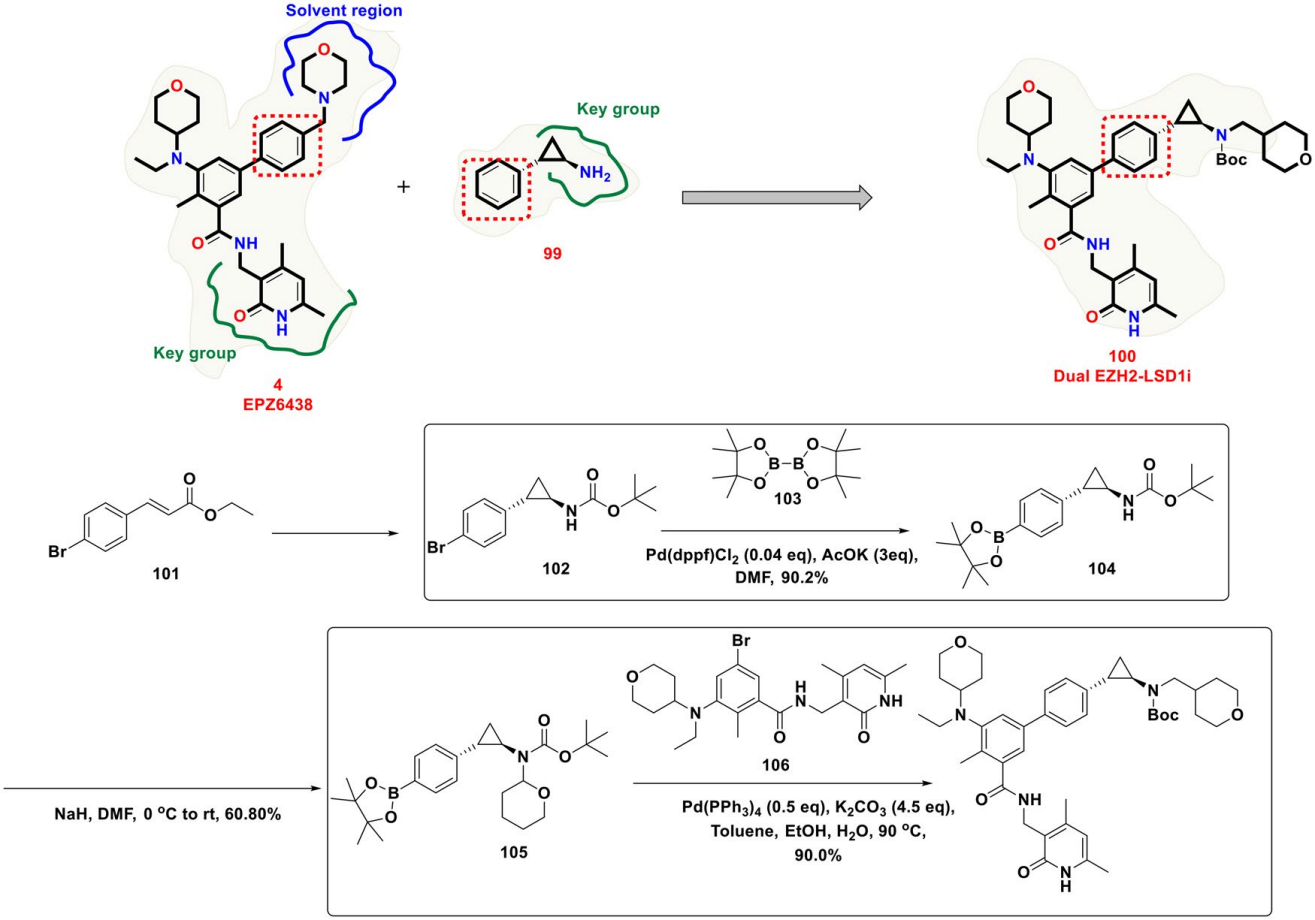
Design Strategy & Synthetic Route of Dual EZH2 & LSD1 Inhibitor (the figure was drawn by the authors using chemdraw software).

Synergistic antiproliferation effects of LSD1 inhibitors and EZH2 inhibitors stem from the revelations regarding the mediation of H3K27 trimethylation by EZH2 and the removal of methyl groups from H3K4me1/2 and H3K9me1/2. Banking on these revelations, Le *et al.* furnished a series of EZH2/LSD1 dual inhibitors and subjected them to exhaustive biological evaluations, as shown in Figure 20. The outcome of the biological evaluations led to the identification of a dual EZH2/LSD1 inhibitor (**100**) that was endowed with excellent antiproliferative activity. Interestingly, western blot analysis results indicated that the compound (**100**) could lead to the accumulation of H3K4me2 and H3K9me2 and decrease H3K27me3 expression. Tumour growth suppression ability of the compound was also evident in the *in vivo* studies (22RV1 xenograft mouse model). The multistep synthetic route to the compound (**100**) involved an organopalladium-catalysed Suzuki cross-coupling reaction for mediating two key steps. As shown in the scheme, the conversion of (**102**) to (**104**) was attained by the reaction with bis(pinacolato)diboron (**103**). Pd(dppf)Cl_2_ was used as the organopalladium catalyst, and potassium acetate was employed as the mild base for the transmetallation. The reaction was performed under an argon atmosphere and stirred at 90 °C for 12h in DMF. Gladly, the yield of the reaction was exceptionally high (>90%) mixture was stirred at 90 °C under an argon atmosphere for 12h. Fourth, the successful generation of the adduct (**104**), sodium hydride-mediated appendage of the tetrahydropyranyl group at the carbamate nitrogen, and the resulting adduct (**105**) was again subjected to a Suzuki cross-coupling reaction. For this step, Pd(PPh_3_)_4_ was used as the organopalladium catalyst. Toluene:EtOH:H_2_O = 3:1:2 (6 ml) was used as the solvent system for the reaction, and potassium carbonate was used as the base for the reaction. Similar to the first Suzuki coupling reaction, this step was also performed (constant stirring) under an argon atmosphere at 90 °C for 12h. The yield of this reaction was around 89%. Noteworthy to mention that the exceptionally high-yielding Suzuki cross-coupling reaction of the synthetic protocol ensured that the target compound could be attained in high yields, which enabled the research group to conduct a comprehensive biological evaluation^127^.

**Figure 21.**
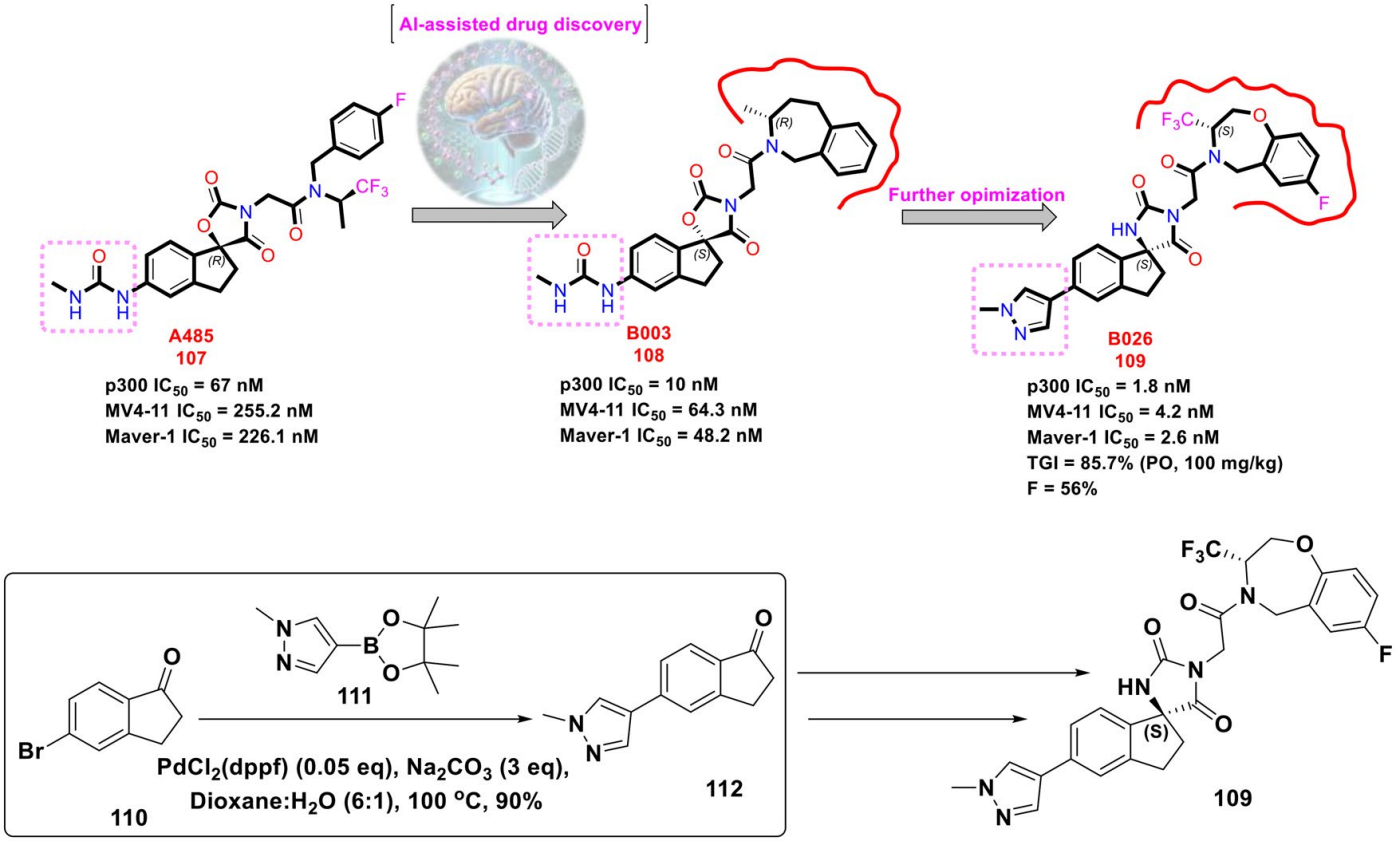
Design Strategy & Synthetic Route of Highly Potent p300/CBP Histone Acetyltransferases Inhibitors (the figure was drawn by the authors using chemdraw software).

Yang *et al.* designed and furnished a dual p300/CBP HAT inhibitor in pursuit of attaining synergistic antiproliferative effects (as shown in Figure 21). Disclosures ascertaining the prominent role of p300 and CREB-CBP as transcriptional activators for numerous cellular processes spurred the research group to generate a scaffold capable of inducing balanced modulation of p300 and CBP. The authors used an artificial-intelligence-assisted drug discovery pipeline to identify a starting point that was subjected to exhaustive structural optimisation. Resultantly, a potent dual inhibitor p300/CBP HAT (**109**) was pinpointed [IC_50_ value = 1.8 nM (p300) and 9.5 nM (CBP enzyme)]. The dual inhibitor also exerted dose-dependent tumour growth inhibition in an animal model of human cancer. Noteworthy to mention that the synthetic route to the target dual inhibitor (**109**) commenced with a Suzuki − Miyaura cross-coupling reaction of 5-bromo-1-indanone (**110**) with 1-methyl-4-(4,4,5,5-tetramethyl-1,3,2-dioxaborolan-2-yl)-1H-pyrazole to furnish a key intermediate (**112**). Moreover, PdCl_2_(dppf) was used as the organopalladium catalyst and sodium carbonate as the base. Dioxane − H_2_O was used as the solvent mixture, and the reaction mixture was bubbled with argon gas and then heated to 100 °C in an oil bath for 17h. It is noteworthy to mention that the organopalladium-catalysed Suzuki cross-coupling reaction yielded the intermediate (**112**) in high yields and allowed the research group to subject intermediate (**112**) to direct spirocyclization followed by potassium carbonate-mediated alkylation to generate a target compound (**109**)^128^.

**Figure 22.**
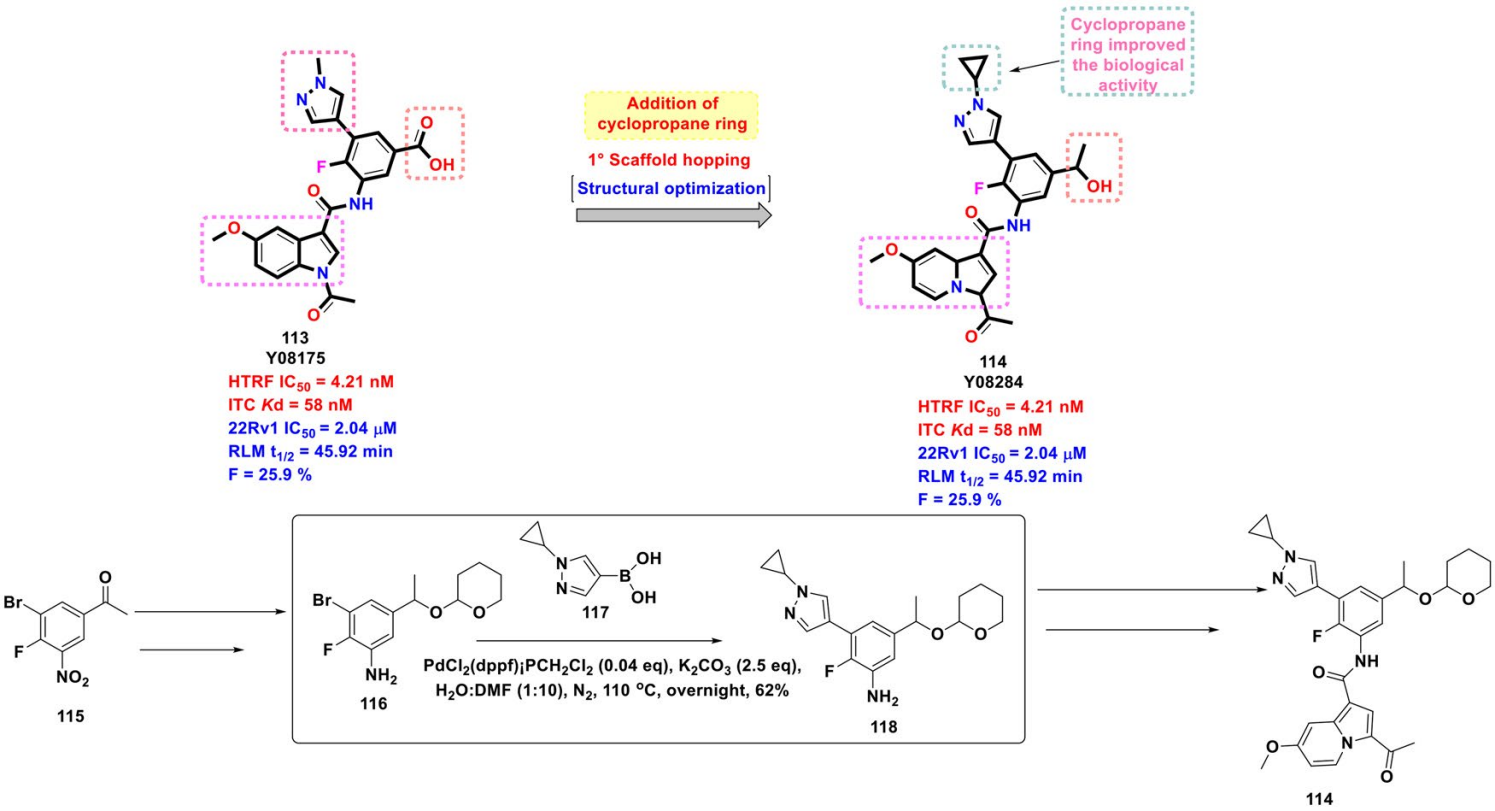
Design Strategy & Synthetic Route of 1-(Indolizin-3- yl) ethan-1-ones Based CBP Bromodomain Inhibitors (the figure was drawn by the authors using chemdraw software).

Xiang *et al.* furnished a selective CBP bromodomain inhibitor through structural optimisation of a previously reported CBP bromodomain inhibitor, as shown in Figure 22. The aim of conducting this medicinal chemistry campaign was to improve the cellular potency as well as the metabolic stability of the lead compound. The exhaustive structural optimisation program led to the identification of an embellished CBP bromodomain inhibitor. Notably, the multistep synthetic route to the compound leveraged the catalytic potential of an organopalladium to catalyse the Suzuki cross-coupling reaction to afford the formation of a key intermediate. The synthetic route commenced with the conversion of 1-(3-bromo-4-fluoro-5-nitrophenyl)ethan-1-one (**115**) to 3-bromo-2-fluoro-5-(1-((tetrahydro-2H-pyran-2-yl)oxy)ethyl)aniline (**116**) via a reaction sequence comprising steps *viz*. sodium borohydride reduction, DHP protection, nitro reduction. The adduct (**116**) attained through the aforementioned reaction sequence was utilised for C-C bond formation with (1-cyclopropyl-1H-pyrazol-4-yl)boronic acid. Interestingly, PdCl_2_(dppf)·CH_2_Cl_2_ was used as an organopalladium catalyst, and potassium carbonate was used as the base. The reaction was carried out in DMF under a nitrogen atmosphere at 110 °C for overnight, with the yield of the adduct (**118**) reported to be 62%. Noteworthy to mention that the aforementioned key step enabled the research group to install a pyrazole ring, which was the key structural feature of the lead compound. Further, the adduct was subjected to various chemical steps to afford the formation of the target CBP bromodomain inhibitor (**114**). Encouragingly, compound demonstrated good liver microsomal stability and pharmacokinetic properties and could inhibit CBP bromodomain as well as the proliferation of prostate cancer cells. In the *in vivo* studies, compound (**114**) also exhibited remarkable tumour growth inhibition (TGI = 88%) in a 22Rv1 xenograft model^129^.

**Figure 23.**
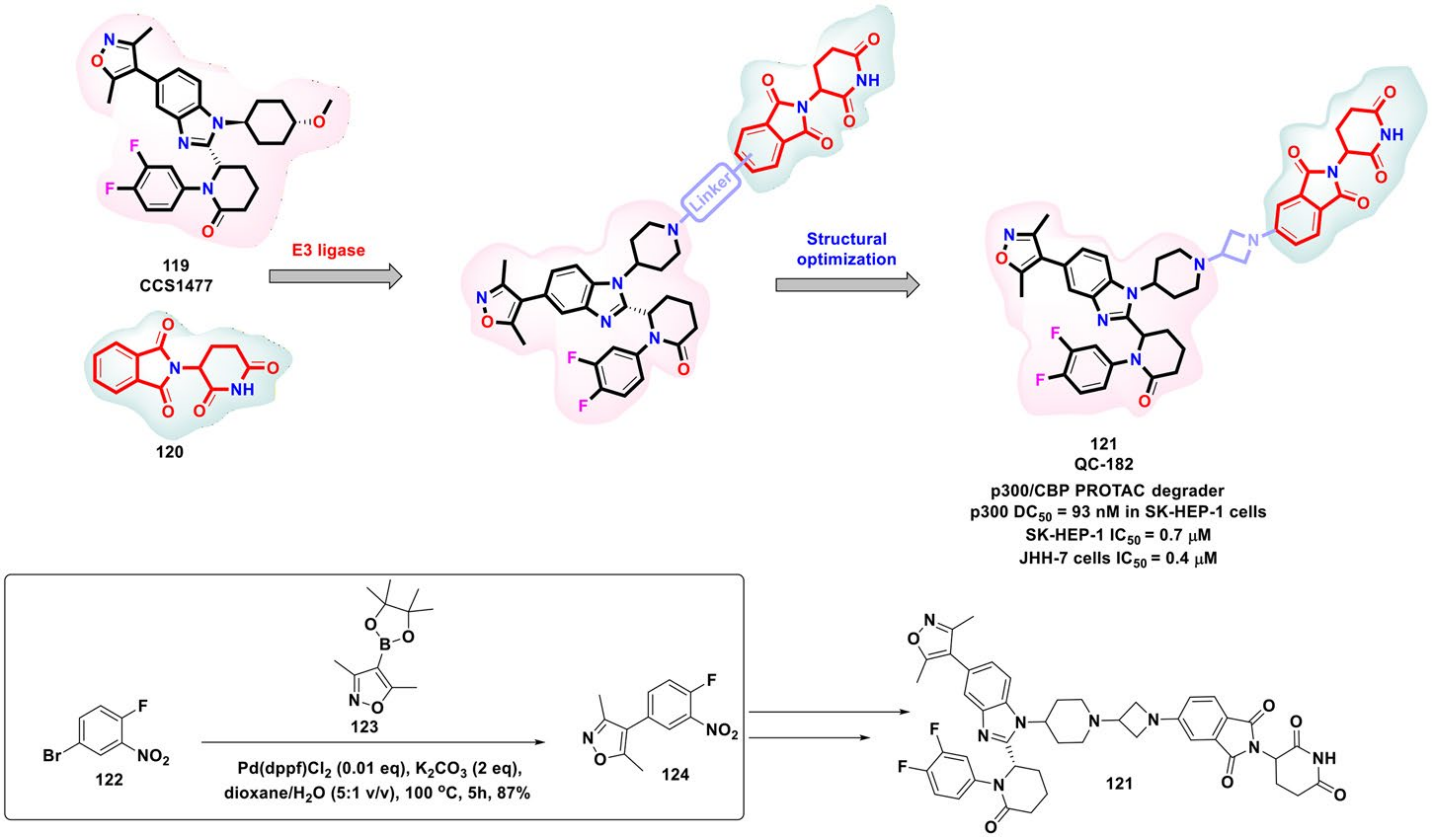
Design Strategy & Synthetic Route of Novel PROTAC Degraders of p300/CBP (the figure was drawn by the authors using chemdraw software).

Chang *et al.* designed a series of p300/CBP targeting PROTACs through the appendage of p300/CBP bromodomain inhibitor, CCS1477, to the binding scaffold of CRL4CRBN E3 ligase complex (thalidomide) for the design of putative p300/CBP PROTAC degraders through chemically diverse linkers (as shown in Figure 23). The outcome of the biological evaluation underscored the antitumor potential of one of the PROTAC (**121**) that demonstrated a striking ability to suppress p300/CBP-regulated transcriptome and exert p300/CBP protein degradation at low concentration in the SK-HEP-1 HCC cell line. Also, effective depletion of both p300 and CBP protein levels in SK-HEP-1 xenografts mouse tumour tissue was observed with compound (**121**) treatment. Given the aforementioned, PROTAC (**121**) seems to be a promising prospective therapeutic for the treatment of HCC. It is noteworthy to mention that organopalladium catalysis was leveraged for the synthesis of the ligand for the target protein. The first step of the synthetic route to the target protein inhibitor commenced with the Pd(dppf) Cl_2_-catalysed Suzuki cross-coupling reaction between 4-bromo-1-fluoro-2-nitrobenzene (**122**) and 3,5-dimethyl-4-(4,4,5,5-tetramethyl-1,3,2-dioxaborolan-2-yl)isoxazole (**124**), where potassium carbonate was used as the base and a mixture of dioxane and water was used as the solvent for this C-C bond formation reaction. The reaction was purged with argon and carried out for 5h at 100 °C. The yield of the reaction was good (87%), and the obtained adduct (**124**) was further subjected to a multistep synthetic route to furnish the target degrader (**121**)^130^.

**Figure 24.**
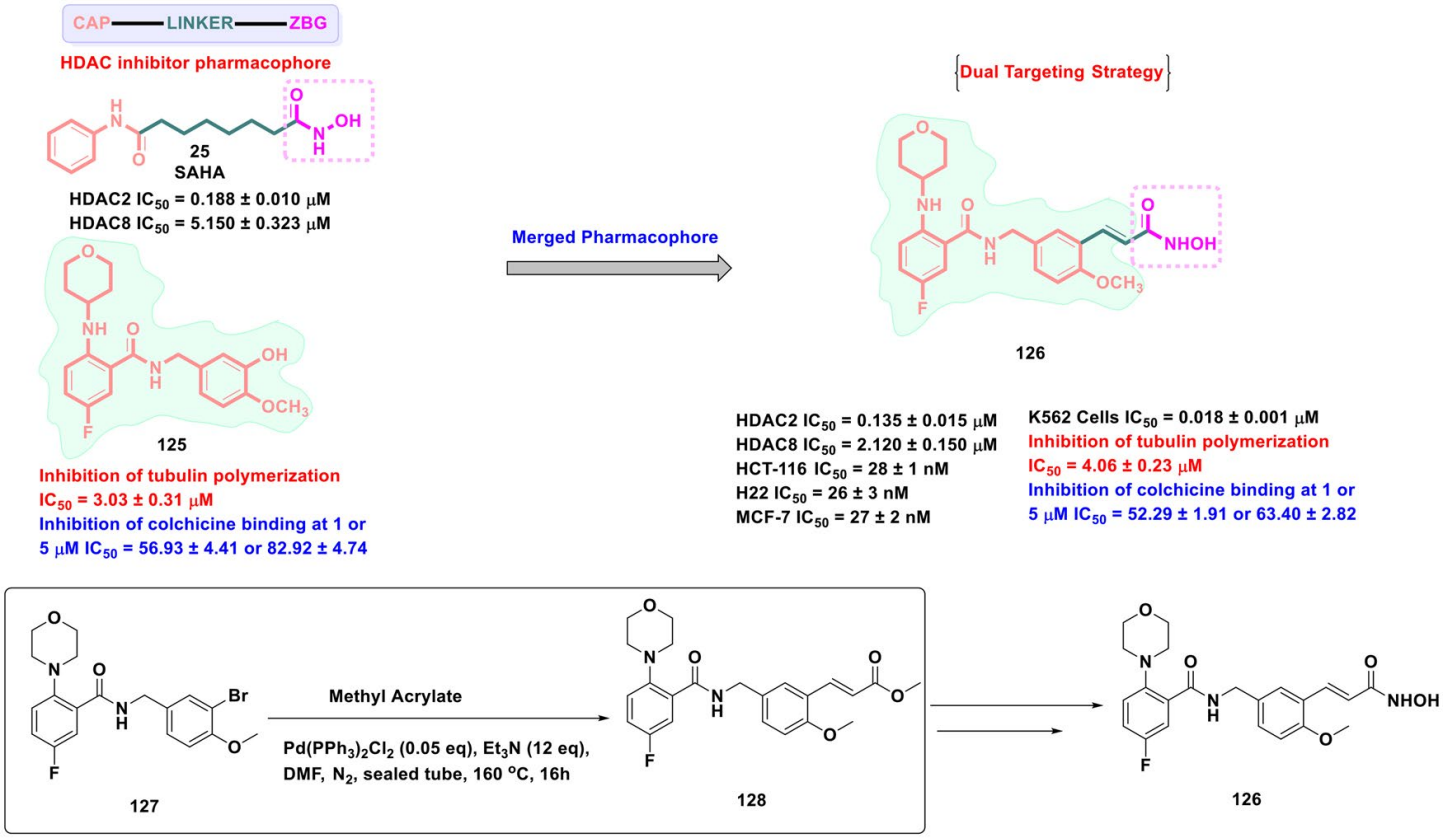
Design Strategy & Synthetic Route of Dual Tubulin and HDAC Inhibitor (the figure was drawn by the authors using chemdraw software).

Zhu *et al.* designed a series of dual Tubulin-HDAC inhibitors utilising the three-component HDAC inhibitor pharmacophore (Figure 24). The design strategy involved the appendage of a previously reported tubulin inhibitor to the zinc binding motif (hydroxamic acids and benzamides). Notably, the structural tolerability of the tubulin inhibitor served as the prominent reason behind its selection as a cap construct of the HDAC inhibitor model. The aforementioned envisionment culminated in the identification of a balanced dual tubulin–HDAC inhibitor (**126**). The synthetic route to the dual inhibitor employed the Heck olefination reaction for the generation of a key intermediate (**128**). N-(3-bromo-4-methoxybenzyl)-5-fluoro-2-morpholinobenzamide (**127**) was subjected to a Heck coupling reaction with methyl acrylate to form the desired acrylate intermediate (**128**). Pd(PPh_3_)_2_Cl_2_ was used as the organopalladium catalyst, and triethylamine was used as the base in this reaction. The reaction was performed in a sealed tube under a nitrogen atmosphere for 16h to produce (**128**). Noteworthy to mention, the previous examples covered in this section rely on Pd(dppf)Cl_2_ as the organopalladium catalyst. However, in this case, Pd(PPh_3_)Cl_2_ was used. A relative comparison of the aforementioned organopalladium indicates that Pd(dppf)Cl_2_ contains 1,1′-bis(diphenylphosphino)ferrocene (dppf) as a bidentate ligand, whereas Pd(PPh_3_)_2_Cl_2_ comprises two triphenylphosphine ligands (monodentate). In general, dppf has stronger electron-donating properties than PPh_3,_ which facilitates the oxidative addition. Though Pd (dppf)Cl_2_ holds the aforementioned advantage over Pd(PPh_3_)_2_Cl_2_, ease of dissociation to generate the active palladium species Pd(0) offered by the latter, owing to the presence of monodentate ligands, might have spurred the authors to employ Pd(PPh_3_)_2_Cl_2_ as the organopalladium catalyst. Notably, the aforementioned is an assumption as the authors have not mentioned the reason for their precise prioritisation of Pd(PPh_3_)_2_Cl_2_ over Pd (dppf)Cl_2_. Compound (**128**) was further converted to the target hydroxamic acid **126** via treatment with NH_2_OK in MeOH. Impressively, (**126**) exhibited a remarkable antitumor profile against a panel of cancer cell lines mediated via dual modulation of tubulin and HDAC. Also, the compound disrupted both internal and peripheral tumour vasculature, enhanced the acetylation level of HDAC substrate proteins, induced cell apoptosis, depolarised the mitochondria membrane potentials, induced ROS generation in HepG2 cells, and elicited striking *in vivo* antitumor activity in a liver cancer allograft mouse model. Specifically, treatment with compound (**126**) at the dose of 20 mg/kg led to significant suppression of tumour volume and reduction of tumour weight^131^.

**Figure 25.**
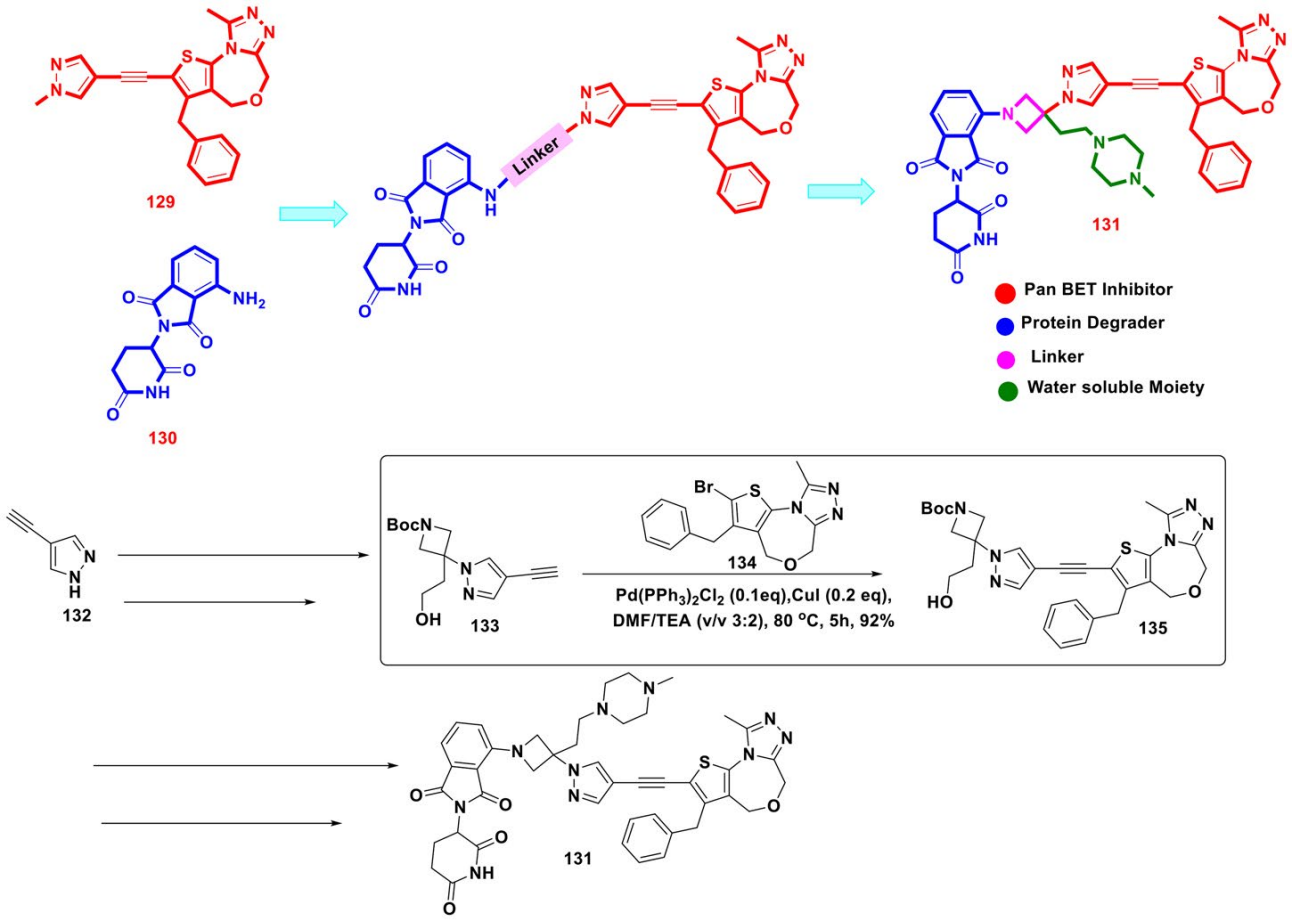
Design Strategy & Synthetic Route of Selective BRD4 Degraders (the figure was drawn by the authors using chemdraw software).

Hu *et al.* utilised the applications of Sonogashira coupling for the synthesis of a non-selective BET inhibitor. The authors utilised their previously reported PROTAC pan BET inhibitor, QCA276 (**129**), for the design of the target degraders (Figure 25). The route to the synthesis of the target compound commenced with the conversion of 4-ethynyl-1H-pyrazole (**132**) to tert-butyl 3-(4-ethynyl-1H-pyrazol-1-yl)-3-(2-hydroxyethyl)azetidine-1-carboxylate (**133**) via multiple steps. The adduct (**133**) was subjected to Sonogashira coupling using Pd(PPh_3_)_2_Cl_2_ as the organopalladium catalyst. Copper iodide was used as a co-catalyst in this reaction to form a copper acetylide intermediate that renders the alkyne more reactivity, thereby enabling its coupling with the organopalladium catalyst. Noteworthy to mention that the addition of copper iodine in Sonogashira reaction methodology generally ensures that the reaction can be accomplished at relatively low temperatures and with lower catalyst loadings. The reaction was performed in a mixture of DMF and TEA (3:2) at 80 °C for 5h. The aforementioned reaction conditions yielded the intermediate (**135**) in 92% yield. The high-yielding Sonogashira coupling protocol enabled the authors to further subject the intermediate (**135**) to a reaction sequence to afford the formation of the target compound. Gratifyingly, the compound (**131**) significantly depleted BRD4 protein in tumour tissues and remarkably inhibited the tumour growth (MV4;11 tumour tissues and MDA-MB-231 xenograft model). The PROTAC (**131**) rapidly degraded BRD4 protein at low concentrations^132^.

**Figure 26.**
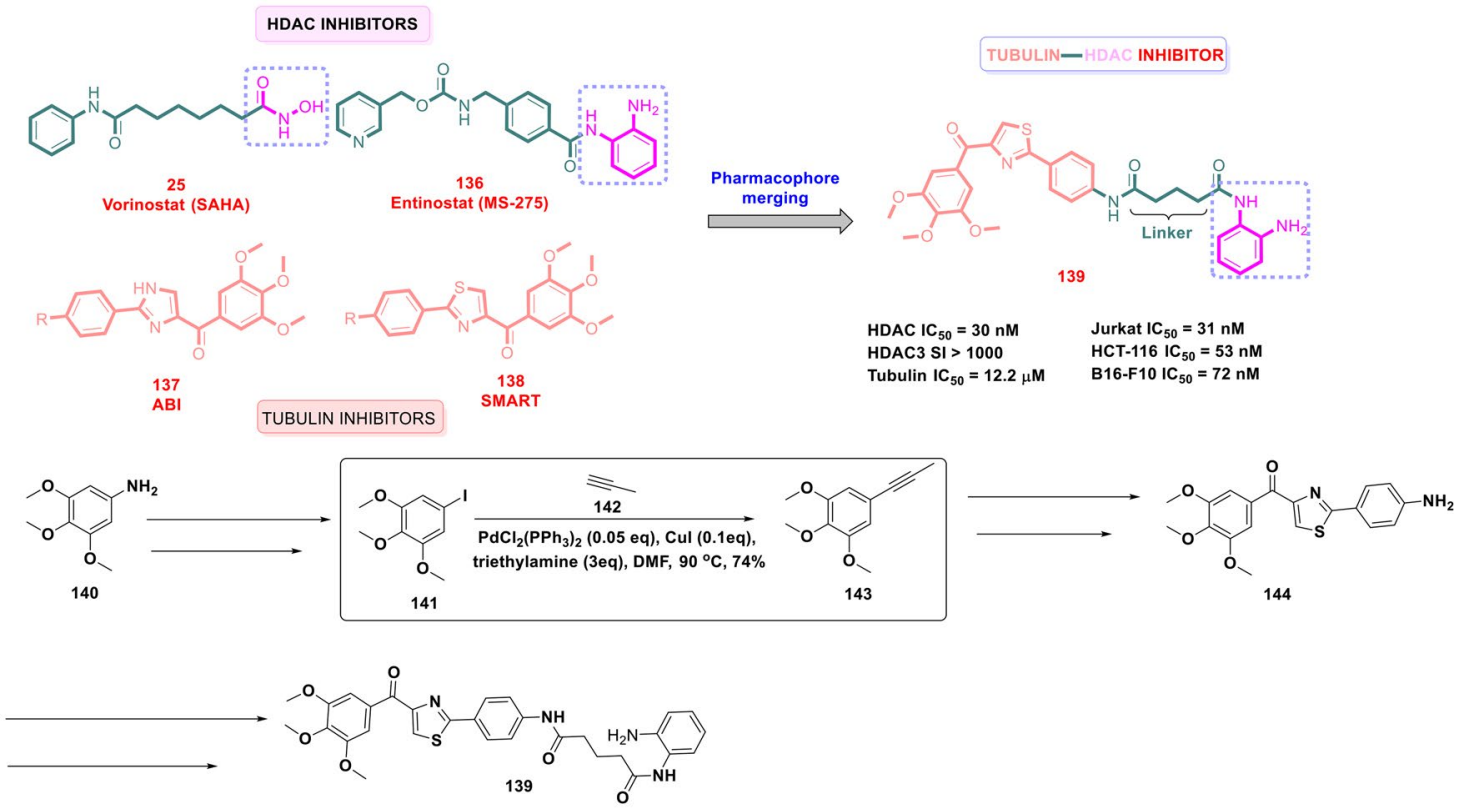
Design Strategy & Synthetic Route of Novel Dual Tubulin/Histone Deacetylase 3 Inhibitor (the figure was drawn by the authors using chemdraw software).

Sonogoshira coupling was also employed by PNEG *et al.* to generate a key intermediate that was further utilised for the construction of a dual tubulin-HDAC inhibitor (Figure 26). The chemical architecture of the target compound (**139**) comprised the pharmacophores of SMART (tubulin inhibitor) (**138**) and MS-275 (HDAC inhibitor) (**136**). The synthetic route to the target dual inhibitor (**139**) involved the amidation of a key intermediate (**144**) with long-chain alkoxy alkanoic acids to generate the desired dual modulatory action. Notably, the synthesis of the intermediate (**144**) was accomplished via a reaction sequence comprising a key step, *viz*., organopalladium-assisted coupling of 5-iodo-1,2,3-trimethoxybenzene (**140**) with prop-1-yne to produce 1,2,3-trimethoxy-5-(prop-1-yn-1-yl)benzene (**141**). The aforementioned C-C bond coupling reaction was accomplished using PdCl_2_(PPh_3_)_2_ as the organopalladium catalyst and copper iodide as a co-catalyst. Triethylamine was used as a base, and the reaction was carried out in DMF at 90 °C for 16h under a nitrogen atmosphere. The aforementioned conditions yielded a compound in 74%. This adduct was utilised to generate the target compound (**139**) via a multistep synthetic route. The resulting scaffold (**139**) demonstrated the ability to exert balanced modulation of HDAC3/tubulin and elicited cytotoxic potential against the HDAC-resistant gastric cancer cell line (YCC3/7) (IC_50_ = 30 − 144 nM). Moreover, striking antitumor efficacy was exhibited by compound (**139**) with a TGI of 70.00% at a dose of 10 mg/kg in a B16-F10 melanoma tumour model^133^.

**Figure 27.**
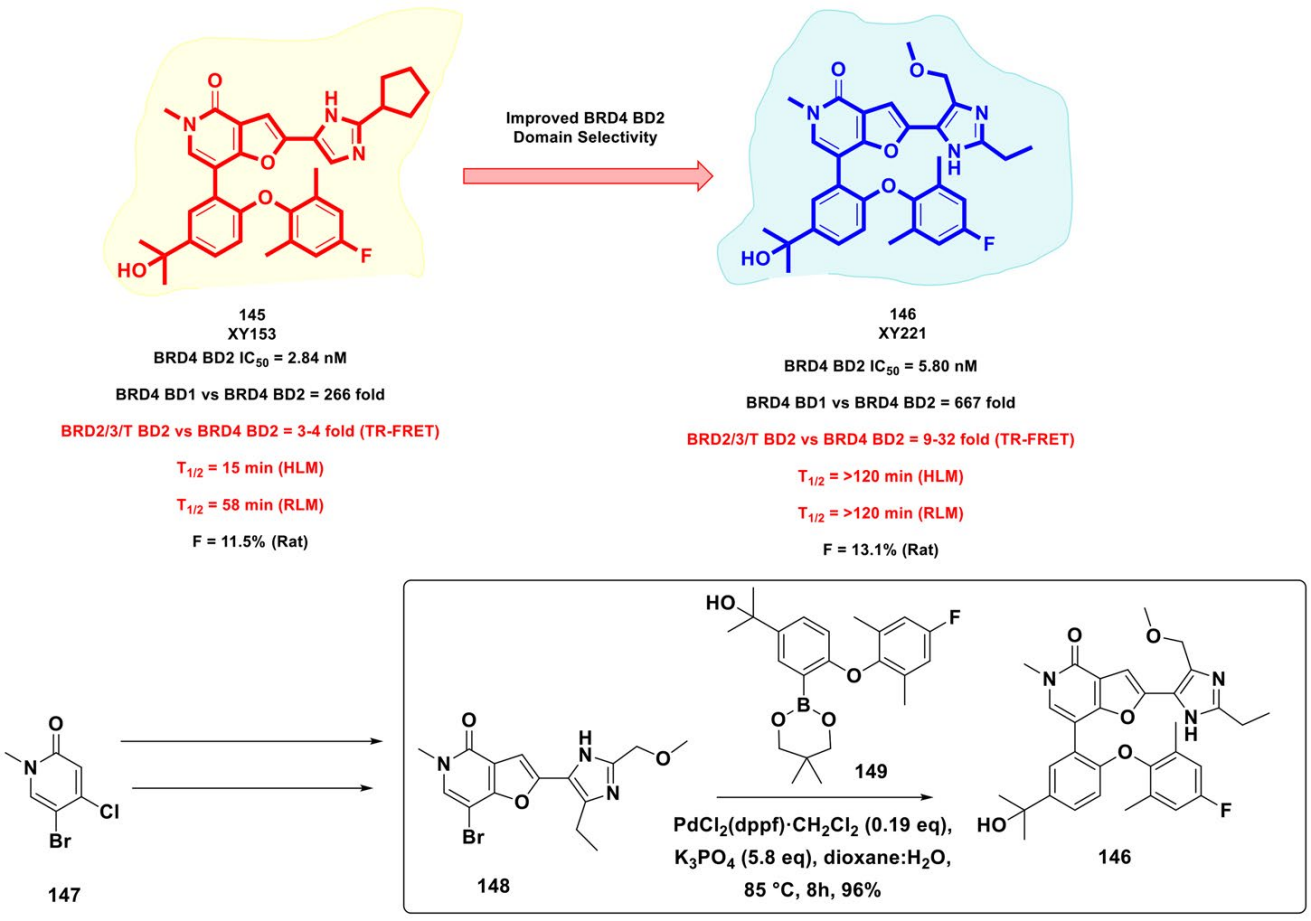
Design Strategy & Synthetic Route of First BRD4 Second Bromodomain (BD2)-Selective Inhibitors (the figure was drawn by the authors using chemdraw software).

Xu *et al*. reported the pioneering discovery of the first selective inhibitors specifically targeting BRD4’s second bromodomain (BD2) (Figure 27). Structural optimisation was inspired by the previously reported XY153 (**145**) pan BRD4 inhibitor. Research team successfully identified compound (**146**) (XY221) as a highly potent and selective BRD4 BD2 inhibitor (IC_50_ = 5.8 nM), exhibited 667-fold selectivity over BRD4 BD1 and 9–32-fold selectivity across other BET BD2 domains. Compound (**146**) effectively downregulates oncogenic proteins such as c-MYC, BCL-2, and MCL-1, inducing apoptosis in MV4-11 leukaemia cells. Compound (**146**) also demonstrated excellent liver microsomal stability (T_1/2_ > 120 min) and moderate oral bioavailability (F = 13.1%), supporting its potential as a selective BRD4 BD2-targeted therapeutic. The synthesis of compound (**146**) (**XY221**) was meticulously achieved through a strategic sequence of transformations that capitalised on the robustness of organopalladium-catalysed cross-coupling methodologies. Key steps involved the construction of a furo[3,2-*c*]pyridin-4(5H)-one core scaffold, which was subsequently functionalised via Suzuki-Miyaura coupling reactions between intermediate (**148**) and second coupling partner (**149**) employing PdCl_2_(dppf)·CH_2_Cl_2_ as the palladium catalyst to afford compound (**146**) with 96% yield. The utilisation of organopalladium catalysis was pivotal for the efficient formation of C–C bonds under relatively mild conditions, enabling high selectivity, excellent yields, and broad substrate scope^134^.

**Figure 28.**
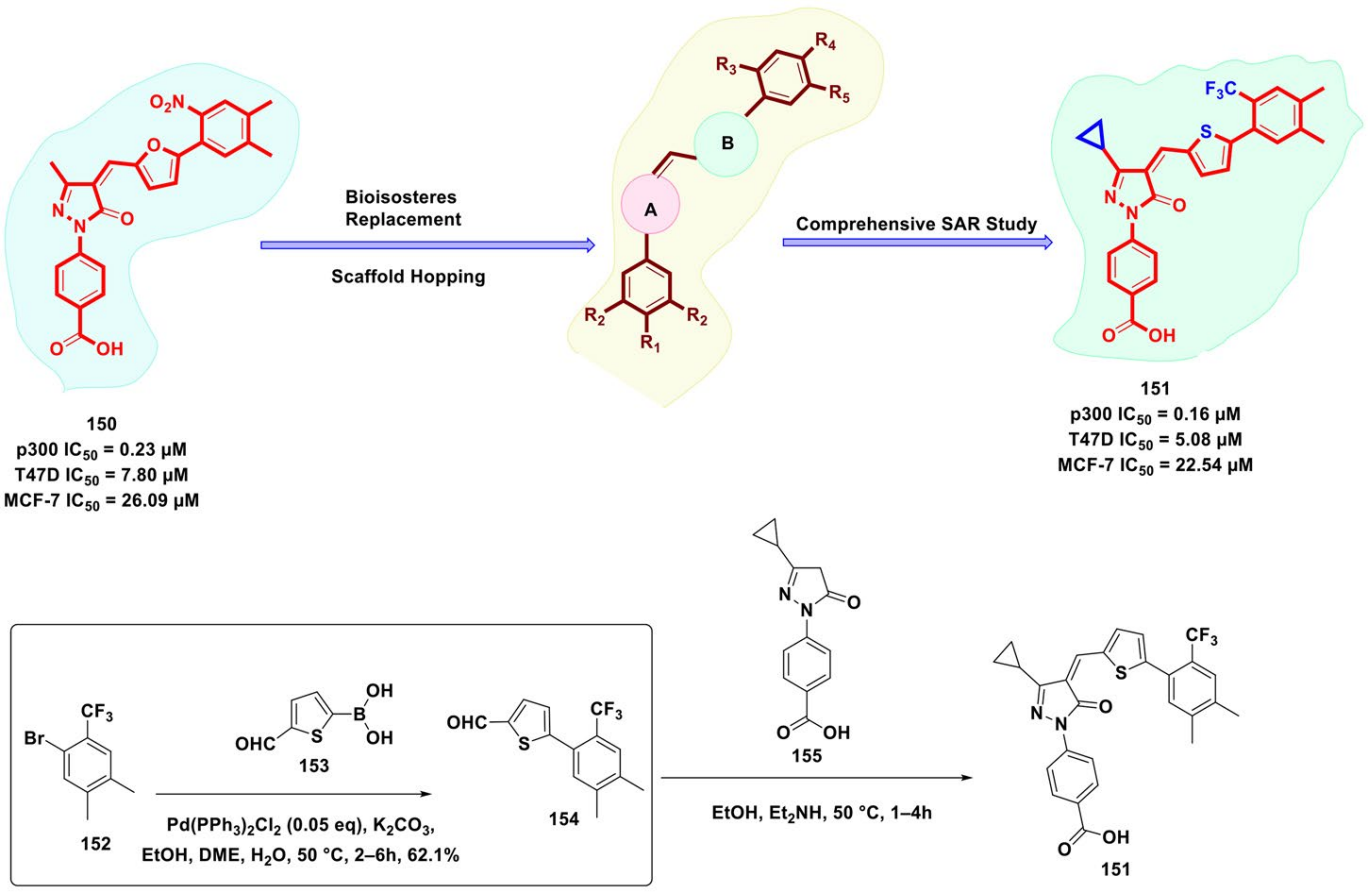
Design Strategy & Synthetic Route of a new class of histone acetyltransferase p300 inhibitors (the figure was drawn by the authors using chemdraw software).

Liu and colleagues launched a comprehensive medicinal chemistry campaign, employing strategies of bioisosteric replacement and rational scaffold hopping, to design, synthesise, and biologically evaluate a novel series of p300 inhibitors derived from the (**150**) C646 scaffold, as shown in Figure 28. A research team synthesised a library of 30 distinct compounds featuring diverse chemical moieties. In SAR determination, compound (**151**) emerged as a highly potent inhibitor of histone acetyltransferase p300, exhibiting an IC_50_ value of 0.16 μM, markedly superior to that of the standard inhibitor C646 (**150**), while also displaying enhanced drug-like properties. Western blot analysis demonstrated that compound **(151**) significantly reduced acetylation levels of histone H3 at lysine 27 (H3K27) more effectively than C646 (**150**). Furthermore, compound (**151**) exhibited robust antiproliferative effects against human breast cancer cell lines, with IC_50_ values of 5.08 μM in T47D cells and 22.54 μM in MCF7 cells. Molecular docking studies provided mechanistic insights into its superior inhibitory activity. The synthetic route of the potent compound commenced with an organopalladium mediated Pd(PPh_3_)_2_Cl_2_ Suzuki coupling reaction between 1-bromo-4,5-dimethyl-2-(trifluoromethyl)benzene (**152**) and (5-formylthiophen-2-yl)boronic acid (**153**) in the presence of Pd(PPh_3_)_2_Cl_2_ with potassium carbonate as base, producing key intermediate (**154**). Furthermore, *Knoevenagel condensation* was performed between intermediate (**154**) and intermediate (**155**) to yield desirable compound (**151**) by utilising diethylamine and ethanol heated at 50 °C for 1–4 h with 57.9% yield^135^.

**Figure 29.**
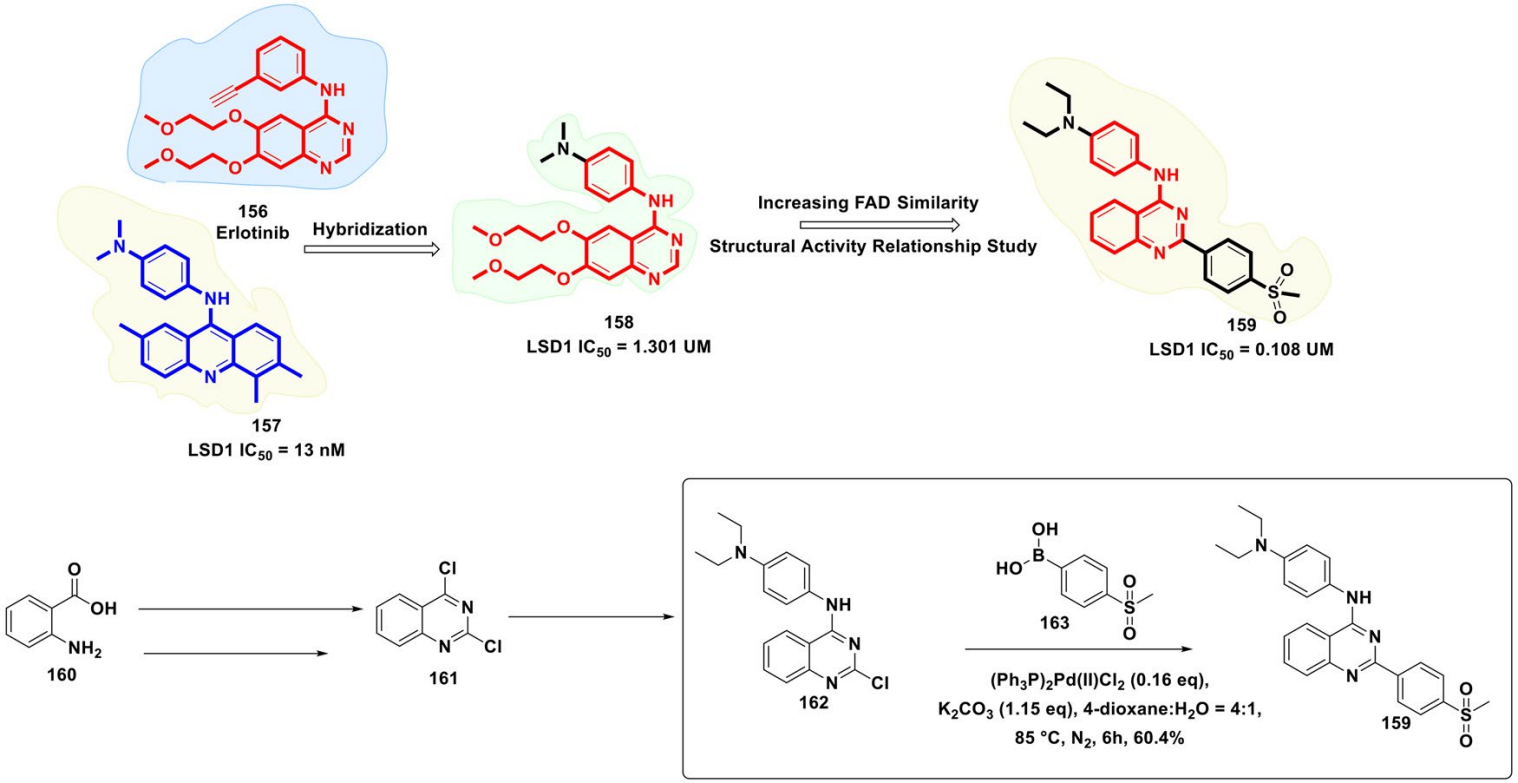
Design Strategy & Synthetic Route of 2‑Aryl-4-aminoquinazolin-Based LSD1 Inhibitors (the figure was drawn by the authors using chemdraw software).

Wang *et al.* designed and synthesised a series of 2-aryl-4-aminoquinazoline-based LSD1 inhibitors aimed at activating the immune response in gastric cancer, as shown in the Figure 29. The structural design strategy was inspired by a hybrid approach, combining features of Erlotinib (**156**), an EGFR tyrosine kinase inhibitor, and compound (**157**), a previously reported LSD1 inhibitor with favourable drug-like properties. A diverse library of quinazoline-based derivatives was developed and systematically evaluated for their LSD1 inhibitory potency, specifically targeting gastric cancer cells. Compound (**159**) demonstrated potent, selective inhibitory activity against LSD1 with an IC_50_ of 0.108 μM. Furthermore, compound (**159**) significantly downregulated PD-L1 expression in gastric cancer cells in both a time- and dose-dependent manner. *In vivo,* compound (**159**) significantly suppressed tumour growth in immunocompetent mice bearing MFC xenografts, with minimal systemic toxicity. It is important to highlight the role of organopalladium in the synthesis of the potent compound (**159**). The synthesis of compound (**159**) was accomplished through a convergent synthetic strategy and commenced with the intramolecular cycloaddition with 2-aminobenzoic acid (**160**), followed by chlorination, and nucleophilic aromatic substitution to yield intermediate (**162**), which was further subjected to organopalladium catalyst, mediated Suzuki coupling between boronic acid moiety (**163**) in the presence of dichlorobis(triphenylphosphine)palladium(II) as palladium catalyst and potassium carbonate as a base and solvent system used for the synthesis is dioxane: water in 4:1 at 85 °C for 6 h under inert conditions to generate the final desirable compound (**159**)^136^.

### Palladium Acetate-assisted synthesis of small molecule epigenetic inhibitors

One of the most widely used pre-catalysts in C-C bond formation reactions, palladium acetate has demonstrated utmost efficiency in smoothly catalysing the crucial steps of multistep synthetic routes to small molecule epigenetic inhibitors. Good solubility in organic solvents and efficient reduction to catalytically active Pd(0) species *in situ* define the success of palladium acetate as the catalyst for various cross-coupling reactions. This subsection highlights the contribution of this catalyst in expanding the size of the armoury of scaffolds addressing the epigenetic targets.

**Figure 30.**
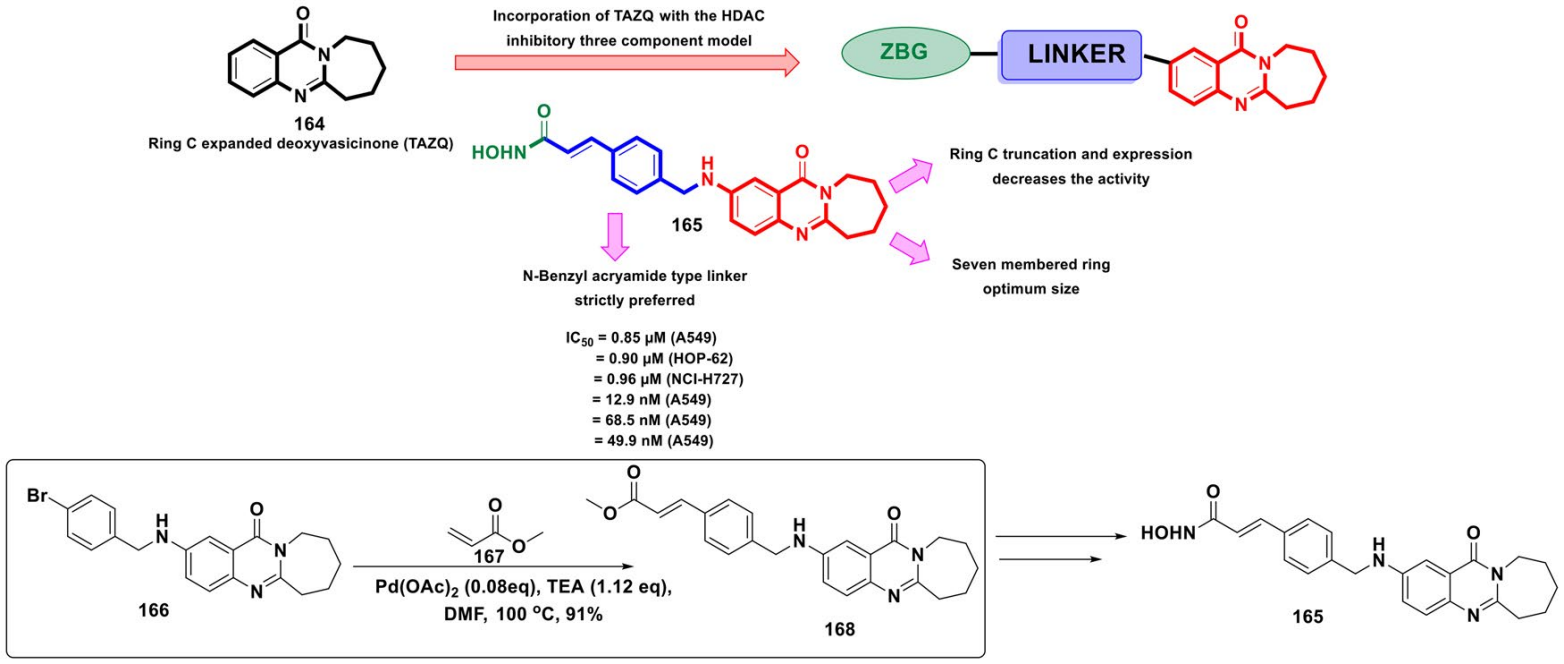
Design Strategy & Synthetic Route of Deoxyvasicinone-Based HDAC Inhibitor (the figure was drawn by the authors using chemdraw software).

Sharma *et al.,* as a part of their medicinal chemistry campaign to furnish anti-lung cancer agents, designed a series of prudently constructed HDAC inhibitors bearing a synthetically engineered alkaloid as the surface recognition part (Figure 30). Notably, an azepino quinazoline scaffold (**164**) with bronchidilatory action, structurally inspired by naturally occurring alkaloid Deoxyvasicinone, was accommodated in the structural template of designed adducts. The linker part to append the aforementioned structural motif with the zinc binding motif was comprehensively interrogated to furnish selective HDAC6 inhibitory scaffolds. Biological exploration of all the furnished adducts led the research group to pinpoint an HDAC inhibitor (**165**) featuring a benzyl acrylamide functionality that elicited magnificent anti-lung cancer activity. Noteworthy to mention that the aforementioned HDAC inhibitor (**165**) was generated via a multistep synthetic route, of which one of the steps involved the use of palladium acetate as the organopalladium catalyst for the generation of a key intermediate. The reaction basically involved the stitching of an acryl unit on the azepino quinazolone core via Heck olefination with methyl acrylate (**167**). Triethylamine was used as the base, and the reaction was performed in DMF at 100 °C for 2h. Encouragingly, the Heck olefination protocol yielded the intermediate (**168**) in excellent yields (91%). Noteworthy to mention that the azepino quinazolone (**168**) responded well to the reaction conditions, as the desired intermediate could be furnished without the use of a ligand. Thus, palladium acetate was found to be efficient in catalysing the Heck coupling in this case without the use of phosphine or any other strong ligand. Such a protocol is usually considered to be quite advantageous, as it enables the chemist to avoid air-sensitive phosphine ligands for which utmost precautions are required to be taken during the reaction. The intermediate (**168**) was further converted to the target adduct via a conventional route usually employed for the generation of hydroxamic acid-type HDAC inhibitors (**165**), *viz*., ester hydrolysis, amidation with NH_2_OTHP, and protic acid cleavage of tetrahydro pyranyl functionality. The target adduct (**165**) exhibited efficacies against KRAS and EGFR mutant lung cancer cell lines (IC_50_ = 0.80–0.96 μM) mediated via preferential HDAC6 inhibition, induced apoptosis, and increased autophagic flux. The compound (**165**) was found to be well accommodated within the active site of HDAC6 isoform, as indicated by the results of the docking studies that led the authors to figure out some key interactions with the amino acid residues of HDAC6. Further, the hydroxamic acid (**165**) was leveraged for the generation of pH-responsive nanocarrier (Hyaluronic acid-fused quinazolinone (**165**) nanoparticles) that demonstrated pH-sensitive antitumor effects, thereby exerting selective cell viability reduction potential towards the lung cancer cell lines^137^.

**Figure 31.**
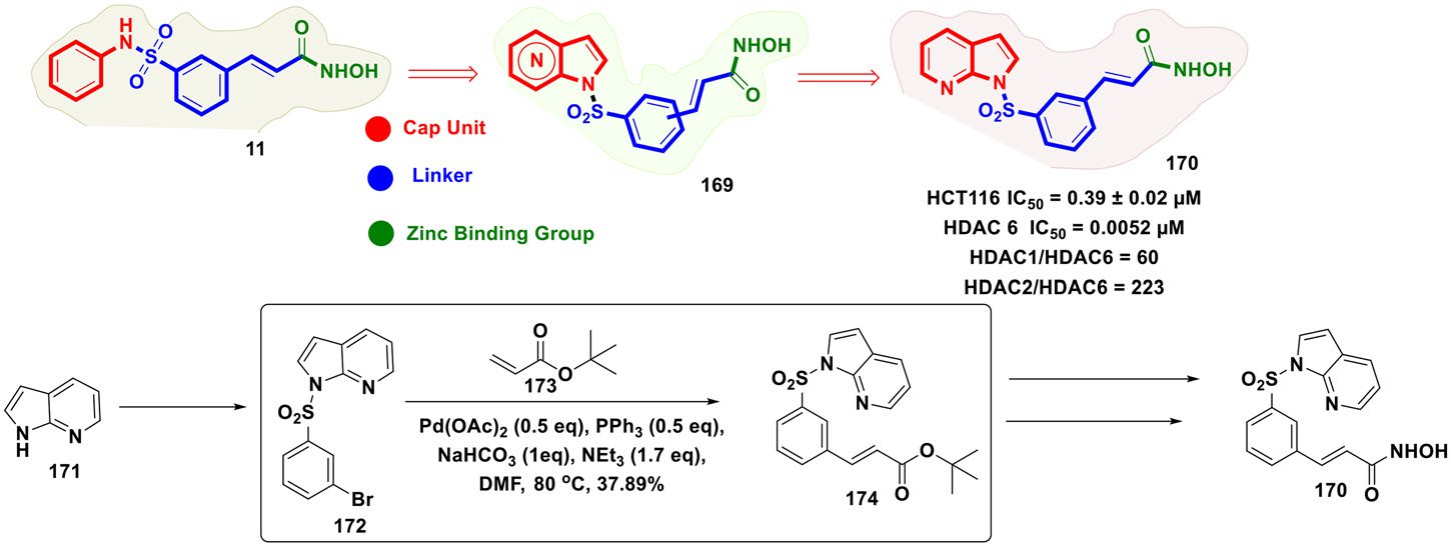
Design Strategy & Synthetic Route of Azaindolylsulfonamides Based Histone Deacetylase 6 Activity (the figure was drawn by the authors using chemdraw software).

Lee *et al.* designed and synthesised a series of selective HDAC6 inhibitors as anticolorectal agents (Figure 31). To ensure the selectivity of the resulting scaffolds towards the HDAC6 isoform, the research group exercised a comprehensive investigation of the linker part of the HDAC inhibitor pharmacophore. The correlation of the cellular and enzymatic data revealed that an acrylamide-stitched hydroxamic acid elicited remarkable *in vitro* and *in vivo* anticolorectal effects mediated via selective HDAC6 inhibition. Noteworthy to mention that the scaffold demonstrated 60-fold and 223-fold higher selectivity towards HDAC6 in comparison to HDAC1 and HDAC2, respectively. This selectivity towards the HDAC6 isoform was assumed to be instrumental in conferring magnificent anti-colorectal efficacy to the resulting adducts. A structural analysis of all the scaffolds furnished in this study highlights that the templates only varied in the linker part, as the surface recognition part (Aza indoles) and the zinc binding motif (hydroxamic acid) were the constant structural features of the scaffolds. Thus, the selective HDAC6 inhibitory trends were attributed to the existence of specific linkers (acrylamide) in the chemical architecture of the accomplished scaffolds. Notably, to install an acrylamide linker, the research group utilised an organopalladium-catalysed Heck coupling reaction as an important reaction of their multi-step synthetic route to the desired compound. Specifically, C-C bond formation was accomplished between tert-butyl acrylate and 1-(3-bromophenyl)sulfonyl)-1(H)-pyrrolo[2,3-*b*]pyridine (**172**) using palladium acetate as the catalyst and triphenyl phosphine as the ligand. Triethylamine was used as a base for the reaction, and the reaction was performed in DMF at 80 °C for 5h. The yield of the reaction was low (37.89%). The intermediate (**174**) was further converted to the target hydroxamic acid via a conventional reaction methodology. Gratifyingly, the compound demonstrated an impressive pharmacokinetic profile with oral bioavailability of 33% as well as *in vivo* pharmacodynamics effect as evident by tumour growth inhibitory activity of (**170**) in colorectal HCT116 xenografts^138^.

**Figure 32.**
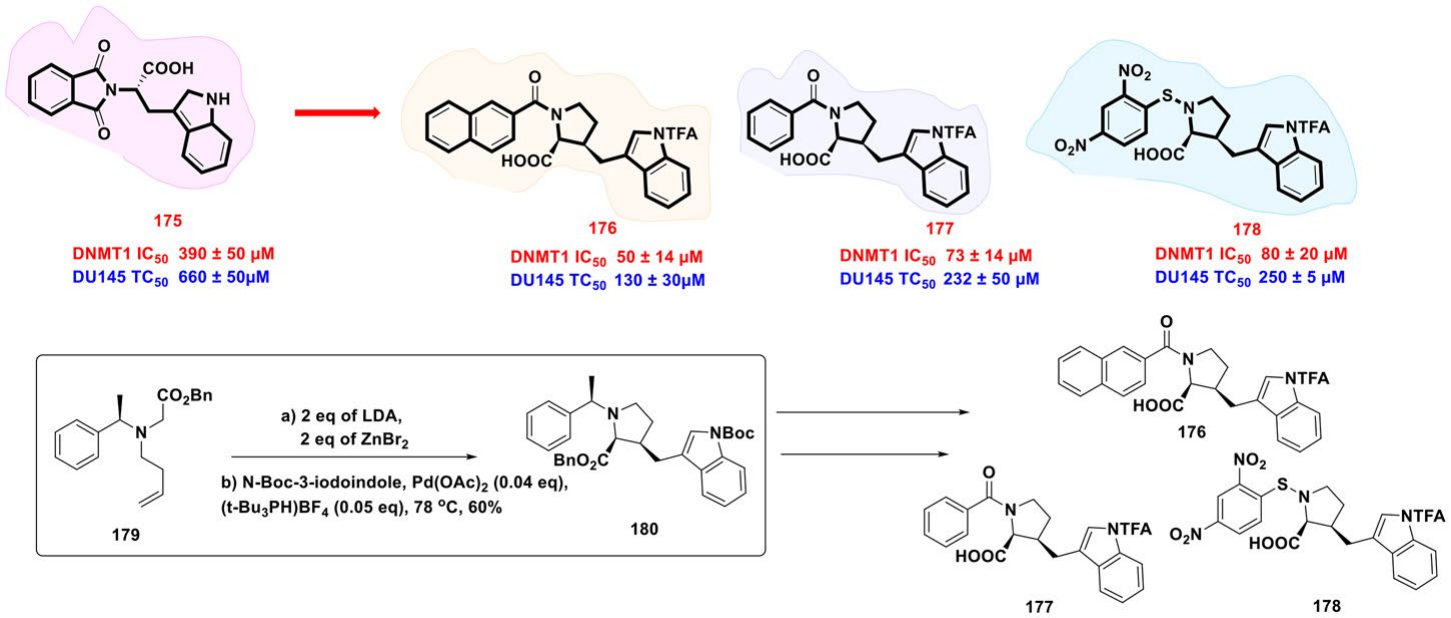
Design Strategy & Synthetic Route of N‑Phthaloyl‑L‑tryptophan Based DNA Methyltransferase 1 Inhibitors (the figure was drawn by the authors using chemdraw software).

Asgatay *et al.* employed the applications of Negishi coupling for catalysing the crucial step of the multistep synthetic routes to DNA methyltransferase inhibitors (Figure 32). The synthetic pathway commenced with the carbo cyclisation of olefin (**179**) via deprotonation using LDA and transmetallation employing ZnBr_2,_ followed by a subsequent Negishi cross-coupling reaction between N-Boc-3-iodoindole and cyclic zinc intermediate. Notably, the Negishi coupling reaction was accomplished using [Pd(OAc)_2_] as an organopalladium catalyst. Tri-tert-butyl phosphine tetrafluoroborate was commonly used as a ligand source. Notably, (*t*-Bu_3_PH)BF_4_ is often a ligand of choice for this coupling as the BF4^-^ salt makes the ligand more stable. Moreover, t-Bu_3_P is electron-rich and enhances the reactivity of the palladium catalyst. The reaction was carried out in dry THF and was conducted under argon overnight to afford the formation of the intermediate (**161**) in acceptable yields. The intermediate (**180**) was further leveraged for the construction of the target adducts (**176–178**) that manifested a striking ability to inhibit DNMT1 and exert potent cell growth inhibitory effects against the human prostate carcinoma cell line (DU145 cell line)^139^.

**Figure 33.**
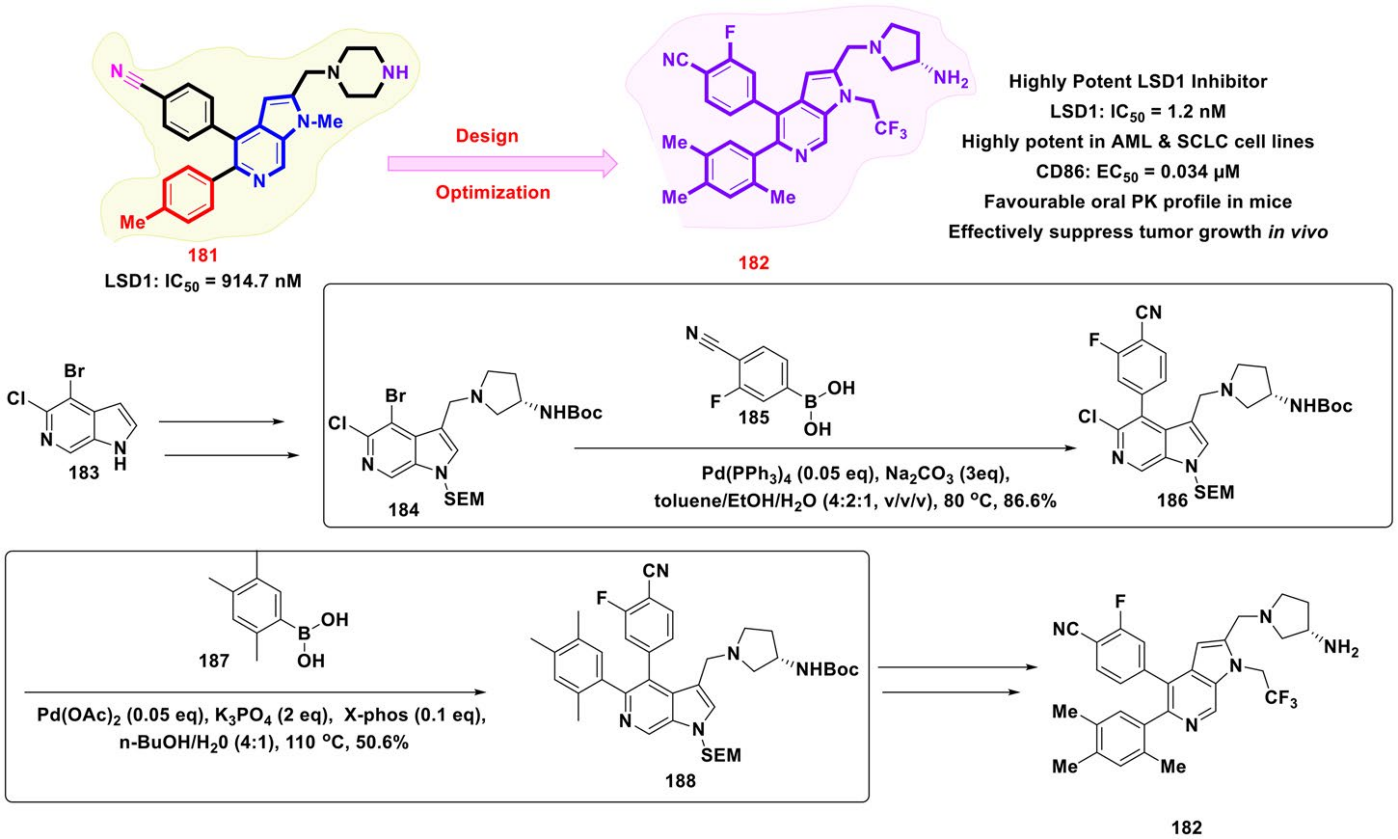
Design Strategy & Synthetic Route of Novel 1H‑Pyrrolo[2,3‑c]pyridin Derivatives Based Lysine Specific Demethylase 1 Inhibitors (the figure was drawn by the authors using chemdraw software).

Jiang *et al.* designed and synthesised 1(H*)*-pyrrolo[2,3-*c*] pyridine-based LSD1 inhibitors as anti-leukaemia agents utilising compound (**181**) as the lead structural template (Figure 33). The outcome of the biological evaluations culminated in the identification of a selective and reversible LSD1 inhibitor (**182**) endowed with remarkable cell growth inhibitory effects against AML cell lines (MV4 − 11 and Kasumi-1). Further evaluation results revealed that i) compound (**182**) induced differentiation of AML cell lines, ii) compound (**182**) demonstrated a favourable oral PK profile iii) compound (**163**) exerted tumour growth suppression in an AML xenograft model. Notably, the synthetic route to compound (**182**) utilised the applications of two consecutive Suzuki cross-coupling reactions catalysed by organopalladiums. The first Suzuki coupling between tert-butyl (S)-(1-((4-bromo-5-chloro-1-((2-(trimethylsilyl)ethoxy)methyl)-1(H)-pyrrolo[2,3-*c*]pyridin-3-yl)methyl)pyrrolidin-3-yl)carbamate (**184**) and (4-cyano-3-fluorophenyl)boronic acid (**185**) was catalysed by Pd(PPh_3_)_4_ to generate intermediate (**186**). Sodium carbonate was used as the base in the reaction. The reaction was carried out in a mixture of toluene: ethanol: water (4:2:1) at 80 °C with a yield of 86.6%. The second Suzuki cross coupling reaction between tert-butyl (*S*)-(1-((5-chloro-4-(3-cyano-4-fluorophenyl)-1-((2-(trimethylsilyl)ethoxy)methyl)-1(H)-pyrrolo[2,3-*c*]pyridin-3-yl)methyl)pyrrolidin-3-yl)carbamate (**186**) and (3,4,5-trimethylphenyl)boronic acid (**187**) was catalysed by palladium acetate to generate intermediate (**188**). An electronically rich and sterically bulky biaryl phosphine ligand was used to enhance the reactivity and the stability of the palladium catalysts. Potassium phosphate was used as the base, and the reaction was carried out in a mixture of *n*-butanol and water (4:1) at 110 °C. The intermediate (**188**) was converted to the target compound (**182**) via classical organic chemistry methodologies^140^.

**Figure 34.**
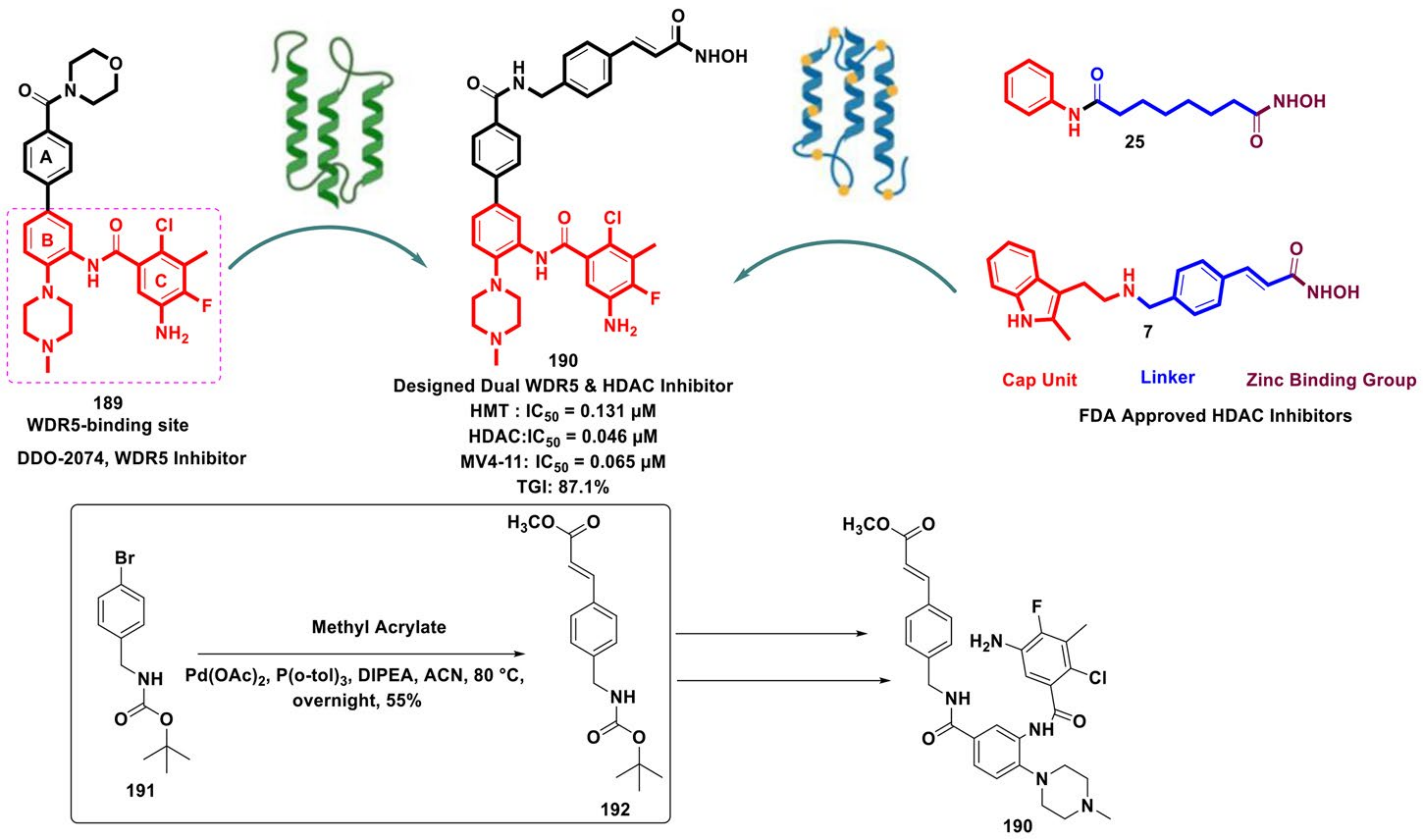
Design Strategy & Synthetic Route of Dual WDR5 − MLL1 and HDAC Inhibitor (the figure was drawn by the authors using chemdraw software).

Long and his research group conducted a preliminary experiment that indicated synergistic effects of the cocktail of WDR5 and HDAC inhibitors owing to dynamic modulation of H3 acetylation and H3K4 methylation. On the basis of the aforementioned preliminary results, MLL1 − WDR5/HDAC dual-target inhibitors were designed and synthesised by the group (Figure 34). Notably, the structural template of the designed dual adducts featured the pharmacophores of DDO-2074 and vorinostat. The outcome of this study culminated in the identification of a strikingly potent dual WDR5 − MLL1 and HDAC inhibitor (**190**). The synthetic route to the target scaffold commenced with the Heck coupling of methyl acrylate and tert-butyl (4-bromobenzyl)carbamate (**191**) to afford the formation of a key intermediate (**192**) that was further used for appendage with the DDO pharmacophore via PyBOP-assisted amidation procedure. The Heck olefination in this study was catalysed by palladium acetate, and tri(o-tolyl)phosphine was employed as a ligand for the palladium catalyst. The reason for using tri(o-tolyl)phosphine over triphenyl phosphine could be the stronger sigma donor ability to increase the electron density on the palladium centre, thereby facilitating the oxidative addition of aryl halides to Pd(0). Acetonitrile was used as the solvent, and the reaction was conducted at 80 °C overnight. The desired intermediate (**192**) was obtained in acceptable yields and thus was leveraged for further reactions to assemble the target adduct. Impressively, compound (**190**) exerted significant antiproliferative effects via impediment of the DNA damage repair signalling pathway. Compound (**190**) also demonstrated significant *in vivo* antitumor efficacy in the MV-4-11 xenograft model at the dose of 30 mg/kg (TGI = 87.1%)^141^.

**Figure 35.**
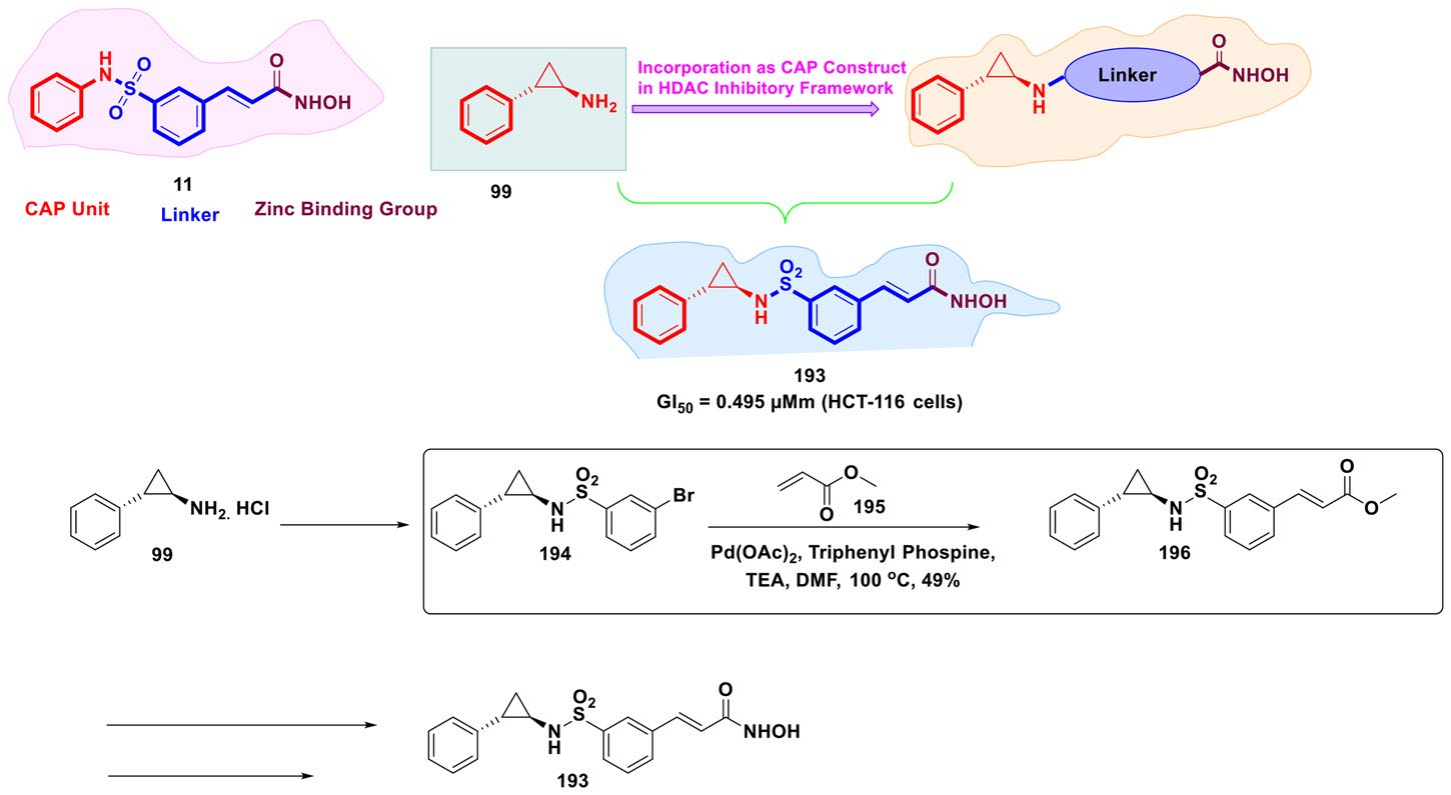
Design Strategy & Synthetic Route of Dual LSD1 and HDAC Inhibitor (the figure was drawn by the authors using chemdraw software).

Chou *et al.* developed dual LSD1–HDAC inhibitors based on the tranylcypromine scaffold as surface recognition part, integrating hydroxamic acid moiety as zinc-binding motif, and further, systematically optimising linkers to achieve HDAC6 selectivity. Given HDAC6’s therapeutic relevance in cancer, particularly colorectal cancer, diverse linkers were synthesised and evaluated, which yielded a series of rationally designed dual inhibitors with promising *in vitro* and *in vivo* antitumor activity. The results of the biological evaluations culminated in the identification of a potent scaffold (**193**) that was endowed with striking anticolorectal activity. It is important to mention that the chemical structures furnished in this study have a major structural overlap with each other and differ only in the context of the linker component. Scaffold (**193**), comprised of an acrylamide-type linker and Heck olefination catalysed by palladium acetate, was used for the insertion of the acrylamide linker. Triphenyl phosphine was used as a ligand in the reaction to form Pd(0)-PPh_3_ complex, thereby facilitating the insertion of the acryl moiety. Triethylamine was used as the base, and the reaction was conducted in DMF at 100 °C. The resulting intermediate (**196**) was converted to the target hydroxamic acid via conventional methodology. Gratifyingly, the compound manifested significant cell growth inhibition (GI_50_ = 0.495 *μ*M) against colorectal cell lines which might be exerted via selective HDAC6 and LSD1 inhibition. Moreover, compound (**193**) exhibited prominent antitumor activity in the *in vivo* studies (Xenograft model) and also effectively inhibited growth in patient-derived CRC organoids, as shown in Figure 35^142^.

### Pd_2_(dba)_3_ and Pd(dba)_2_ catalysis

Pd_2_(dba)_3_ and Pd(dba)_2_ are Pd(0) sources with labile dibenzylideneacetone (dba) ligands, making them highly reactive and versatile for reactions like Heck, Suzuki, and Buchwald-Hartwig couplings. The reactivity of Pd_2_(dba)_3_ and Pd(dba)_2_ depends on the electronic properties of the dba ligands. When the dba ligand has more electron-donating groups (such as methoxy), it weakens the bond between palladium and dba, making more active Pd(0) species available for oxidative addition and increasing the catalytic activity. In contrast, electron-withdrawing groups (like CF_3_) strengthen the Pd–dba bond, stabilising the complex but reducing reactivity^31–36^. This section covers Pd_2_(dba)_3_ and Pd(dba)_2_ assisted furnishment of small molecule inhibitors of the epigenetic targets.

**Figure 36.**
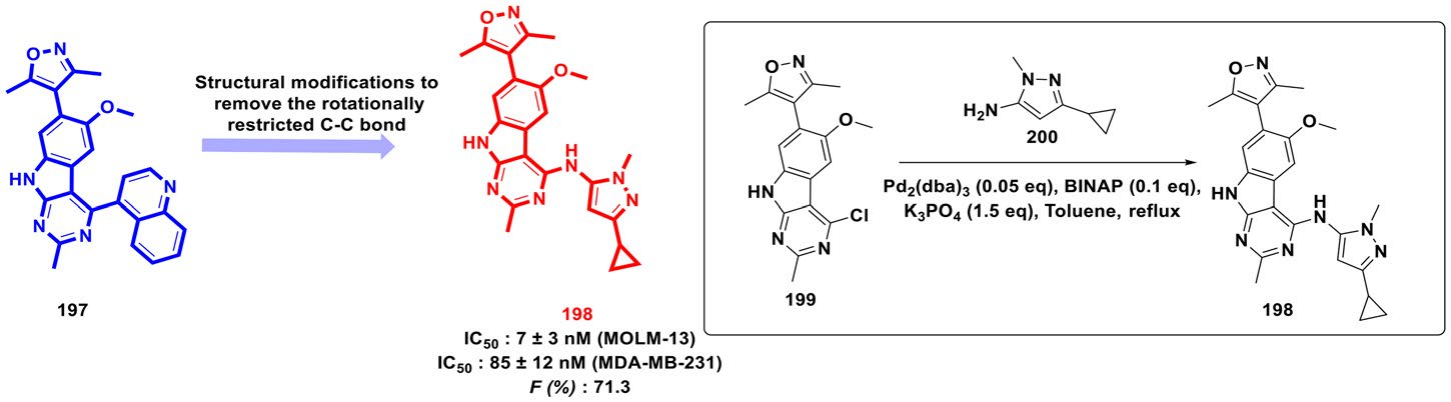
Design Strategy & Synthetic Route of Discovery of CF53 as BET Inhibitor (the figure was drawn by the authors using chemdraw software).

Wang *et al.* reported a structure-guided approach which led to the identification of CF53 (**198**), a highly potent and orally bioavailable BET inhibitor (Figure 36). Structural optimisation through the incorporation of an NH-substituted pyrazole moiety into the 9(H)-pyrimido[4,5-*b*]indole scaffold (**199**) yielded a series of compounds exhibiting sub-nanomolar binding affinities (Ki < 1 nM) for the BRD4 BD1 domain and exhibited low-nanomolar potency in inhibiting the proliferation of leukaemia and breast cancer cells. Notably, CF53 (**198**) exhibited remarkable selectivity for BET proteins over other bromodomain-containing proteins. Previous BET inhibitors exhibited restricted C–C bond rotation, which contributed to their limited therapeutic efficacy. To overcome this structural constraint, the group strategically modified compound (**197**) to eliminate the rotationally restricted C–C bond, thereby enhancing its pharmacological properties. The synthesis of CF53 (**198**) predominantly relies on organopalladium-catalysed C–N bond formation, as depicted in Figure 35. The key intermediate (**199**) was subjected to Buchwald–Hartwig coupling with 3-cyclopropyl-1-methyl-1H-pyrazol-5-amine (**200**) as the primary amine coupling partner. This transformation was facilitated by Pd_2_(dba)_3_ as the palladium catalyst, while BINAP served as a chiral ligand to stabilise the active palladium complex. Tripotassium phosphate was employed as the base, and the reaction was conducted in toluene at 100 °C under anhydrous conditions for an extended duration (overnight), affording CF53 (**198**) in high yield^143^.

**Figure 37.**
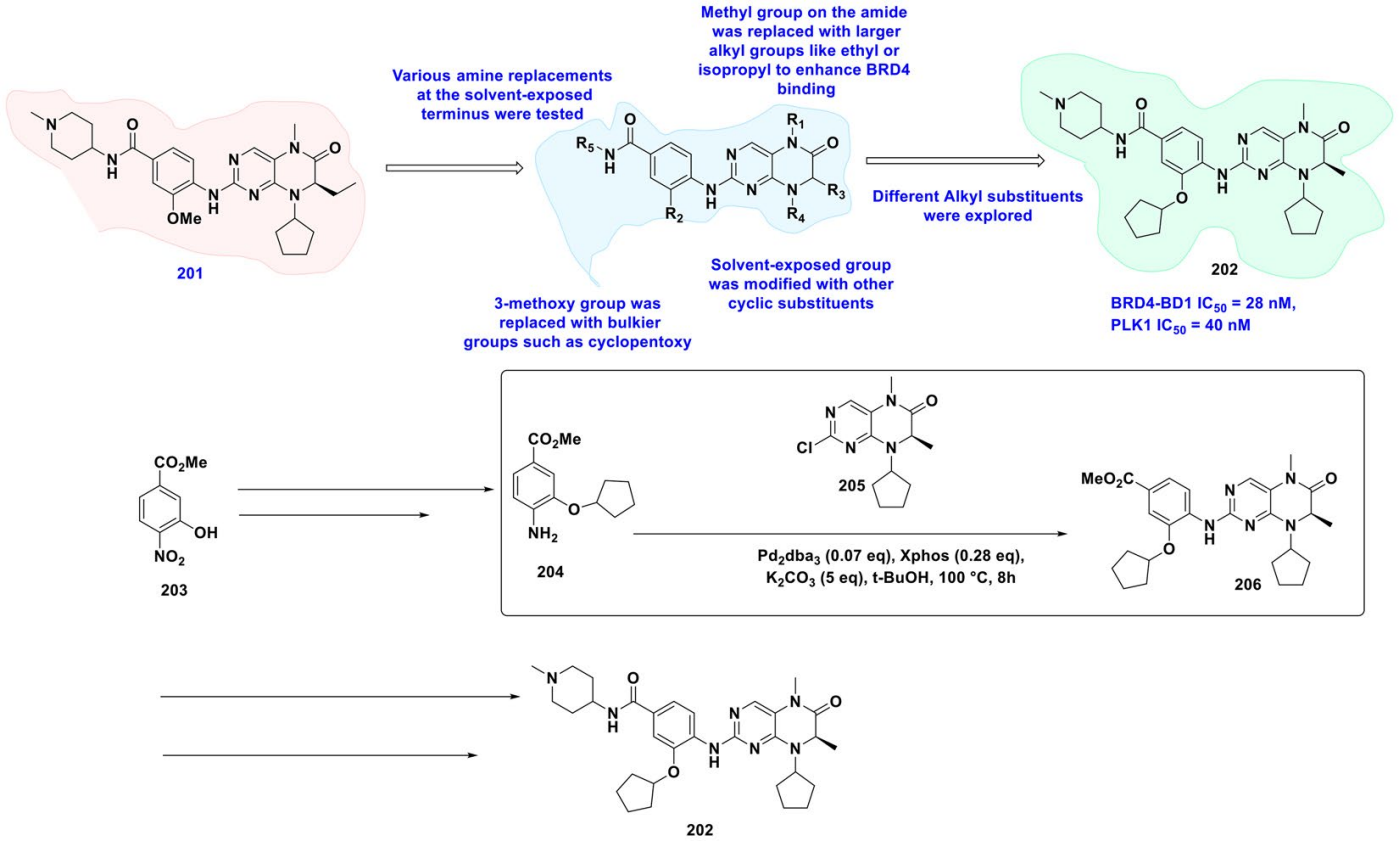
Design Strategy & Synthetic Route of Potent and Selective Dual BRD4/Polo-like Kinase 1 (PLK1) Inhibitors (the figure was drawn by the authors using chemdraw software).

To develop potent inhibitors targeting both BRD4 and PLK1, Wei Zhang and his team adopted a dual inhibition strategy to design and synthesise a series of potent and selective dual BRD4/PLK1 inhibitors (Figure 37). A medicinal chemistry campaign was commenced to systematically modify the BI-2536 scaffold (**201**), leading to the development of structurally diverse small molecules capable of dual inhibition of BRD4 and PLK1. Among the synthesised compounds, compound (**202**) emerged as a highly potent dual kinase-bromodomain inhibitor, exhibiting an IC_50_ value of 28 nM for BRD4-BD1 and 40 nM for PLK1. Notably, compound (**202**) demonstrated exceptional selectivity for PLK1 over BRD4, with IC_50_ values of 9.9 nM for PLK1 and 2579 nM for BRD4-BD1, highlighting its preferential kinase-targeting properties. The synthesis of compound (**202**) was predominantly reliant on organopalladium-catalysed transformations. The synthetic route started with the alkylation and reduction to afford the amine. The resulting amine (**204**) intermediate then undergoes a palladium-catalysed cross-coupling reaction with an aryl chloride in the presence of X-Phos as the ligand, Pd_2_(dba)_3_ as the palladium catalyst, and K_2_CO_3_ as the base in tert-butanol at 100 °C for 8h, yielding intermediate around 50% (**206**). The reaction mixture subsequently undergoes ester hydrolysis and amide coupling reaction to afford the final compound (**202**). This optimised synthetic route proficiently includes key organopalladium-mediated transformations to construct the desired molecular architecture^144^.

**Figure 38.**
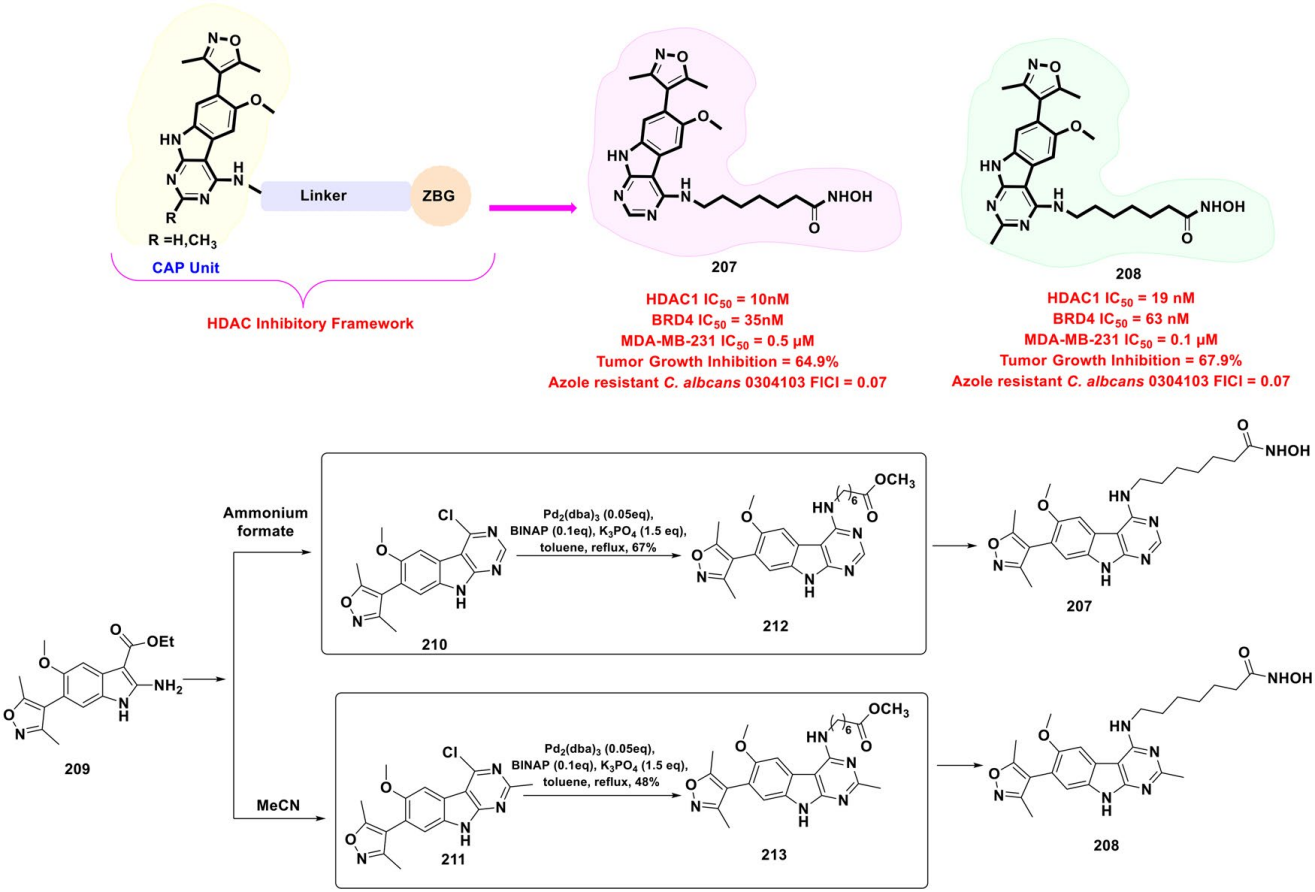
Design Strategy & Synthetic Route of Dual BET & HDAC Inhibitor (the figure was drawn by the authors using chemdraw software).

Therapeutic interventions for breast cancer often inadvertently promote *Candida albicans* infections, which in turn can exacerbate tumour progression and metastatic spread. Existing therapies often fail to address both conditions simultaneously through drug resistance. Accumulating evidence from previous studies indicates that HDAC and BET inhibitors possess dual antifungal and antineoplastic properties. To exploit this mechanistic synergy, Huang *et al.* designed and synthesised BET-HDAC dual inhibitors to achieve synergistic epigenetic modulation (Figure 38). The synthetic route of the final hydroxamic acid compounds commenced with the cyclocondensation of commercially available ethyl 2-amino-6-(3,5-dimethylisoxazol-4-yl)-5-methoxy-1H-indole-3-carboxylate (**209**), then the chlorination reaction yielded intermediates (**210**) and (**211**). Subsequent palladium-catalysed coupling of these intermediates with a range of amides using Pd_2_(dba)_3_ as the catalyst & BINAP as the ligand, K_3_PO_4_ as the base, and toluene as the solvent at 140 °C yielded the corresponding intermediates (**212**) and (**213**). These intermediates were then treated with a methanolic solution of hydroxylamine to furnish the desired hydroxamic acid derivatives. The synthesised compounds exhibited robust dual-target inhibitory efficacy, effectively suppressing MYC expression, promoting histone hyperacetylation, and initiating apoptotic cascades in malignant cell lines. Notably, compounds (**207**) and (**208**) displayed exceptional potency in attenuating breast cancer progression while concurrently combating *Candida albicans* infections, achieving nanomolar-scale inhibition against both BRD4 (**207**: IC_50_ = 63 nM; **208**: IC_50_ = 35 nM) and HDAC1 (**207**: IC_50_ = 10 nM; **208**: IC_50_ = 19 nM), surpassing the efficacy of conventional single-target inhibitors. Both compounds exhibited exceptional anticancer and antifungal activities both *in vitro* and *in vivo*^145^.

**Figure 39.**
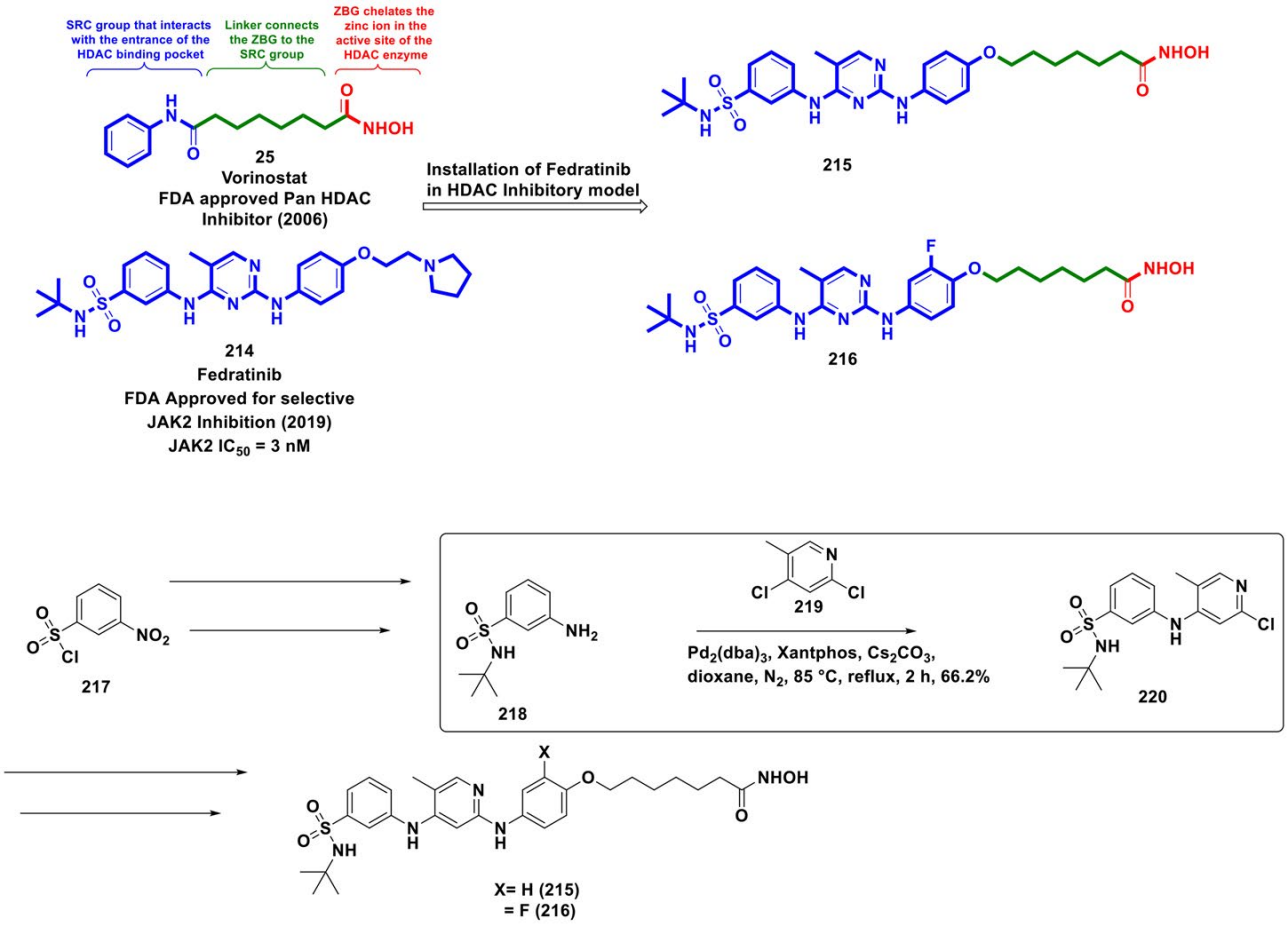
Design Strategy & Synthetic Route of Fedratinib-Based JAK and HDAC Bispecific Inhibitors (the figure was drawn by the authors using chemdraw software).

Qian *et al*. developed a new class of dual JAK and HDAC inhibitors, using Fedratinib (**214**) as the central scaffold (Figure 39). The goal was to target both haematologic cancers and solid tumours by leveraging the known synergistic effects of JAK and HDAC blockade. HDAC-inhibitory pharmacophores were utilised for this study, in which Fedratinib acts as the capping group. Various linkers were used to connect this cap to a hydroxamic acid-based ZBD, a well-established motif for strong HDAC inhibition. To synthesise the novel dual inhibitors, organopalladium-mediated synthetic methods were employed. A Buchwald–Hartwig amination was carried out using Pd_2_(dba)_3_, a pre-activated Pd(0) catalyst, with caesium carbonate as the base at 85 °C for 2 h, yielding the key intermediate (**220**) in 66.2%. Pd_2_(dba)_3_ is highly effective in such couplings due to its inherent Pd(0) state, eliminating the need for external reductants. Intermediate (**220**) then underwent nucleophilic substitution to form an ester intermediate, which was subsequently converted to the corresponding hydroxamic acids under alkaline NH_2_OK conditions. Compounds (**215**) and (**216**) emerged as the lead candidates, showing strong dual inhibition of JAK1/2 and HDAC isoforms, particularly HDAC3 and HDAC6, with notable selectivity towards JAK2. Structurally, analogs containing a six-carbon linker attached to an aromatic ring, either unsubstituted or fluorinated benzene, displayed significant antiproliferative and proapoptotic activity *in vitro*. Mechanistic studies indicated that these effects were driven by modulation of the JAK-STAT signalling pathway and HDAC-related epigenetic regulation. *In vivo*, both compounds induced potent synergistic antitumor effects in HEL (haematologic) and A549 (solid tumour) xenograft models, without causing systemic toxicity. Overall, the results highlight dual JAK/HDAC inhibition as a promising multimodal cancer therapy that combines targeted pathway blockade with epigenetic reprogramming^146^.

**Figure 40.**
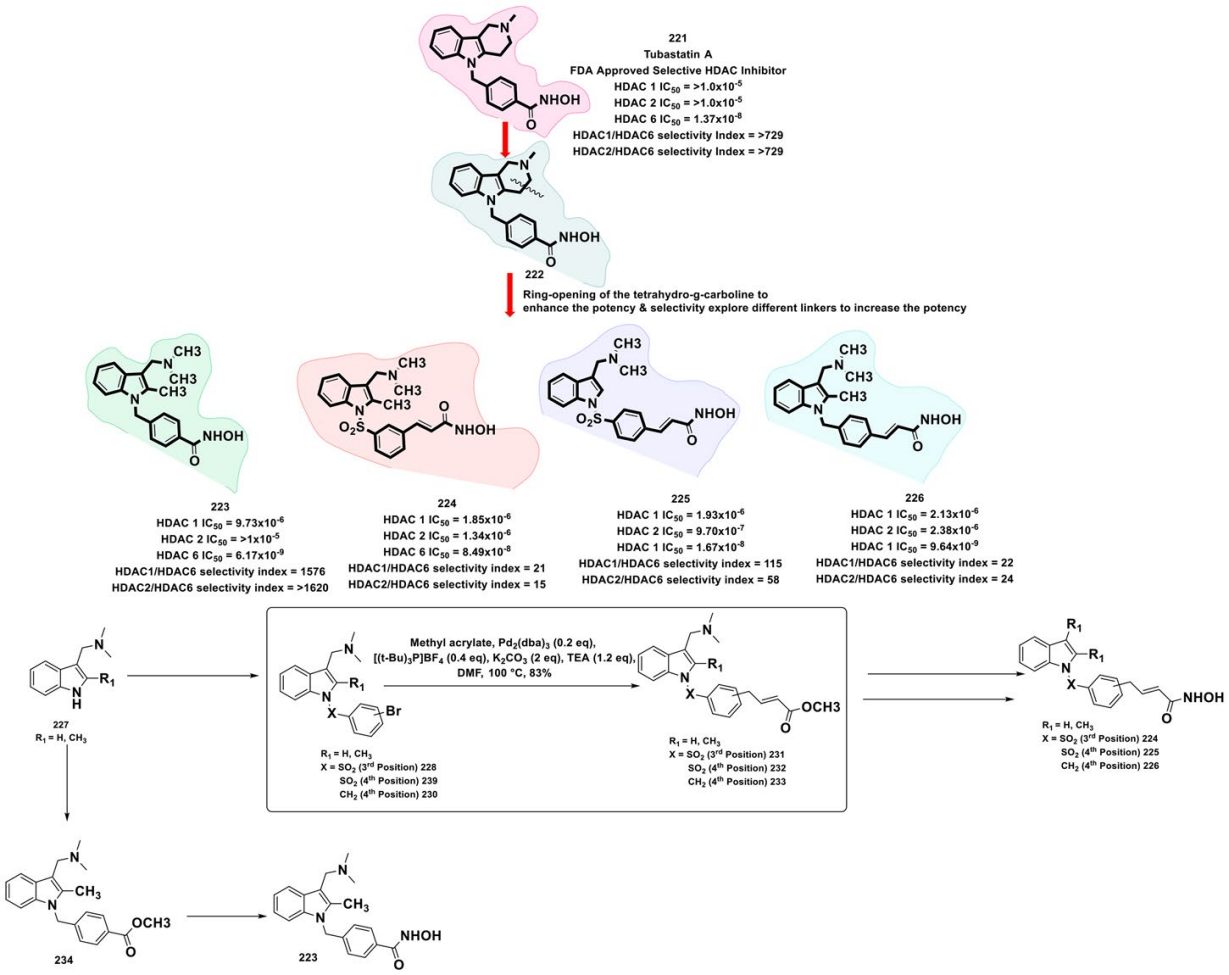
Design Strategy & Synthetic Route of Tubastatin A-Based HDAC Inhibitors (the figure was drawn by the authors using chemdraw software).

Earlier investigations demonstrated that organopalladium catalysis plays a significant role in improving the pharmacokinetic and pharmacodynamic properties of anticancer scaffolds. On the building of this context, Nepali *et al.* commenced a medicinal chemistry campaign to rationally design and synthesise novel HDAC6 inhibitors by performing structural modifications on FDA-approved HDAC6 inhibitor tubastatin A (**221**). The synthetic route of these compounds (as shown in Figure 40**)** relied on modern organic chemistry techniques, which initiated with a Heck coupling reaction catalysed by the organopalladium complex Pd_2_(dba)_3_ to produce key ester intermediates (**231–233**). Pd_2_(dba)_3_ offers notable benefits, including high thermal stability and excellent efficiency in facilitating Heck couplings. The use of specialised ligands, such as tri-tert-butylphosphonium tetrafluoroborate, demonstrated a ligand-accelerated catalytic process. These sterically demanding phosphine ligands enhance palladium centre stability and improve catalytic turnover, while also minimising side reactions and improving reaction kinetics. The ester intermediates (**231–233**) were subsequently converted to carboxylic acids using lithium oxide monohydrate. These acids then undergo amidation with NH_2_OTHP, followed by TFA-mediated deprotection, to yield the target hydroxamic acids in good overall yield. Structure-activity relationship analysis revealed that compounds featuring a benzyl motif (**223**) retained HDAC6 selectivity (IC_50_ in the low nM range) but exhibited negligible antiproliferative activity. In contrast, compounds incorporating N-sulfonyl/benzyl acrylamide linkers (**224–226**) exhibited dual functionality: robust HDAC6 inhibition, confirmed by hyperacetylation of tubulin, and potent cytotoxic effects. These optimised analogs demonstrated superior efficacy to tubastatin A (**221**), suppressing colony formation in a dose-dependent manner and inducing apoptosis via PARP cleavage and caspase-3 activation^147^.

**Figure 41.**
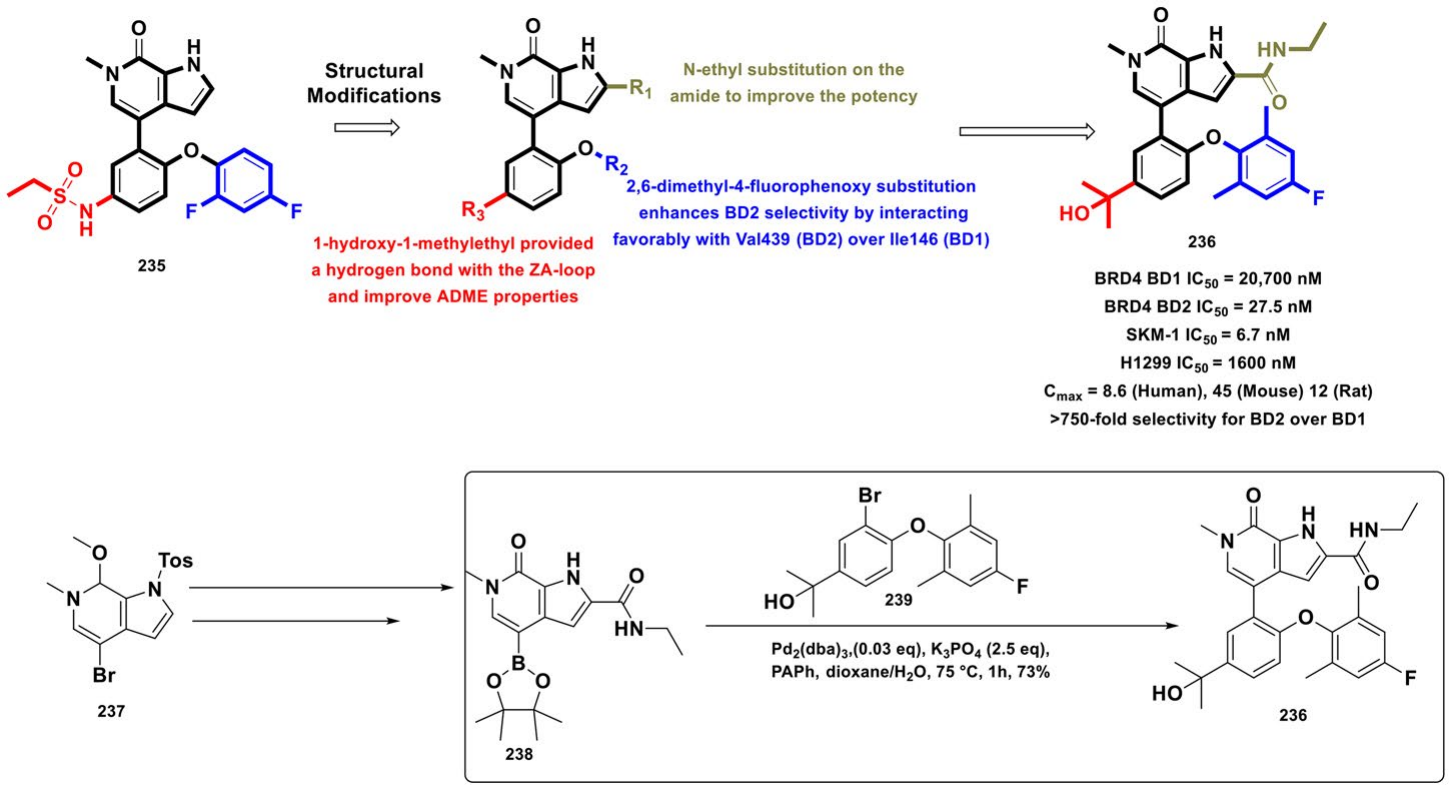
Design Strategy & Synthetic Route of BET Bromodomain Inhibitor (the figure was drawn by the authors using chemdraw software).

Sheppard *et al.* designed pyrrolopyridone-based chemotypes selective BD2 inhibitors through rational structural optimisation of the dual-bromodomain clinical compound ABBV-075 (**235**). These efforts yielded compound (**236**), a potent and selective BD2 inhibitor with 6.7 nM activity in SKM-1 leukemic cells, 1,600 nM in H1299 carcinoma cells, and a 330-fold selectivity index for BD2 over BD1 domains. The synthetic route to (**236**) employed sequential organopalladium-catalysed transformations commencing with Miyaura borylation of an aryl halide precursor which was achieved using an XPhos-Pd G3 catalyst system, wherein the sterically demanding XPhos ligand facilitated oxidative addition of the aryl halide to Pd(0). This protocol furnished boronic ester (**238**) in high yield. Subsequent Suzuki-Miyaura coupling of (**238**) with a complementary aryl fragment, catalysed by Pd_2_(dba)_3_, led to C-C bond formation, thereby yielding compound (**236**) in 73% yield. Further, oral administration of (**236**) (18.8 mg/kg QD) in an SKM-1 AML xenograft model induced 83% TGI with minimal toxicity (2% body weight loss), contrasting with the dual inhibitor ABBV-075 (**235**), which showed comparable efficacy but marked toxicity (7% weight loss, 1 mortality). Pharmacokinetic studies across species revealed favourable bioavailability (58% in mice and 77% in dogs), moderate plasma clearance, and acceptable elimination half-life (*t*_1/2_), supporting its translational potential. Compound (**236**) represents a promising candidate for further preclinical development in BET-driven malignancies (Figure 41)^148^.

**Figure 42.**
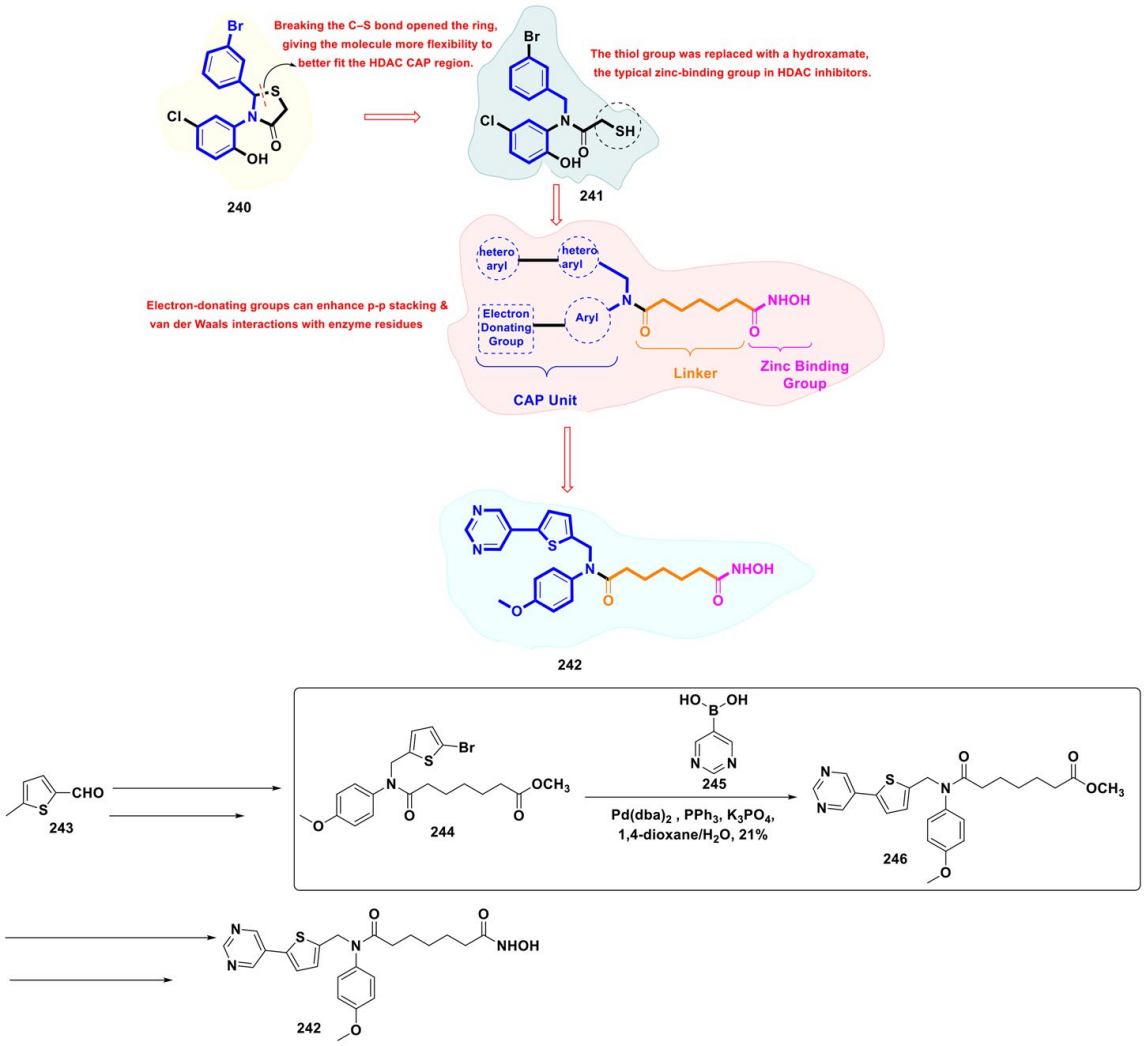
Design Strategy & Synthetic Route of Bis-Substituted Histone Deacetylase Inhibitors (the figure was drawn by the authors using chemdraw software).

Current HDAC inhibitors demonstrate clinical utility in haematologic malignancies but exhibit limited efficacy against solid tumours and inadequate suppression of metastatic progression. To address this issue, Chen *et al.* engineered a novel class of hydroxamate-based HDAC inhibitors through strategic structural modifications of the investigational ATR inhibitor CK-548 (**240**). The design involved cleavage of CK-548 (**240**) thiazolidinone ring at the C–S bond, replacing the thiol moiety with a hydroxamate ZBG linked via a bis-substituted aromatic amide scaffold. This optimisation aimed to enhance pan-HDAC inhibition while conferring antimetastatic properties. From all the synthesised scaffolds, compound (**242**) exhibited potent anticancer activity. The target hydroxamates were synthesised from bromo-thiophene aldehyde through reductive amination, acylation, and Fischer esterification steps to afford intermediate (**244**). A palladium-catalysed Suzuki–Miyaura cross-coupling was then carried out on the bromo-substituted intermediate (**244**) with arylboronic acid **245**, employing Pd_2_(dba)_3_ as the catalyst, PPh_3_ as the ligand, and K_3_PO_4_ as the base in aqueous dioxane under reflux, delivering the key biaryl scaffold (**246**) in 21% yield. Finally, the methyl ester (**246**) was transformed into the corresponding hydroxamic acid via reaction with hydroxylamine, affording hydroxamic acid **242**. Out of all synthesised hydroxamic acids, compound (**242**) exhibited selective and potent HDAC inhibition (HDAC1: 1.8 nM; HDAC2: 2.8 nM; HDAC3: 2.2 nM; HDAC6: 8.2 nM). Compound (**242**) induced histone H3/H4 hyperacetylation, suppressed migration/invasion through paxillin and MMP2/9 downregulation. Compound (**242**) demonstrated favourable PK, including broad tissue distribution and a 128-min plasma half-life (Figure 42)^149^.

**Figure 43.**
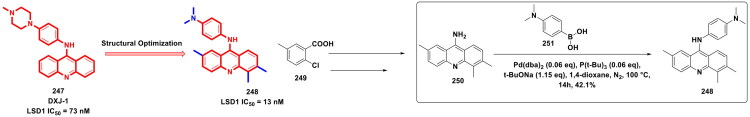
Design Strategy & Synthetic Route of Novel acridine-based LSD1 inhibitors (the figure was drawn by the authors using chemdraw software).

Dai *et al*. designed and synthesised a novel class of acridine derivatives targeting LSD1 using DXJ-1 (a previously reported compound for their anti-gastric cancer profile) as a lead compound in this study (Figure 43). Compound (**248**) exhibited the best anti-LSD1 activity (IC_50_ = 13 nM) among all these compounds. Synthetic route of compound (**248**) began with a Cu-mediated Ullmann N-arylation between the ortho-chloro benzoic acid (**249**), followed by cyclodehydrative activation, and later obtained an amine intermediate (**250**). The amine intermediate was further subjected to Buchwald–Hartwig amination to form a new C-N bond by using Pd(dba)_2_ as the palladium catalyst and P(t-Bu)_3_ as the ligand. Pd(dba)_2_ (dibenzylideneacetone palladium(0)) functions as the palladium precursor, while P(t-Bu)_3_ serves as a bulky phosphine ligand. Owing to its strong electronic donation and steric hindrance, P(t-Bu)_3_ promotes the generation of low-coordinate palladium complexes, which in turn enhance the activation of otherwise unreactive substrates such as aryl chlorides, thereby enabling efficient C–N, C–C, and C–O bond formation. Notably, t-BuONa was used as a sterically hindered inorganic base. Noteworthy to mention that compound (**248**) emerged as a potent acridine-based LSD1 inhibitor (IC_50_ = 13 nM) that suppresses gastric cancer cell stemness and migration, reduced PD-L1 expression, and enhances T cell-mediated immune responses. *In vivo,* (**248**) effectively inhibits tumour growth and boosts T cell cytokine production without significant toxicity, making it a promising candidate for gastric cancer immunotherapy^150^.

**Figure 44.**
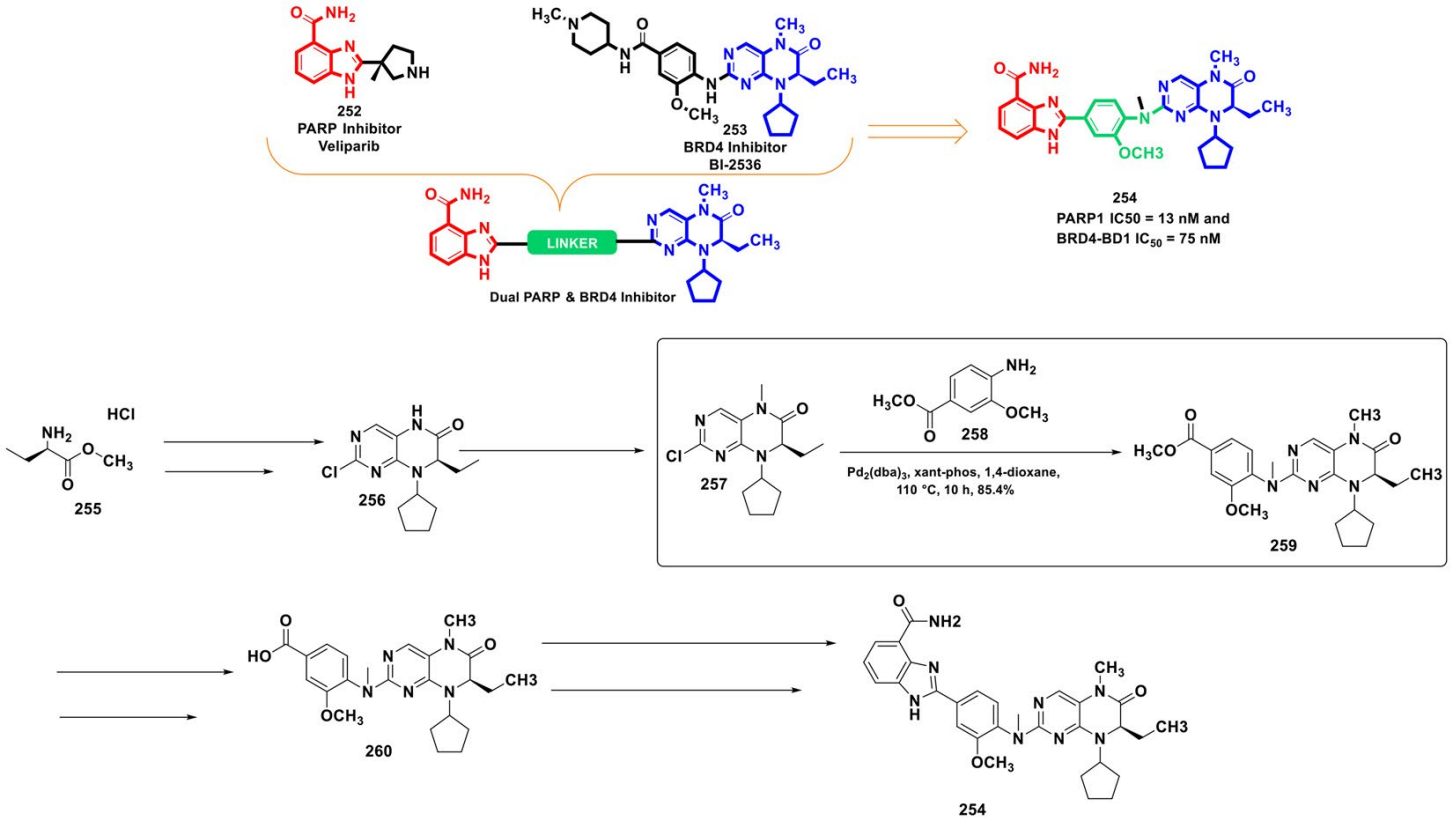
Design Strategy & Synthetic Route of Novel Dual PARP/BRD4 Inhibitor (the figure was drawn by the authors using chemdraw software).

Wang and team rationally designed and synthesised dual PARP/BRD4 inhibitors by the fusion of two pharmacophores, and utilised organopalladium catalysts, for example, Tris(dibenzylideneacetone)dipalladium(0) or [Pd_2_(dba)_3_], which plays a fundamental role in the synthesis of a cocktail of two drugs Veliparib **(252)** (PARP Inhibitor) and BI-2536 (BRD4 Inhibitor) (**253**) as depicted in Figure 44. The synthesis begins with reductive amination utilising **255,** followed by nucleophilic substitution, nitro reduction, and subsequent N-methylation to yield the N-methylated intermediate (**257**), which was further subjected to the Buchwald-Hartwig reaction by using Pd_2_(dba)_3_ as the palladium source in combination with Xantphos, a robust bisphosphine ligand, in dioxane as the reaction solvent and yielded ester intermediate **259** with an excellent yield of 85.4%. Further, the ester intermediate (**259**) underwent methylation, hydrolysis, and HATU-mediated amide coupling with 2,3-diaminobenzamide, followed by acid-induced cyclisation to yield the fused heterocycle (**254**). Noteworthy to mention that compound (**254**) exhibited potent dual PARP1/2 and BRD4 inhibitor with balanced enzymatic activity (IC_50_: PARP1 = 13 nM, PARP2 = 4.7 nM, BRD4 = 101 nM). Compound (**254**) significantly suppressed the proliferation of BRCA-proficient pancreatic cancer cell lines (CFPAC-1, Capan-1, SW1990) with IC_50_ values in the low micromolar range and demonstrated superior efficacy to JQ-1, Olaparib, or their combination. Mechanistically compound (**254**) exhibited significant antitumor efficacy both *in vitro* and *in vivo*^151^.

### Pd(PPh_3_)_4- _assisted synthesis of small-molecule epigenetic inhibitors

In modern synthetic chemistry, the construction of C-C bonds often centres on tetrakis(triphenylphosphine)palladium(0) [Pd(PPh_3_)_4_], a palladium(0) complex with a tetrahedral geometry featuring four monodentate triphenylphosphine ligands. Renowned for its role in homogeneous catalysis, this complex is indispensable in cross-coupling methodologies like Suzuki, Heck, and Sonogashira reactions. This subsection underscores the utility of Pd(PPh_3_)_4_ as the palladium catalyst to furnish small molecule chemical architectures as epigenetic inhibitors.

**Figure 45.**
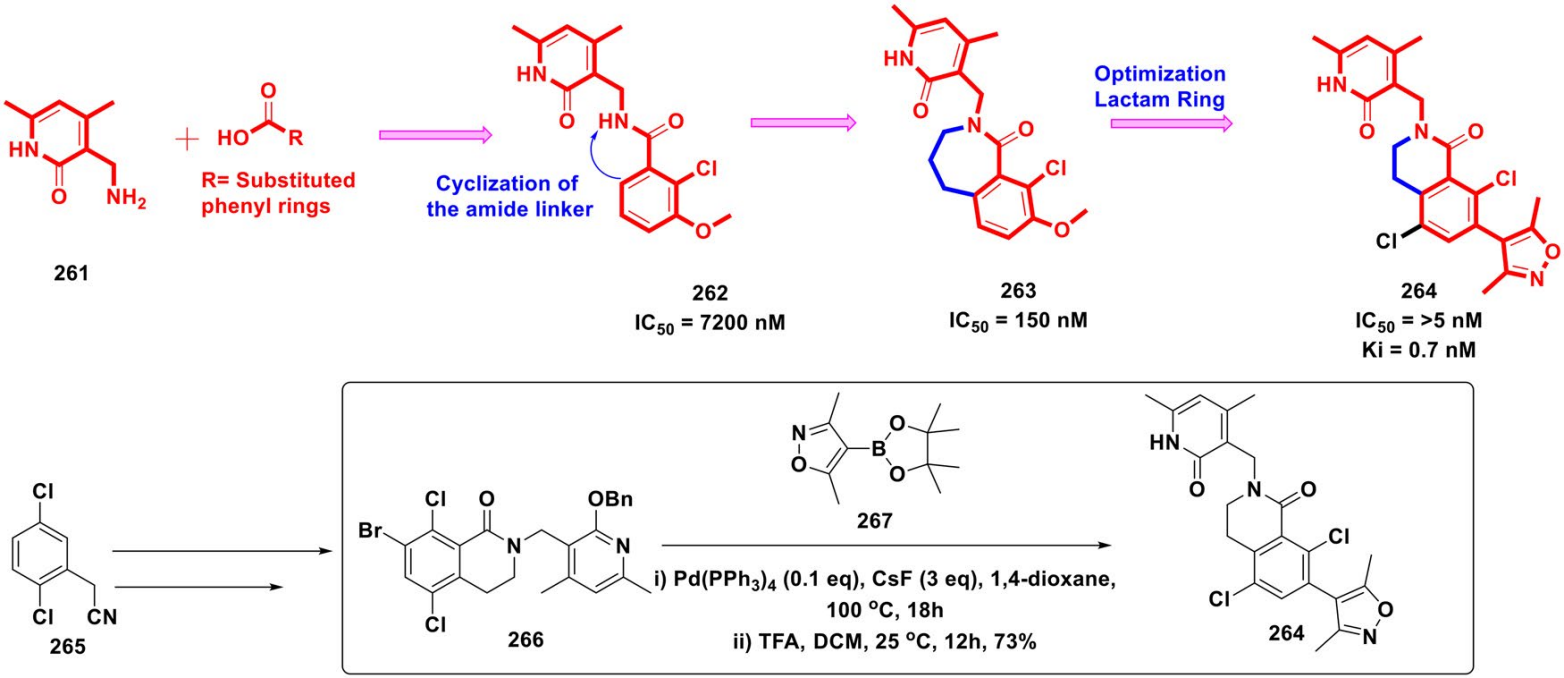
Design Strategy & Synthetic Route of Pyridone-containing 3,4-Dihydroisoquinoline-1(2H)-ones based Novel class EZH2 Inhibitor (the figure was drawn by the authors using chemdraw software).

Kung *et al.* conducted a lead modification study employing a previously existing EZH2 inhibitor as the lead to generate a series of lactam-containing EZH2 inhibitors (Figure 45). Computational torsional angle analysis and ligand cyclisation strategy were used for the design of the compounds. The study aimed to improve ligand lipophilic efficiency as well as the potency of the lead structural template. Resultantly, a magnificent EZH2 inhibitor (**264**) was identified that manifested remarkable efficacy in the diffuse large B-cell lymphoma Karpas-422 tumour model. Gratifyingly, the aim of the study was accomplished as the compound (**264**) manifested remarkable *in vivo* antitumor efficacy in a diffuse large B-cell lymphoma Karpas-422 tumour model and was endowed with improved LipE and on-target potency in both biochemical and cellular readouts. Organopalladium catalysis proved to be instrumental in the synthesis of the compound (**264**) as it catalysed the penultimate step of the multistep synthetic route. The route began with nickel chloride-mediated nitrile reduction of 2-(2,5-dichlorophenyl)-acetonitrile (**265**), followed by a reaction sequence comprising steps *viz.*, concomitant methyl carbamate protection, intramolecular Friedel − Crafts acylation, NBS-mediated bromination, and lactam nitrogen alkylation. The aforementioned reaction sequence culminated in the identification of an adduct (**266**) that was leveraged for Suzuki cross-coupling reactions with 3,5-dimethyl-4–(4,4,5,5 tetramethyl-1,3,2-dioxaborolan-2-yl) isoxazole (**267**). For this reaction, Pd (PPh_3_)_4_ was used as the organopalladium catalyst, and caesium fluoride was used to activate the boron reagent via formation of boronated anions that are more reactive in transmetallation. Notably, transmetallation in the Suzuki cross-coupling reaction is required for the transfer of the organic group to palladium. Anhydrous 1,4-dioxane was used as the solvent for the reaction, and the reaction was performed in a sealed tube at 100 °C for 18h to afford the formation of the adduct. Gratifyingly, the adduct was generated in good yield (73%) that allowed the research group to proceed towards the final step of the synthetic pathway to afford benzyl ether deprotection via TFA^152^.

**Figure 46.**
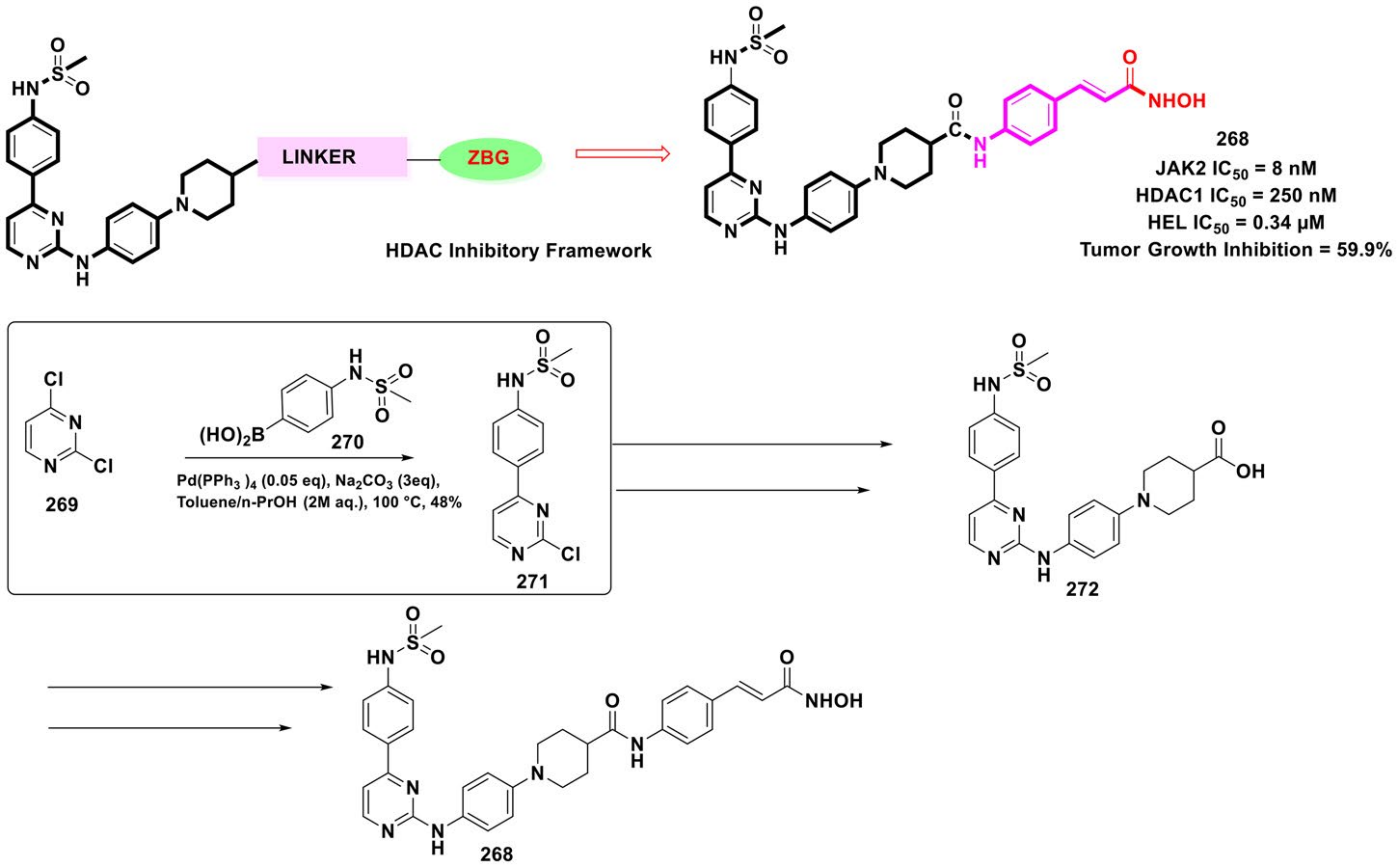
Design Strategy & Synthetic Route of Dual JAK 2 & HDAC Inhibitor (the figure was drawn by the authors using chemdraw software).

Sheng *et al.* designed dual JAK2 and HDAC inhibitors by the HDAC inhibitory structural architecture, in which ruxolitinib was used as a cap unit, different linkers were used to optimise the dual potency of compounds, and hydroxamic acid was used as a ZBD (Figure 46). Compound (**268**), incorporating an N-phenylmethanesulfonamide group at the terminal position of the Cap unit and an acrylamide linker, demonstrated strong dual inhibition of JAK2 and HDAC. Synthesis of compound **268** initiated with a multi-step route that relied on organopalladium-catalysed cross-coupling to assemble the core framework. The sequence began with a Suzuki–Miyaura coupling between 2,4-dichloropyrimidine (**269**), a pyrimidine derivative, and aryl boronic acid (**270**), using tetrakis(triphenylphosphine)palladium(0) [Pd(PPh_3_)_4_] as the catalyst in a toluene/n-PrOH mixture with aqueous Na_2_CO_3_ at 100 °C. The resulting biaryl intermediate (**271**) then underwent nucleophilic substitution to produce an ester intermediate, which was hydrolysed with LiOH to yield the corresponding carboxylic acid (**272**). This acid was condensed with an ester moiety to generate another ester intermediate, which was finally treated with a methanolic hydroxylamine solution at room temperature to afford the target compound (**268**). Compound (**268**) showed potent activity against both cancer and invasive fungal infection and selectively inhibited JAK2 (IC_50_ = 8 nM) and HDAC1 (IC_50_ = 250 nM), outperforming single-target inhibitors in HEL cells (IC_50_ = 0.34 μM) by inducing apoptosis, G_2_/M-phase arrest, histone H3/H4 acetylation, and STAT5 dephosphorylation. During *in vivo* assays, compound (**268**) suppressed HEL xenograft growth by 59.9%, extended AML mouse survival, and spared normal tissues. Compound (**268**) also synergized with fluconazole against drug-resistant *Candida albicans* (FIC ≤ 0.07), enhancing antifungal activity and reducing biofilm formation, making compound (**268**) a promising dual-action agent for leukaemia–IFI therapy^153^.

**Figure 47.**
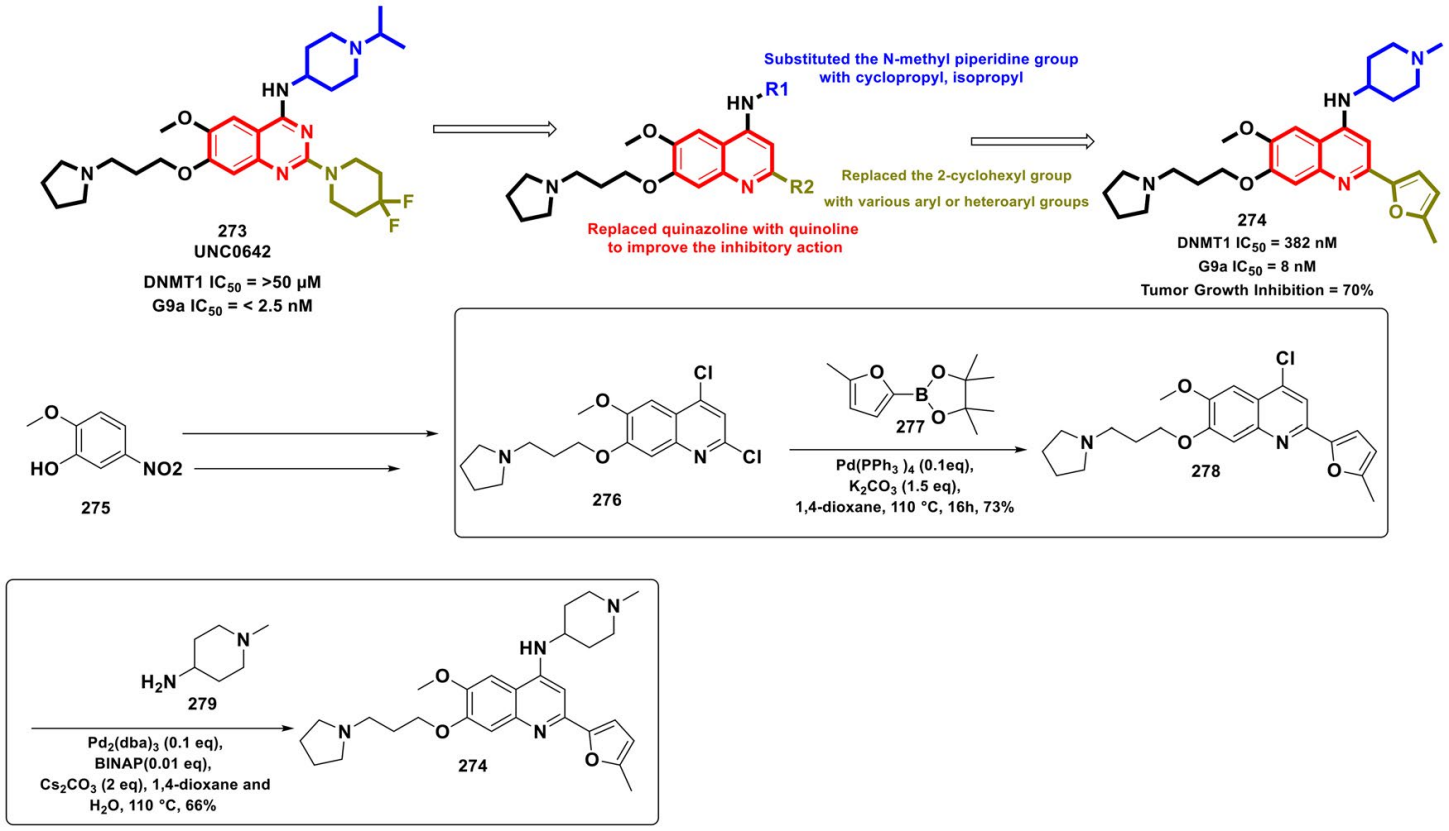
Design Strategy & Synthetic Route of Reversible DNA Methyltransferase and Lysine Methyltransferase G9a Inhibitors (the figure was drawn by the authors using chemdraw software).

Recent scientific reports suggest that DNMTs catalyse DNA methylation at CpG sites, silencing tumour suppressor genes by blocking transcriptional machinery, while G9a methylates histone H3 at lysine 9 (H3K9me2), promoting heterochromatin formation and reinforcing gene repression. These mechanisms act synergistically to induce tumorigenesis. On this factual basis, Oyarzabal and his team developed quinazoline-based chemical adducts designed to concurrently inhibit histone 3 lysine 9 methyltransferase (G9a) and DNMTs at nanomolar potencies, leveraging a synthetic strategy anchored in organopalladium catalysis (Figure 47). Among these, compound (**274**) emerged as a dual inhibitor with striking *in vitro* activity (IC_50_ = 382 nM for DNMT1; IC_50_ = 8 nM for G9a) and demonstrated superior *in vivo* efficacy in a human AML xenograft model, achieving 70% TGI (*p* < 0.05) at a low intravenous dose (2.5 mg/kg daily for 21 days), reducing tumour volumes to 940 ± 435 mm³ compared to 3096 ± 1399 mm³ in controls. Compound (**273**) showed only 45% inhibition despite a higher dose (30 mg/kg/day) and prolonged administration. The synthesis of compound (**274**) hinged on two pivotal palladium-catalysed steps as depicted in Figure 47: a Suzuki coupling (Pd(PPh_3_)_4_/K_2_CO_3_) to generate intermediate 73% yield (**278**) from precursor (**276**), followed by a Buchwald-Hartwig amination (Pd_2_(dba)_3_/BINAP/Cs_2_CO_3_) to furnish intermediate (**274**) in 66% yield. While BINAP, typically employed for asymmetric amination, was utilised here to stabilise the palladium complex and ensure efficient coupling, and its role underscored the precision required to access structurally intricate intermediates^154^.

**Figure 48.**
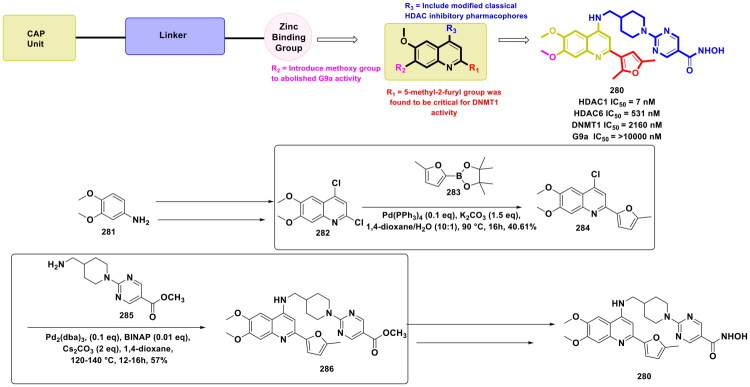
Design Strategy & Synthetic Route of Histone Deacetylases, DNA Methyltransferase 1, and Lysine Methyltransferase G9a Inhibitor (the figure was drawn by the authors using chemdraw software).

In another study, Oyarzabal *et al.* rationally designed a novel class of quinoline-derived compounds with polypharmacological activity against concurrently HDACs, DNMT1, and lysine methyltransferase G9 through strategic structural modifications (Figure 48). In this study, the quinoline core was selected as a privileged scaffold due to its demonstrated compatibility with DNMT1 and G9 binding, as observed in predecessors like compound CM-272 and CM-579, while its rigidity allowed precise substitutions at different positions without compromising target engagement. These polypharmacological compounds were synthesised by organopalladium catalysis. Two different organopalladium catalysts were used in two different steps. Notably, Pd(PPh_3_)_4_ was utilised for the formation of a C-C bond by the Suzuki coupling reaction to generate intermediate (**284**). The reaction was performed at reflux by using dioxane and water as solvents, and Pd_2_(dba)_3_ was used with BINAP to form a C-N bond through Buchwald − Hartwig amination reaction. The reaction was performed at 120 °C-140 °C, and caesium carbonate was used as a base in the reaction mixture to generate ester intermediate (**286**), which further transformed into final hydroxamic compounds with good yield. Among the synthesised compounds, compound (**280**) emerged as a standout dual DNMT1/HDAC inhibitor, demonstrating striking antiproliferative potency, surpassing single-target inhibitors and validating the synergistic therapeutic impact of concurrent epigenetic pathway disruption. During *in vivo* studies, (**280**) exhibited significant antitumor efficacy in a MM xenograft model, and it was supported by a tailored formulation vehicle which enables favourable pharmacokinetics and a > 1 log unit therapeutic window, indicative of selective on-target activity^155^.

**Figure 49.**
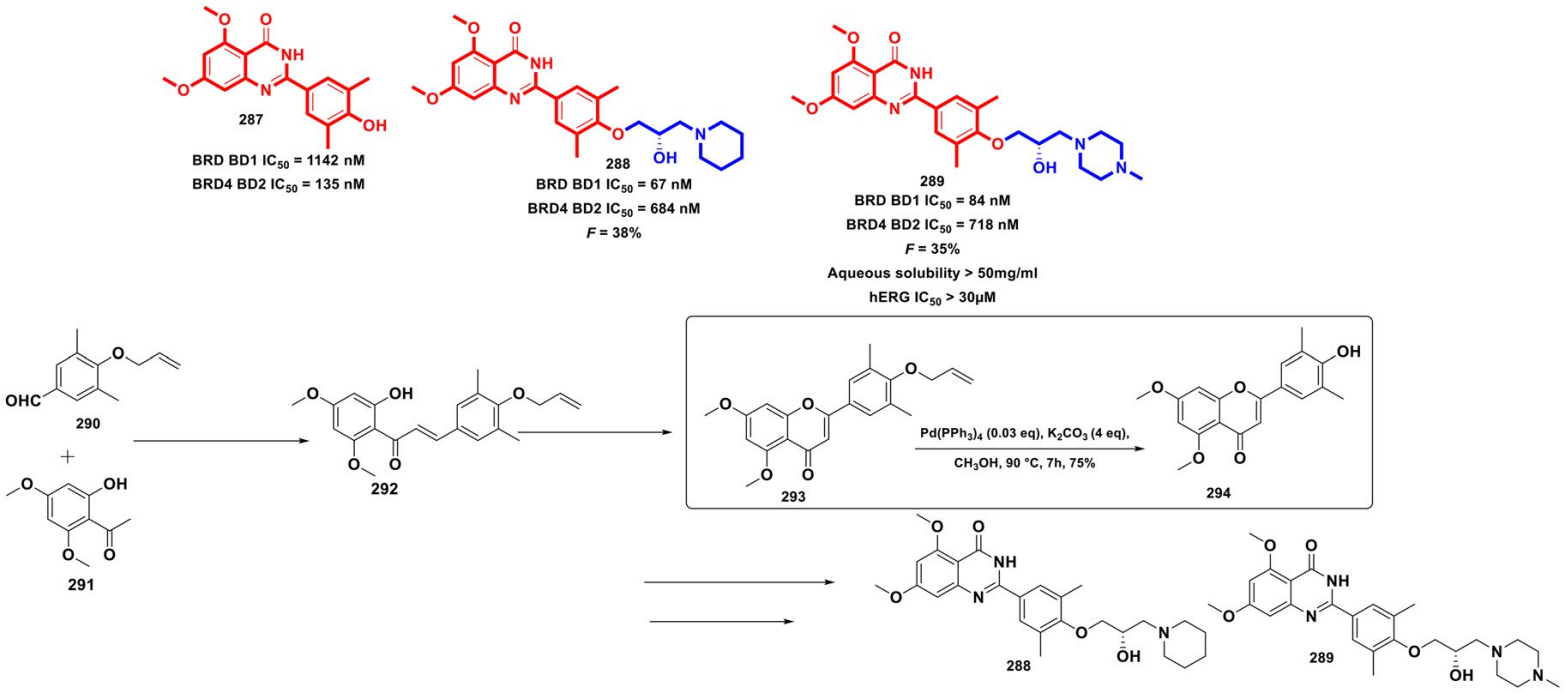
Design Strategy & Synthetic Route of Potent and Selective BRD4 Inhibitors (the figure was drawn by the authors using chemdraw software).

Zhou and team rationally design and develop a novel class of BRD4 inhibitors through scaffold hopping. To develop novel BRD4 inhibitors, a quinazolin-4-one core with a chromone scaffold was utilised to improve BRD4 selectivity, elevate inhibitory potency, and improve PK properties, as depicted in Figure 49. The synthetic route commenced with the formation of the chalcone linkage between the aldehyde (**290**) and acetophenone (**291**) through a Claisen-Schmidt condensation, and intramolecular Michael cyclisation to yield intermediate (**293**). Further deprotection of the allyl aryl ether group of the flavone intermediate was done by using Palladium-Tetrakis(triphenylphosphine), as palladium-catalysed deallylation to generate the phenol intermediate (**294**). Noteworthy to mention that the organopalladium catalyst was used as an effective scavenger in the deprotection of allyl-protected functional groups. Further, intermediate (**294**) was subsequently alkylated at the phenolic hydroxyl group via a Williamson ether synthesis and producing final compounds (**288**) and (**289**). Noteworthy to mention that compounds (**288**) and (**289**), displaying IC_50_ values of 67–84 nM, have strong selectivity over other BET and non-BET proteins. In cellular assays, both compounds suppressed TLR3-induced proinflammatory gene expression, with compound (**289**) showing superior potency (IC_50_ = 0.28 μM for CIG5; 0.31 μM for IL-6). Both compounds also exhibited favourable pharmacokinetics, including 35–38% oral bioavailability, >50 mg/mL ueous solubility, low microsomal clearance, and high metabolic stability^156^.

**Figure 50.**
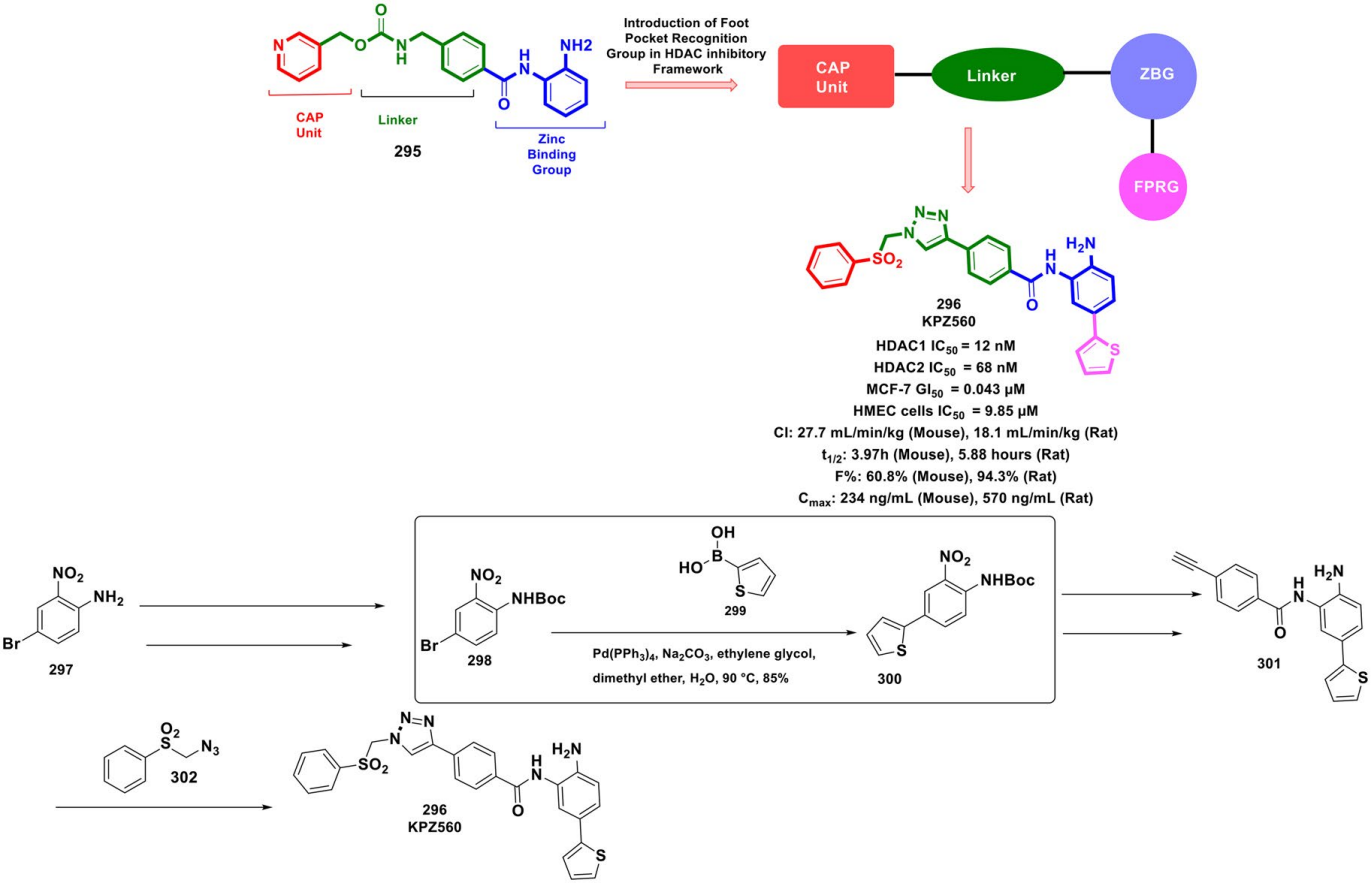
Design Strategy & Synthetic Route of Selective HDAC 1 and 2 Inhibitors (the figure was drawn by the authors using chemdraw software).

Suzuki and colleagues adopted a click chemistry-based approach to rationally design & develop selective HDAC1/2 inhibitors (Figure 50). Faqor this medicinal campaign, a classical HDAC inhibitor framework was used, which consisted of cap group, a linker, and a ZBG, but also introduced a unique structural feature, the Foot Pocket Recognition Group, to improve selectivity and binding strength. From the synthesised compound library, compound KPZ560 (**296**) emerged as the most potent HDAC inhibitor in biological evaluations. The synthesis of KPZ560 (**296**) involved a multistep strategy that began with the protection of the amino group of 4-bromo-2-nitroaniline (**297**) afforded an amine-protected intermediate (**298**), which was further subjected to palladium-catalysed Suzuki Miyaura cross-coupling reactions with 2-thiopheneboronic acid in the presence of Pd(PPh_3_)_4_ as organopalladium catalyst, sodium carbonate as base and ethylene glycol dimethyl ether as solvent at reflux to generate corresponding aryl substituted derivatives (**300**) with 85% yield. Further, subsequent nitro reduction, acid-amine coupling and copper-catalysed click reactions yielded the final compound KPZ560 (**296**). Noteworthy to mention that compound KPZ560 (**296**) exhibited significant biological effects through its unique two-step slow binding inhibition mechanism of HDAC1/2, inducing dose- and time-dependent histone acetylation at H3K9 and H3K18 and selectively acetylating p53 without affecting NF-κB or α-tubulin. KPZ560 (**296**) exhibited strong anti-proliferative activity in MCF-7 breast cancer cells while sparing normal HMECs, which highlights its therapeutic potential^157^.

**Figure 51.**
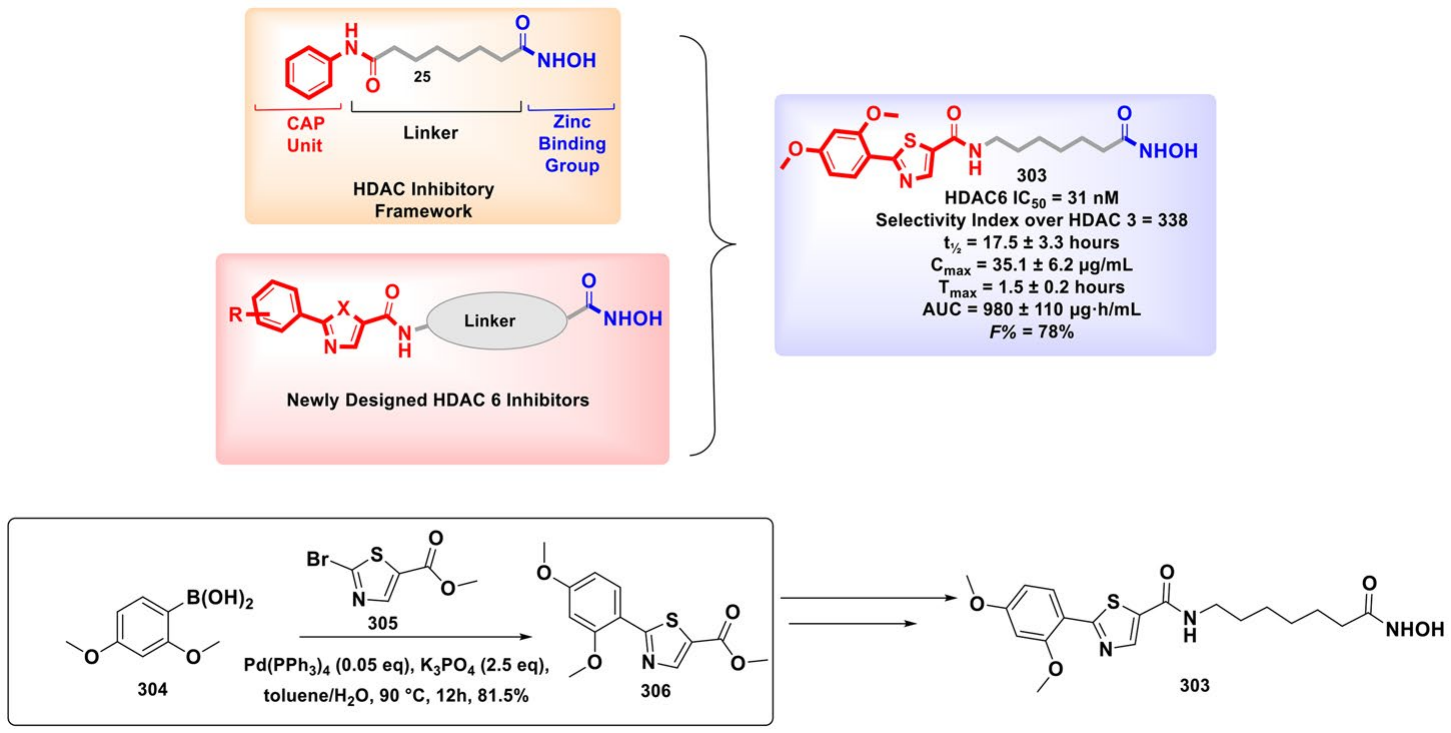
Design Strategy & Synthetic Route of Novel HDAC6 Inhibitor (the figure was drawn by the authors using chemdraw software).

Jianjun *et al*. pioneered the rational design of isoform-selective HDAC6 inhibitors to address different shortcomings linked with non-selective epigenetic inhibitors (Figure 51). In this study, a series of 2-phenylthiazole analogues were synthesised; out of this series, only compound (**303**) exhibited the most potent activities against HDAC6. The synthesis of compound (**303**) employed a palladium-catalysed Suzuki–Miyaura coupling reaction, where 2,4-dimethoxyphenylboronic acid (**304**) and 2-bromothiazole-5-carboxylate (**305**) undergo regioselective C–C bond formation mediated by Pd(PPh_3_)_4_ in a biphasic toluene/water system with K_3_PO_4_ at 90 °C, furnishing the aryl-thiazole core (**306**). Further, ester saponification, amide coupling with methyl 7-aminoheptanoate, hydroxamic acid formation using NH_2_OTHP, and final THP deprotection with HCl afforded the target hydroxamic acid compound (**303**). Compound (**303**) is a next-generation selective HDAC6 inhibitor (IC_50_ = 31 nM, 338-fold selectivity over HDAC3) showing potent activity against HDAC inhibitor–resistant gastric cancer and B16-F10 melanoma cells and inducing apoptosis by causing G_2_/M arrest. In animal studies, it inhibited 63% of melanoma tumour growth without toxicity and showed synergy with PD-L1 inhibitors. Mechanistically, it enhanced α-tubulin and Ku70 acetylation, and despite low oral bioavailability, its good solubility and moderate half-life make it a promising oncologic candidate^158^.

**Figure 52.**
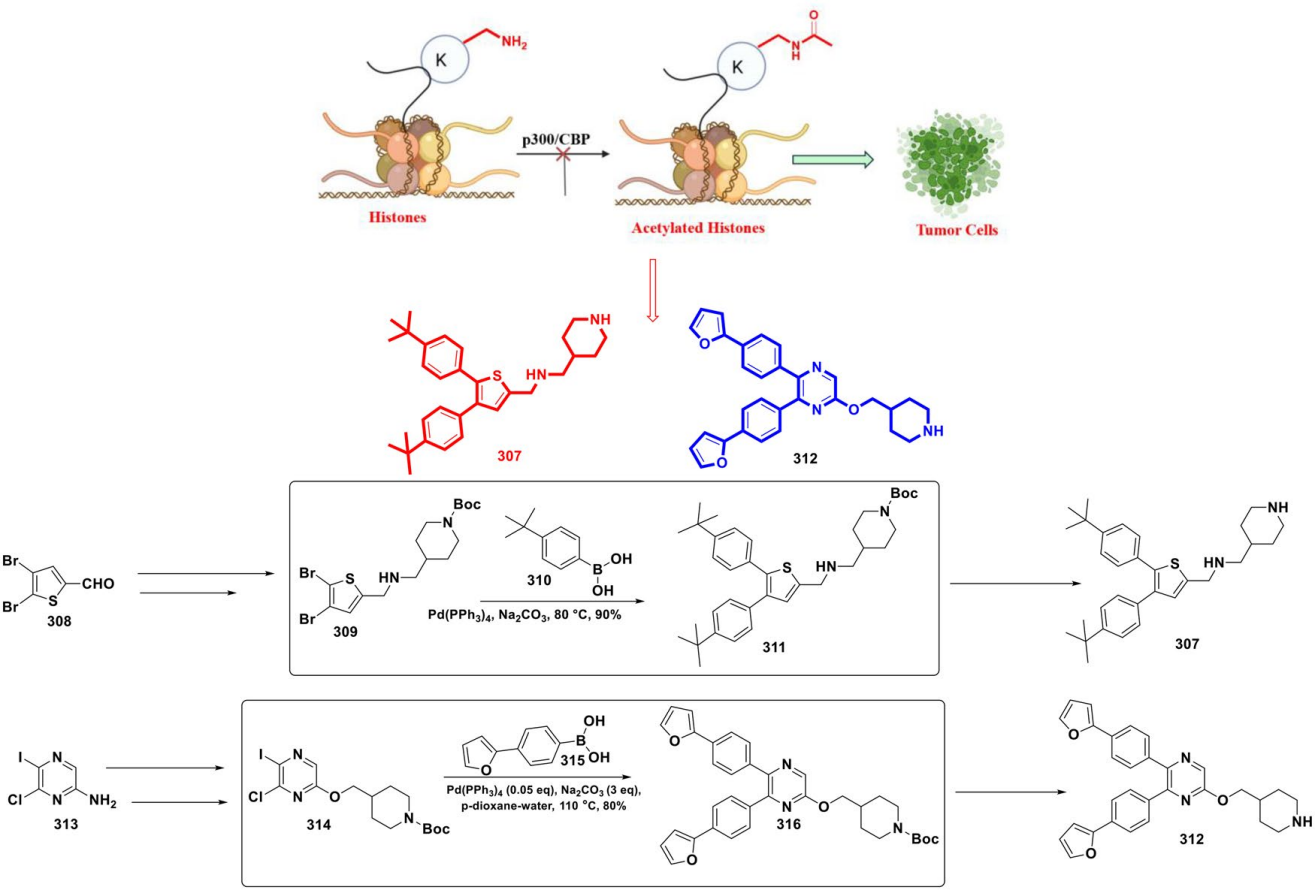
Design Strategy & Synthetic Route of Novel Histone Acetyltransferases p300/CB (the figure was drawn by the authors using chemdraw software).

Song *et al.* discovered a novel series of inhibitors of p300/CBP HAT that are competitive against the histone substrate. Previously reported potent and selective p300/CBP HAT inhibitor exhibited limitations, including poor cell permeability and structural liabilities. A series of thiophene-2-carbamide derivatives was synthesised and subjected to a comprehensive SAR investigation (Figure 52), with compound (**307**) emerging as the most pharmacologically potent compound. The synthesis of compound (**307**) entailed a strategically composed sequence of chemical transformations. The synthetic route commenced with 4,5-dibromothiophene-2-carbaldehyde (**308**), which is reduced to a benzylic alcohol, then chlorinated, and further coupled with a Boc-protected amine to yield the benzylated amine (**309**). Next, an organopalladium-mediated Suzuki cross-coupling reaction was performed employing tetrakis(triphenylphosphine)palladium(0) [Pd(PPh_3_)_4_] as the catalyst, dioxane: water as solvent system at reflux to generate intermediate (**311**) with 90% yield. Finally, the Boc deprotection under acidic conditions through TFA to deliver the target compound (**307**). Notably, compound (**307**) is a potent p300-HAT inhibitor (IC_50_ = 620 nM) that selectively blocks H3K9, H3K18, and H3K27 acetylation, disrupts histone substrate binding, and suppresses the ER-driven oncogenic transcription. Functionally, compound (**307**) effectively inhibited proliferation of ER^+^ breast cancer, tamoxifen-resistant variants, pancreatic cancer, and RUNX1-ETO leukaemia with EC_50_ values of 1.0–3.4 μM, making it a therapeutic lead for p300/CBP-dependent cancers^159^.

Yongcheng Song and colleagues discovered that 1,4-pyrazine/pyridine derivatives (Figure 52), particularly compound (**312**), function as highly selective p300-HAT inhibitors during library screening, with para-bromine modifications on the pyrazine scaffold proving essential for enhanced potency (IC_50_ = 1.4 µM). Notably, compound (**312**) was synthesised from 6-chloro-5-iodopyrazin-2-amine via intermediate transformations, followed by a key Pd(PPh_3_)_4_-catalysed Suzuki–Miyaura coupling with (4-(furan-2-yl)phenyl)boronic acid in a dioxane–H_2_O system at 110 °C to yield the aryl-substituted intermediate, which, after Boc deprotection, furnished the final amine derivative **312**. Gratifyingly, compound (**312**) showed high specificity, with minimal activity against other HAT isoforms and LSD1 (IC_50_ > 50 µM), while selectively inhibiting H3 acetylation, particularly H3K27. The compound (**312)** ability to disrupt epigenetic signalling via p300/CBP inhibition, coupled with its selective cytotoxicity, makes it a mechanistic probe for studying acetylation-dependent pathways and a potential therapeutic candidate for diverse cancers^160^.

**Figure 53.**
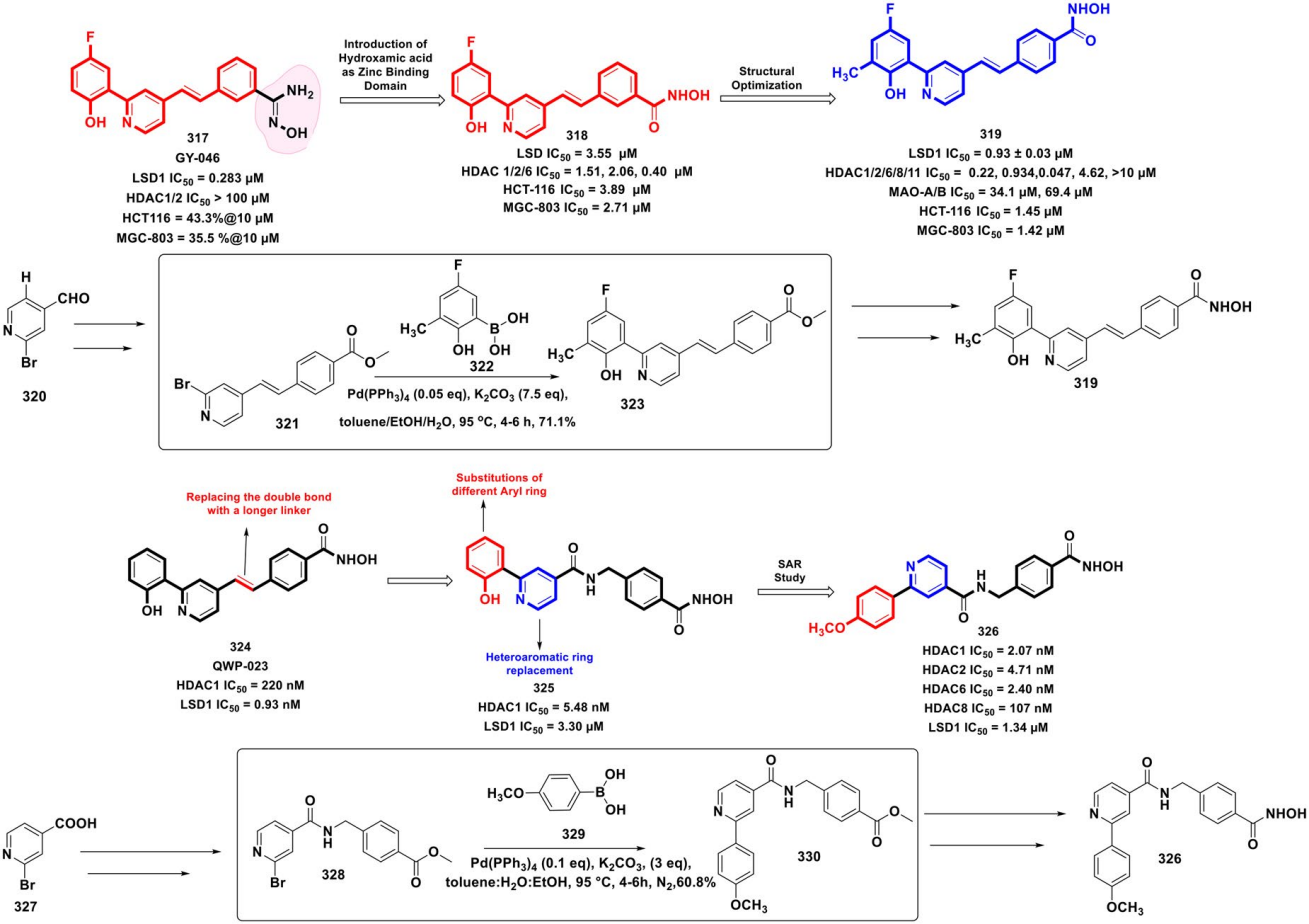
Design Strategy & Synthetic Route of Dual LSD1 & HDAC Inhibitor (the figure was drawn by the authors using chemdraw software).

Recent studies reveal that HDACs, particularly HDAC5, stabilise LSD1 activity, nurturing oncogenic pathways; simultaneous inhibition disrupts this synergy, overcoming limitations of earlier dual inhibitors that suffered from poor HDAC potency and metabolic instability. To address this, Guan *et al.* designed orally bioavailable inhibitors inspired by the LSD1 inhibitor GY-046, which exhibit nanomolar-range activity against both HDAC and LSD1, high selectivity over MAO-A/B, and potent antiproliferative effects in MGC-803 and HCT-116 cells (Figure 53). Compound (**319**) modulates apoptosis-related proteins (Bcl-2, Bax) and epithelial-mesenchymal transition markers (Vimentin, ZO-1, E-cadherin), suppressing migration and colony formation in gastric cancer models. The synthesis of compound (**319**) initiated with the condensation of 4-bromo-2-formylpyridine (**320**) and after various intermediate transformations, it followed a key Pd(PPh_3_)_4_-catalysed Suzuki–Miyaura cross-coupling of vinylpyridine intermediate (**321**) with (5-fluoro-2-hydroxy-3-methylphenyl)boronic acid in a toluene–ethanol–water system at 95 °C, affording ester intermediate (**323**), which was subsequently converted to the final hydroxamic acid derivative^161^.

Building on prior work, Guan *et al.* refined the structural framework of compound (**324**) to enhance dual-targeting efficacy against LSD1 and HDAC, culminating in the discovery of compound (**326**), a markedly superior inhibitor. Notably, compound (**326**) displayed potent HDAC inhibition (HDAC1 IC_50_ = 2.07 nM; HDAC6 IC_50_ = 2.40 nM) and LSD1 suppression (IC_50_ = 1.34 μM) and exhibited strong antiproliferative effects in haematologic and solid tumour cells (IC_50_ = 0.14–0.57 μM). It also induced mitochondrial apoptosis, G2/M arrest, and significant tumour growth inhibition in xenograft models (71.5% in MGC-803; 57.6% in HCT-116), outperforming SAHA during *in vivo* studies. The synthesis of compound (**326**) heavily relied on organopalladium catalysis. The synthetic route commenced with acid amine coupling to yield ester intermediate, which was further subjected to Pd(PPh_3_)_4_-mediated Suzuki-Miyaura coupling strategy employed for synthesis of intermediate (**330**) by using potassium carbonate as base, toluene, water, and ethanol as solvents, at 95 °C under a nitrogen environment. Intermediate (**330**) further converted into the final desirable compound through hydroxamate formation with NH_2_OK, KOH, anhydrous CH_3_OH–CH_2_Cl_2_, 0 °C to rt, for 1 h (**326**) in 53.3% yield. These two studies highlight the important role of palladium-mediated C–C bond formation in constructing multifunctional epigenetic modulators, underscoring its utility in precision drug design for oncology (Figure 53)^162^.

**Figure 54.**
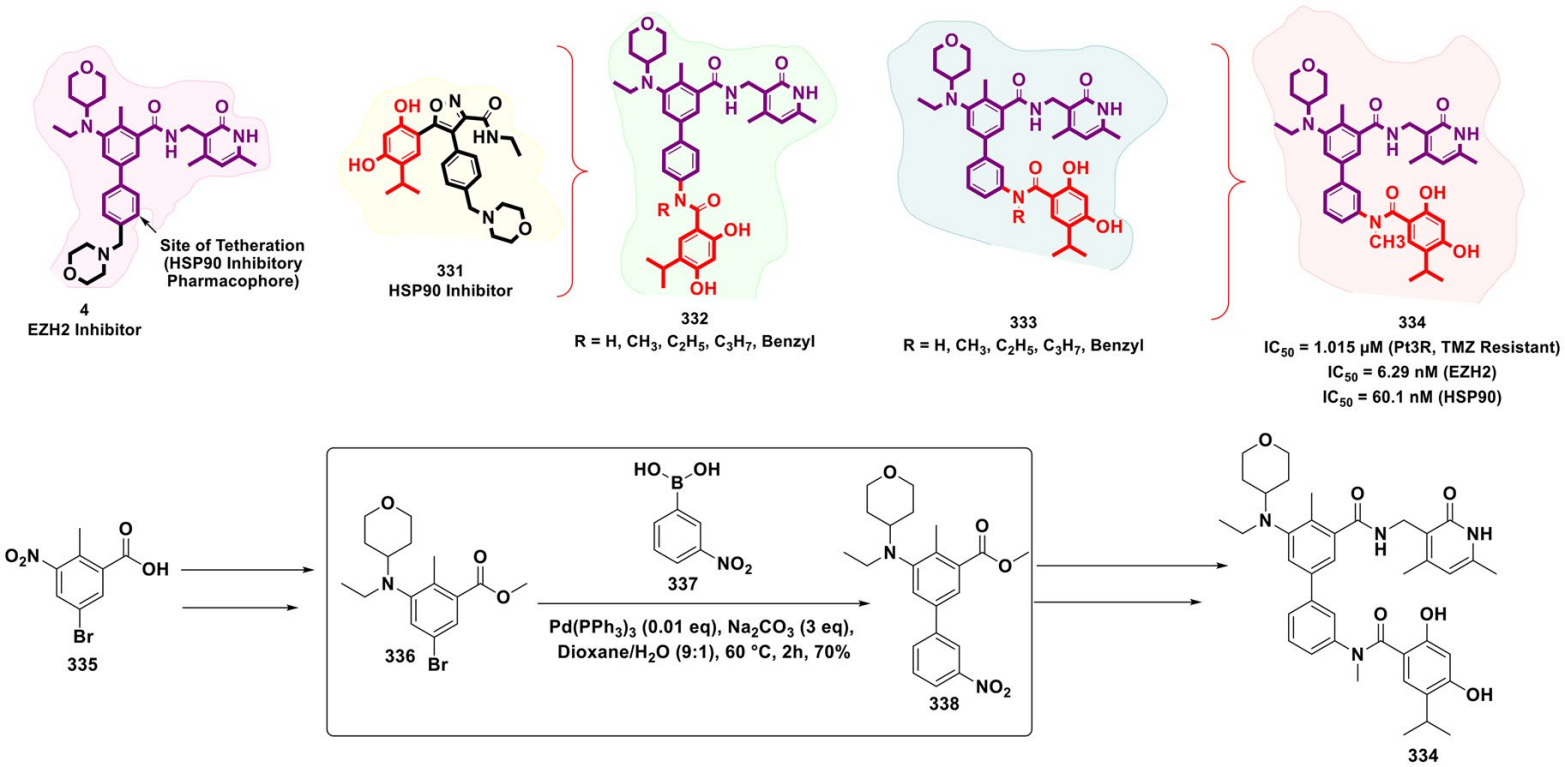
Design Strategy & Synthetic Route of First-in-Class Dual EZH2-HSP90 Inhibitor (the figure was drawn by the authors using chemdraw software).

Despite the therapeutic promise of dual-targeting strategies in GBM, clinical translation has been hindered by suboptimal potency, inadequate BBB penetration, and acquired resistance to existing therapies. Addressing these limitations, Sharma *et al*. developed compound (**334**), a first-in-class dual EZH2-HSP90 inhibitor engineered through rational hybridisation of Tazemetostat (**4**) structural framework with resorcinol-derived pharmacophores (Figure 54). Systematic evaluation of synthesised analogs revealed compound (**334**) as a potent molecule, exhibiting nanomolar dual enzymatic inhibition (EZH2 IC_50_ = 6.29 nM; HSP90 IC_50_ = 60.1 nM) and potent antiproliferative activity against temozolomide-resistant Pt3R GBM cells (IC_50_ = 1.015 μM), surpassing Tazemetostat (**4**) efficacy. Mechanistically, compound (**334**) induced M-phase arrest by downregulating CDK1/cyclin B1, disrupting kinetochore assembly, impairing DNA repair, and elevating mitochondrial ROS, leading to apoptosis. *In vivo*, it effectively inhibited Pt3R tumour growth, crossed the blood–brain barrier, and outperformed tazemetostat, highlighting its promise for GBM therapy. The synthesis of compound (**334**) strategically employed organopalladium catalysis for efficient scaffold assembly. After preparing intermediate (**336**) via reductive amination, a key Suzuki–Miyaura cross-coupling between (**336**) and 3-nitroboronic acid (**337**) using Pd(PPh_3_)_3_ and Na_2_CO_3_ in a dioxane/water system at 60 °C afforded the biphenyl scaffold (**338**) in 85–90% yield. Further, subsequent amidation, hydrolysis, and debenzylation furnished the final compound (**334**) in 48% overall yield, exemplifying the precision and versatility of Pd-catalysed C–C bond formation in complex molecule construction^163^.

**Figure 55.**
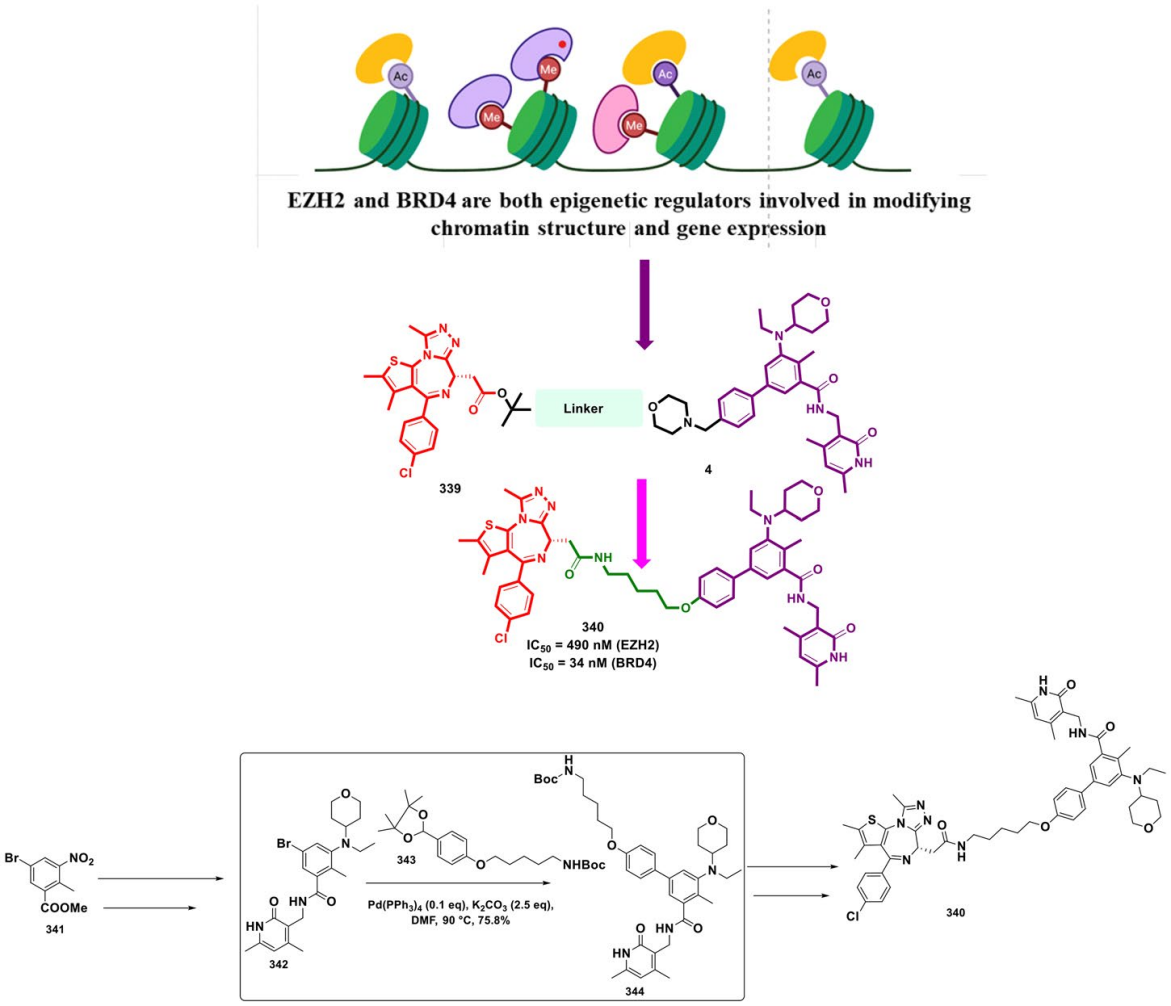
Design Strategy & Synthetic Route of Dual EZH2/BRD4 Inhibitor (the figure was drawn by the authors using chemdraw software).

Guo *et al.* commenced a medicinal chemistry campaign to develop dual EZH2/BRD4 inhibitors for solid tumours, through the cocktail of EPZ-6438 (EZH2 inhibitor) (**4**) and JQ1 (BRD4 inhibitor) (**339**) via variable linkers such as carbon or polyethylene glycol chains (Figure 55). Systematic scaffold optimisation culminated in the identification of compound (**340**), a potent dual inhibitor with IC_50_ values of 0.49 μM (EZH2) and 0.034 μM (BRD4). *In vitro*, compound (**340**) suppressed H3K27me3 (EZH2 marker) and modulated BRD4 pathways, showing stronger antiproliferative effects in pancreatic, colorectal, and lung cancer models than reference drugs either alone or combined. Notably, *in vivo*, it has achieved 63.4% TGI in A549 and AsPC-1 xenografts without toxicity. It is imperative to point out that compound (**340**) was synthesised by a multistep synthetic route that incorporates classical organic transformations such as tosylation, Suzuki-Miyaura cross-coupling, Boc-deprotection, and amide bond formation. A Suzuki-Miyaura coupling was performed between the halogenated core scaffold (**342**) and intermediate (**343**) using Pd(PPh_3_)_4_ as the catalyst and K_2_CO_3_ as the base in DMF, affording the biphenyl-linked intermediate (**344**), which was further transformed into the final compound (**340**) by amide coupling of the second intermediate with an excellent 75.8% yield. This convergent synthetic strategy efficiently integrates the pharmacophores of EPZ6438 (EZH2 inhibitor) (**4**) and JQ1 (BRD4 inhibitor) (**339**), resulting in a novel bivalent molecule with potent dual target activity^164^.

**Figure 56.**
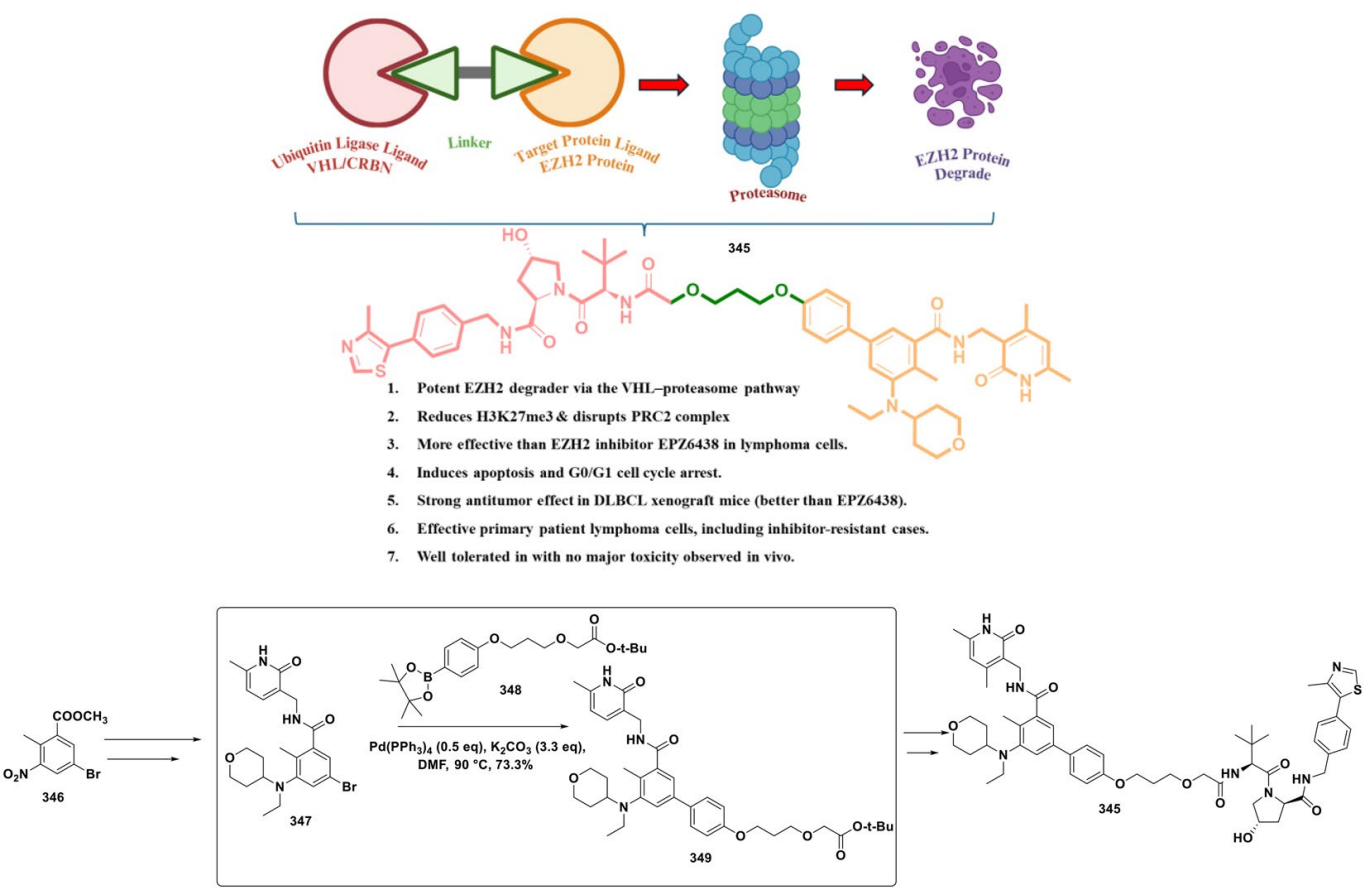
Design Strategy & Synthetic Route of VHL-Based EZH2 Degraders (the figure was drawn by the authors using chemdraw software).

Wen *et al*. reported the rational design and synthesis of PROTAC molecules for complete EZH2 protein degradation in lymphoma as shown in Figure 56. Among them, a VHL-based degrader (**345**) showed potent, dose-dependent EZH2 degradation, strong antiproliferative and apoptotic effects, and superior tumour suppression to EPZ6438 (**4**) without toxicity in lymphoma xenograft models. It is important to highlight that the synthesis of compound (**345**) involves a multistep process that strategically incorporates organopalladium chemistry, particularly through Suzuki coupling reactions, which are essential in constructing biaryl intermediates. The synthesis of compound (**345**) began with nitro reduction, followed by reductive amination, and ester hydrolysis–coupling steps to yield intermediate (**347**). The key transformation involved a Pd(PPh_3_)_4_-catalysed Suzuki–Miyaura cross-coupling between aryl halide (**347**) and boronic acid (**348**), enabling efficient construction of the biaryl scaffold essential for linker assembly (**349**) and later final PROTAC formation by further modifications. This step was crucial for assembling the biaryl core structure that serves as a foundational scaffold for linker attachment and subsequent conjugation with the EZH2 and VHL-binding motifs, ultimately affording compound (**345**) in a yield of 64.1%^165^.

**Figure 57.**
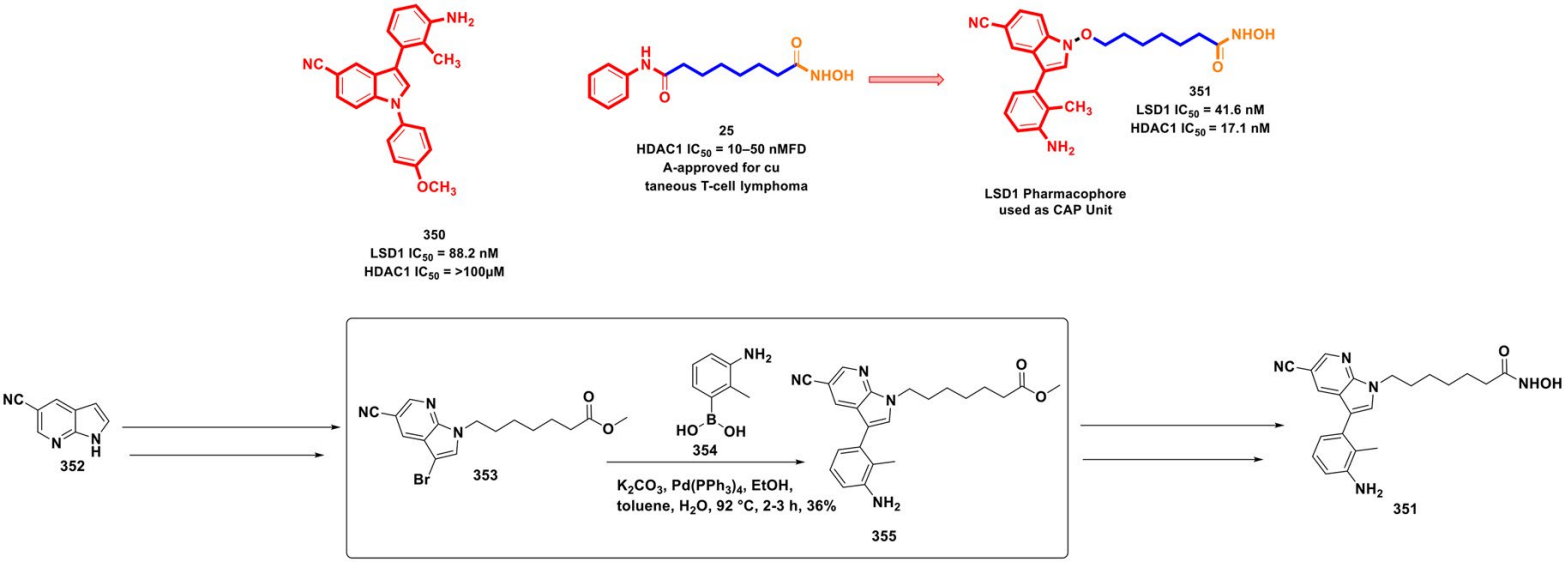
Design Strategy & Synthetic Route of Novel 5‑Cyano-3-phenylindole-Based LSD1/HDAC Dual Inhibitors (the figure was drawn by the authors using chemdraw software).

Duan *et al.* designed 5-cyano-3-phenylindole-based LSD1/HDAC dual inhibitors for colorectal cancer, inspired by the Novartis LSD1 inhibitor (**350**). Structural optimisation led to compound (**351**), exhibiting potent dual inhibition of LSD1 and HDAC isoforms (HDAC1–3, HDAC6) and >10-fold stronger antiproliferative activity than SAHA in HCT-116 and HT-29 cells. Mechanistic studies confirmed epigenetic modulation through increased H3/H4 and α-tubulin acetylation and H3K4 methylation. The synthetic route began with the alkylation of an indole core (**352**), followed by bromination, which yielded a bromine intermediate (**353**). Later, Pd(PPh_3_)_4_-catalysed Suzuki–Miyaura cross-coupling between brominated indole (**353**) and substituted phenylboronic acid (**354**) under anhydrous conditions in toluene at 90 °C with K_2_CO_3_ base was performed. This palladium-catalysed step efficiently constructed the biaryl core (**355**), essential for linker integration and final hydroxamic acid formation, yielding the target molecule (36%) as reported in Figure 57^166^.

**Figure 58.**
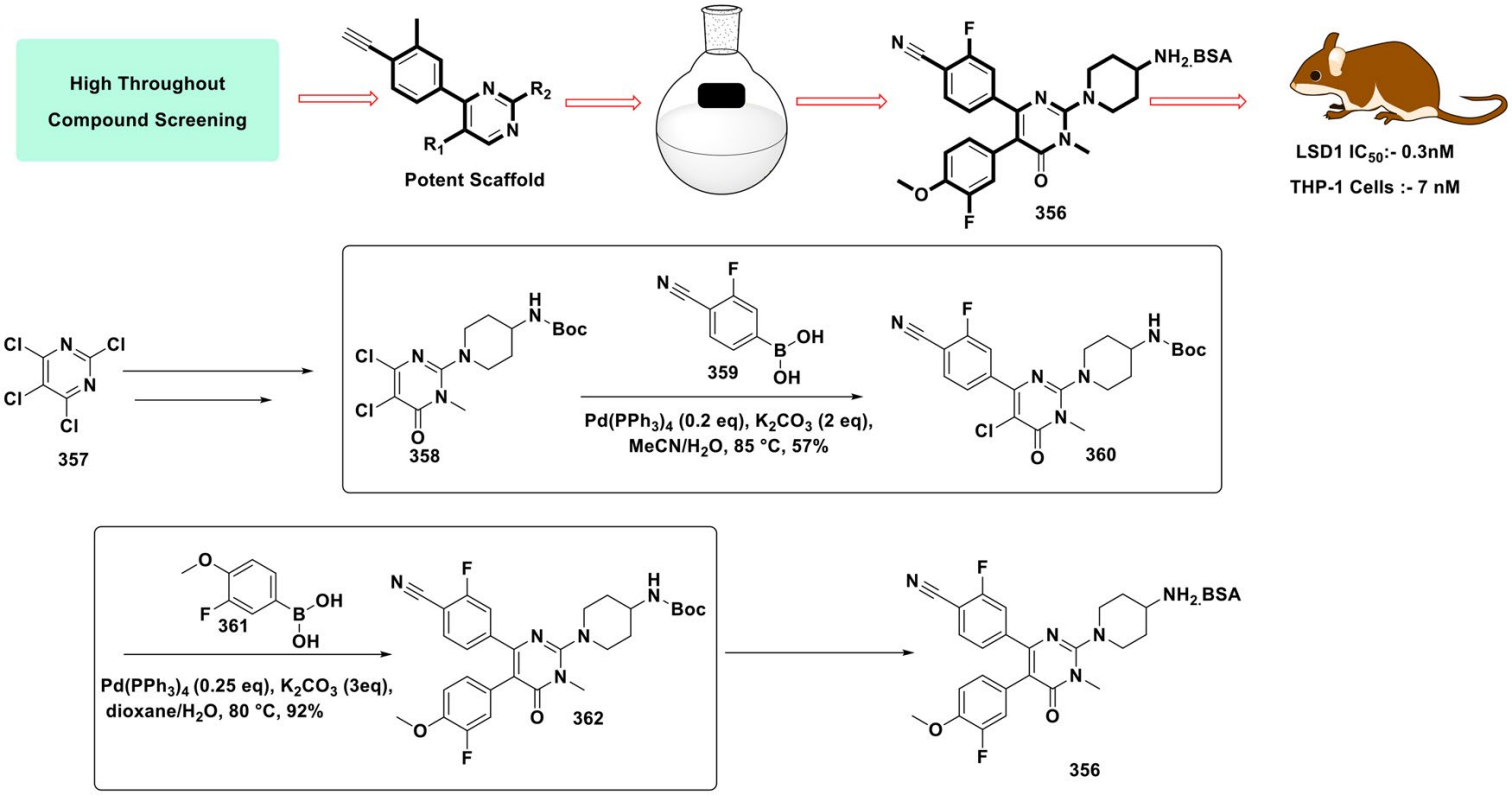
Design Strategy & Synthetic Route of CC-90011 (328): A Lysine Specific Demethylase 1 Inhibitors (the figure was drawn by the authors using chemdraw software).

In pursuit of expanding the activity spectrum of LSD1 inhibitors to numerous malignancies, Chen *et al*. performed high-throughput screening of 300,000 compounds, which led to the identification of 4-(pyrimidin-4-yl)benzonitrile as a crucial pharmacophore for LSD1 inhibition. Further optimisation of this scaffold yielded CC-90011 (**356**), which is a highly potent and selective LSD-1 inhibitor. It is important to mention that the synthesis of CC-90011 (**356**) commenced with commercially available perchloropyrimidine (**357**), proceeding through a six-step sequence. Initial regioselective displacement of the 4-chlorine atom in (**358**) using aqueous NaOH under mild conditions afforded pyrimidinone (**358**) in 69% yield. Two sequential Suzuki-Miyaura cross-couplings followed: the first with (4-cyano-3-fluorophenyl)boronic acid (**359**) in heated acetonitrile/water (85 °C) yielded intermediate (**360**) (57%), while the second with (3-fluoro-4-methoxyphenyl)boronic acid (**361**) in dioxane/water (80 °C) delivered (**362**) with high efficiency (92%). Final deprotection of (**362**) using bis(trimethylsilyl)acetamide (BSA) and formic acid provided CC-90011 (**356**) as a benzenesulfonate salt in 95% yield^167^. Mechanistically, it induced cellular differentiation in AML and SCLC models. It upregulated CD11b expression in THP-1 cells (EC_50_ = 7 nM), inhibited AML Kasumi-1 proliferation (EC_50_ = 2 nM), and showed minimal toxicity towards normal fibroblasts. During the *in vivo* studies, CC-90011 markedly suppressed tumour growth in both xenograft and PDX SCLC models (Figure 58)^167^.

## Inferences/observations – epigenetic drug discovery through palladium catalysis

Pd-catalysed reactions to form diverse C-C and C-N bonds have provided unparalleled versatility and precision to accomplish multistep synthetic routes of small molecule epigenetic inhibitors; however, despite the overwhelming success and widespread application of Pd-based methodologies, several challenges persist, particularly regarding suboptimal yields when complex heterocyclic or sterically hindered substrates are involved^51^^,^^168–170^. Hereby, we present a balanced and critical synthesis of the observations, not only focusing on yield but also on catalyst-ligand performance, substrate compatibility, solvent and base selection, and reaction conditions (Table 2). The success of Pd catalysis is far from universal because certain functional groups, particularly hydroxamates or poly-heteroaromatic systems such as purines, are prone to catalyst poisoning or decomposition under standard conditions, which often leads to poor yields (below 40%). For instance, hydroxamate-functionalised HDAC inhibitors synthesised using Pd(PPh_3_)_4_ yielded only 21% of product, which reflects the susceptibility of hydroxamic acids to degradation in basic and polar solvents^149^. Likewise, purine-linked scaffolds often sequester Pd centres through strong nitrogen coordination that forms catalytically inert off-cycle complexes^170^. These contrasting outcomes reveal the double-edged nature of substrate complexity as richly functionalised molecules are attractive for medicinal chemistry, but they also present formidable challenges in catalysis.

**Table 2.** List of different Chemical Scaffolds and their corresponding reaction system.

Chemical Scaffold Type	Pd Catalyst Used	Base	Solvent System	Yield (%)
*Purine-based hydroxamic acids*	PdCl₂(dppf)₂	K₂CO₃	Dioxane/H₂O	Moderate to high^114^^,^^118^
*Quinoxaline-pyrazole hybrids*	Pd(dppf)Cl₂, Pd₂(dba)₃ + BINAP	Cs₂CO₃	Dioxane/H₂O, Toluene	62–72^115^^,^^118^
*Indazole–pyridine hybrids*	Pd(dppf)Cl₂·CH₂Cl₂	K₂CO₃	DMF	∼58–80^116^^,^^123^
*Azaindolyl–sulphonamide derivatives*	Pd(OAc)₂ + PPh₃	TEA	DMF	∼37^138^
*Pyridone–dihydroisoquinoline-1(2H)-ones*	Pd(PPh₃)₄	CsF	1,4-Dioxane	∼73^152^
*LSD1 inhibitors (benzofuran/indole based)*	Pd(dppf)Cl₂, Pd(OAc)₂	KF, K₃PO₄	THF, DME	63–70^125^^,^^126^
*EZH2 PROTACs (biaryl systems)*	Pd(dppf)Cl₂	K₂CO₃	Dioxane/H₂O	74^165^
*BRD4/PLK1 dual inhibitors*	Pd₂(dba)₃ + XPhos	K₂CO₃	tert-Butanol	∼50^144^
*BET-HDAC bifunctional scaffolds*	Pd₂(dba)₃	Cs₂CO₃	Toluene, EtOH/H₂O	Moderate to high^145^
*DNMT1 inhibitors*	Pd(OAc)₂	K₂CO₃	THF	Moderate^139^
*HDAC6-selective inhibitors (acrylamide)*	Pd(OAc)₂ + PPh₃	TEA	DMF	37^142^

Furthermore, the choice of catalyst-ligand system also represents a decisive factor in the efficiency and selectivity of Pd-catalysed cross-coupling reactions (Table 3). Notably, bidentate ligands such as dppf have consistently delivered superior yields compared to monodentate systems. For example, in the Suzuki coupling of a sterically hindered aryl bromide with a boronic ester during the synthesis of an EZH2 inhibitor, a Pd(dppf)Cl_2_ catalyst ensured smooth transmetalation and afforded a 78% yield^124^. In contrast, Pd(PPh_3_)_4_, when employed under similar conditions with a less hindered substrate, gave only 51% yield, which might be due to premature catalyst degradation^166^. Similarly, in C-N bond formations, Pd_2_(dba)_3_ with BINAP facilitated the coupling of aryl chlorides and amines in yields more than 70%, whereas ligand-free systems resulted in lower products, probably due to more impurities and catalyst deactivation^154^^,^^171^.

**Table 3.** List of different Organopalladium Catalysts and their advantages.

Catalyst	Preferred Reactions	Ligand Type	Advantages
*Pd(dppf)Cl₂*	Suzuki (C–C), some Buchwald	Bidentate (dppf)	High yields, thermally stable, versatile
*Pd(PPh₃)₄*	Suzuki, Buchwald-Hartwig	Monodentate	Rapid oxidative addition, useful in aminations
*Pd₂(dba)₃*	Buchwald-Hartwig, C–N	Ligand-free or BINAP	Low loading, stable, high performance
*Pd(OAc)₂*	Heck, Suzuki (late stage)	Biaryl phosphine	Air-stable, good for electron-deficient substrates
*PdCl₂(PPh₃)₂*	Sonogashira, Heck	Monodentate	Easy ligand dissociation, flexible

Notably, dppf is most used but not universally suitable across different Pd-catalysed cross-coupling reactions. In cases involving electron-deficient halides or poly-heteroaromatic substrates, dppf-ligated Pd often fails to promote efficient activation, leading to incomplete conversions even after continuing the reaction for a long period of time. These outcomes highlight the need to tailor ligands’ steric and electronic properties to the specific demands of the substrate. For example, ligands such as X-Phos and RuPhos have demonstrated superior performance under these challenging conditions. In contrast, monodentate systems like Pd(PPh_3_)_4_ exhibit mixed utility: they are effective in relatively simple arylations; however, they often underperform in sterically hindered or base-sensitive environments, which might be due to their limited ability to stabilise Pd(0) under demanding conditions. Even more problematic are ligand-free systems (e.g., Pd(OAc)_2_ alone), as they are more prone to Pd black formation- a major deactivation pathway that prematurely terminates the catalysis. This phenomenon is especially pronounced in Heck reactions involving bulky aryl halides. In some instances, the use of co-catalysts conferred a solution. For example, in the Sonogashira coupling used for BRD4 degrader synthesis, the inclusion of CuI enhanced catalytic turnover, allowing the reaction to proceed efficiently at lower catalyst loadings and temperatures^132^. Mechanistically, these behaviours are rooted in the reactivity and instability of Pd(0). While Pd(0) is indispensable for oxidative addition due to its electron-rich and low-valent character, it remains highly unstable as it is more prone to oxidative degradation, aggregation into Pd black, and catalyst poisoning^172^^,^^173^. Commercial Pd(0) complexes (e.g., Pd(PPh_3_)_4_) provide convenience but are limited by air/moisture sensitivity, poor shelf life, and high cost, which restrict scalability at the bulk scale. For this reason, use of Pd(II) pre-catalysts appears to be a prudent tactic as they are air-stable, cost-effective, and readily reduced *in situ* to Pd(0) under reaction conditions^174^. Ligands stabilise Pd(0) once generated and modulate key steps in the catalytic cycle-oxidative addition, trans-metalation, and reductive elimination-through their steric and electronic effects. For example, electron-rich ligands (e.g., phosphines, N-heterocyclic carbenes) promote Pd(II) reduction via electron transfer, while bulky ligands (e.g., PtBu_3_, JohnPhos) destabilise Pd(II) intermediates and accelerate reductive elimination^175–180^.

Furthermore, catalyst loading and reaction temperature were also found to significantly influence the overall efficiency and outcome of cross-coupling reactions. In early-stage transformations, particularly those involving less sterically hindered or electronically activated substrates, low catalyst loadings (typically 1–2 mol%) were sufficient to achieve high conversions. For example, Buchwald-Hartwig amination was used to synthesise BET inhibitor. In the aforementioned reaction, the use of Pd_2_(dba)_3_ (1 mol%) in combination with BINAP as a ligand afforded the desired product in high yield^143^. However, in late-stage functionalizations, where substrates often exhibit increased steric bulk or complex substitution patterns, higher catalyst loadings were necessary to drive the reaction to completion. In such cases, increasing the palladium catalyst to 5 mol% notably improved conversion and yield. A case in point is the synthesis of CC-90011, where Pd(PPh_3_)_4_ was employed at different stages. In the initial steps, lower catalyst loadings were sufficient due to the relatively simple substrate framework. However, as the molecule progressed towards its final, pharmacologically active form (a more hindered and electronically diverse structure), a higher palladium loading was required to maintain reaction efficiency^167^. Moreover, the impact of both catalyst concentration and temperature can also pose challenges in late-stage synthesis and can be optimised during the initial days. The temperature range generally varies from 80–110 °C, but certain reactions, like the synthesis of acrylate intermediate using Heck coupling, require 160 °C for 16h to get the desired outcome^131^.

At the industrial scale, despite their broad utility, Pd-based systems face critical barriers because homogeneous catalysis requires high Pd loadings that ultimately increase costs, metal contamination, and toxic waste, all of which are tightly regulated in pharmaceutical production^181^. To overcome these limitations, efforts need to be directed towards reducing the Pd loadings to ppm levels while retaining its efficiency. This can be achieved through the development of highly active ligands such as HandaPhos, t-BuXPhos, EvanPhos, and N2Phos, which accelerate key catalytic steps under mild conditions^168^^,^^182–185^. Notably, Yang *et al.* demonstrated Suzuki-Miyaura couplings using only 0.001 mol% [(bdppma)PdCl_2_], affording more than 95% yield, while bisphosphine-grafted amphiphilic polymers produced Pd nanoparticles that enabled couplings with 96% yield at 100 ppm Pd^186^. Similarly, MOF-anchored Pd catalysts achieved up to 97% yield in Heck couplings with 0.5 mol% Pd loading, illustrating the effectiveness of ligand and support engineering^187^^,^^188^. Recent advances illustrate the power of ligand design to employ low catalyst loading and more outputs in diverse environments, for example:

- Pd(COD)Br_2_ with t-Bu_2_(4-Me_2_C_6_H_4_)P afforded a 94% yield in Sonogashira coupling.^189^
- N2-Phos with Pd(OAc)_2_ enabled catalyst loadings as low as 1000–2500 ppm while maintaining excellent yields.^185^
- SNS pincer ligands with PdI_2_ reduced catalyst loading to 1.5 mol% yet delivered >99% yield in Heck reactions.^190^
- Tang’s bisphosphine with Pd_2_(dba)_3_ achieved 99% yield in dienyl anion transformations.^191^
- Substituted pyridine ligands with Pd(II) salts improved stability and efficiency in both Suzuki–Miyaura and Heck couplings.^192^.

Beyond single-ligand systems, mixed-ligand architectures are also emerging as a powerful strategy. For example, an NHC-phosphine hybrid with PdCl_2_ produced a stable complex that enabled sequential one-pot Suzuki couplings at 10 mol% loading^51^^,^^175^^,^^176^. Beyond ligand design, heterogeneous Pd systems on carbon, polymers, or inorganic supports further enhance recyclability and sustainability, while nanostructured catalysts maximise surface area and turnover efficiency. For example, PEG-silica-Pd complexes maintained ∼89% yield in Suzuki couplings across multiple cycles without leaching, while Pd/C showed excellent reusability. Notably, silica-Pd(0) systems functionalised with β-cyclodextrin-azobenzene extended Pd catalysis to light-controlled biorthogonal reactions in living cells^193–197^. Together, these strategies demonstrate that Pd catalysis can be tuned to address both the scientific challenges of substrate complexity and the practical demands of industrial scalability. The trajectory of the field thus moves from laboratory success with homogeneous Pd systems towards low-loading, recyclable, and sustainable catalytic technologies that meet the future needs of pharmaceutical manufacturing.

Importantly, reaction performance is not only dictated by the ligand-catalyst pairing alone: bases and solvents critically influence the Pd(II) → Pd(0) conversion and overall reactivity^198^. In a systematic study, Fantoni *et al.* demonstrated that efficient Pd(0) generation requires co-optimisation of ligands, bases, and solvents, rather than focusing on ligands in isolation^23^. Mixed solvent systems such as 1,4-dioxane/H_2_O are widely employed and often deliver optimal results in Suzuki-Miyaura couplings. However, their limited miscibility with highly polar reactants can sometimes compromise reaction homogeneity. To address such solubility and reactivity challenges, researchers have evaluated alternative solvents such as acetonitrile/water, ethanol/DME, and ethanol/toluene, tailoring conditions to substrate properties and desired outcomes.

Notably, base selection is equally critical (Table 4). Mild inorganic bases such as K_2_CO_3_ and Cs_2_CO_3_ are often favoured because they promote efficient deprotonation without inducing substrate degradation. Similarly, other bases, including Na_2_CO_3_, K_3_PO_4_, NaHCO_3_, and KF, are employed depending on compatibility with the substrate and mechanistic requirements. In more demanding systems, stronger bases such as NaOH, TEA, or t-BuONa are sometimes used to enhance nucleophilicity and enable challenging bond formations, though their increased reactivity can lead to side reactions with labile functional groups such as esters and hydroxamic acids. Careful optimisation, however, has enabled their successful application in selective pharmacophore synthesis. Typically, base equivalents fall in the 1.5–5 range, though specific protocols such as the one for the synthesis of dual SHP2/HDAC inhibitors employed up to 10 equivalents of K_3_PO_4_ to maximise yields or to suppress side-product formation^117^. Despite these refinements, traditional Pd-catalysed protocols still face significant limitations, like widespread use of toxic organic solvents (e.g., DMF, DMSO, toluene), reliance on non-recyclable homogeneous Pd catalysts, and the need for high temperatures and laborious purification steps contribute to waste generation, environmental hazards, and high production costs. Moreover, the scarcity and high cost of Pd pose major challenges for scaling these reactions for industrial applications^199^^,^^200^.

**Table 4.** List of bases and solvent systems, which are commonly used with organopalladium catalysts.

Base	Solvent	Effect on Yield
*K₂CO₃*	Dioxane/H₂O	Best general system. Excellent for Suzuki (65–87%)^159^^,^^160^
*Cs₂CO₃*	Toluene or DMF	Preferred in Buchwald reactions. High reactivity^115^^,^^155^
*KF*	THF	Used in selected cases for boron activation^125^
*NaOAc/KOAc*	Dioxane/EtOH/H₂O	Mild base systems for sensitive moieties^120^
*Et₃N*	DMF	Effective for Heck couplings^142^

To address these issues, recent efforts have focused on developing greener and more sustainable catalytic systems. A key strategy involves replacing toxic solvents with aqueous and bio-based alternatives such as water and ethanol, which enable Pd-catalysed reactions at ppm-level loadings. The use of surfactants such as TPGS-750-M has further enabled high-yielding couplings (up to 99%) in water, demonstrating the potential of micellar catalysis^201^. In parallel, heterogeneous catalysts, solid-supported systems, and nanostructured Pd materials have emerged as effective solutions to minimise Pd leaching, enhance recyclability, and lower overall metal usage^202^. Complementary strategies for homogeneous catalyst recovery, such as organic solvent nanofiltration, have also shown promise; for example, Xiao *et al.* achieved up to five reuse cycles of Pd(dba)_2_ in the synthesis of AZD4625^203^. Furthermore, ligand-free and base-free protocols are being developed to minimise auxiliary reagents, reduce waste, and simplify product purification. Finally, the integration of microwave-assisted and continuous-flow technologies offers further opportunities to improve efficiency while reducing energy input. Flow-based Pd-catalysed couplings have already demonstrated shorter reaction times, lower catalyst loadings, and enhanced scalability, providing practical routes for greener industrial synthesis^171^^,^^204^^,^^205^.

Hence, the evolution of Pd catalysis highlights a clear trajectory: from early reliance on unstable Pd(0) complexes and non-optimised ligand systems, to modern strategies emphasising Pd(II) precatalysts, ligand innovation, and reaction environment co-tuning. Moving forward, systematic exploration of ligand diversity-including mixed architectures and their interplay with bases and solvents, will be central to enhancing catalyst stability, selectivity, and scalability, thereby broadening the industrial applicability of Pd-catalysed cross-couplings for the construction of small molecules.

## Conclusion

Epigenetic dysregulation has emerged as a defining hallmark of cancer, supporting the malignant transformation of tumours, progression, and therapeutic resistance. Numerous small-molecule epigenetic inhibitors, which target different epigenetic enzymes such as HDACs, DNMTs, BET proteins, and EZH2, have therefore emerged as potent therapeutic modalities, with several already achieving FDA approval for different malignancies. Despite these developments, the clinical results of various epigenetic inhibitors remain hindered by some limitations, including selectivity, metabolic stability, bioavailability, and tissue distribution, highlighting the urgent need to refine their structural frameworks to optimise the pharmacodynamic and pharmacokinetic properties of chemotherapeutic drugs. In this context, the literature precedents have demonstrated that palladium catalysis plays a vital role in medicinal chemistry campaigns focused on the generation of chemical architectures capable of addressing epigenetic targets. It highlights palladium-catalysed cross-coupling as an enabling technology for translating epigenetic target hypotheses into chemically tractable and biologically testable small molecules. The unique properties of different palladium complexes facilitate highly selective C-C and C-N bond formations, which have revolutionised the synthesis of structurally complex, drug-like scaffolds, including hydroxamic acids, and heteroaromatic frameworks that serve as the core of modern epigenetic inhibitors. Indeed, the modularity and versatility of organopalladium catalysis not only streamline synthetic competence but also expand the structure libraries of drug-like scaffolds for structure-activity relationship studies. Moreover, the organopalladium catalysis gains high value in the synthesis of dual and polypharmacological inhibitors that simultaneously inhibit multiple epigenetic pathways and produce synergistic antitumor effects. At present, the synthetic tool is endowed with numerous palladium catalysis, which are the prominent choice for the drug discovery team to design complex chemical architecture and conduct comprehensive optimisation of the synthetic route of therapeutic scaffold. From this review, we gain insights into drug discovery endeavours centred on epigenetics, which have been clearly presented in terms of both organic and medicinal chemistry, particularly in the context of palladium catalysis, catalyst loading, ligand selection, base selection, and solvent choice. The interplay between scaffold complexity, catalyst-ligand design, base strength, and solvent environment emerges as a decisive determinant of synthetic success, particularly in the late-stage functionalization of pharmacologically advanced molecules. Advances in low-loading, ligand-engineering, and heterogeneous Pd systems highlight a clear path towards scalable, greener, and industrially compliant synthesis. From a medicinal chemistry perspective, these innovations not only expand accessible chemical space but also enable systemic structure-activity optimisation of epigenetic drug candidates. In a nutshell, it is highly anticipated that palladium catalysis with green chemistry principles, data-driven catalyst optimisation, and continuous-flow synthesis will gain more prominence in the field of drug discovery in the coming years, which will be helpful in enhancing the size of the structural pool of small molecule chemotypes as anticancer agents.

## Data Availability

The authors confirm that the data supporting the findings of this study are available within the article.
